# Potential and limitations of machine meta-learning (ensemble) methods for predicting COVID-19 mortality in a large inhospital Brazilian dataset

**DOI:** 10.1038/s41598-023-28579-z

**Published:** 2023-03-01

**Authors:** Bruno Barbosa Miranda de Paiva, Polianna Delfino Pereira, Claudio Moisés Valiense de Andrade, Virginia Mara Reis Gomes, Maira Viana Rego Souza-Silva, Karina Paula Medeiros Prado Martins, Thaís Lorenna Souza Sales, Rafael Lima Rodrigues de Carvalho, Magda Carvalho Pires, Lucas Emanuel Ferreira Ramos, Rafael Tavares Silva, Alessandra de Freitas Martins Vieira, Aline Gabrielle Sousa Nunes, Alzira de Oliveira Jorge, Amanda de Oliveira Maurílio, Ana Luiza Bahia Alves Scotton, Carla Thais Candida Alves da Silva, Christiane Corrêa Rodrigues Cimini, Daniela Ponce, Elayne Crestani Pereira, Euler Roberto Fernandes Manenti, Fernanda d’Athayde Rodrigues, Fernando Anschau, Fernando Antônio Botoni, Frederico Bartolazzi, Genna Maira Santos Grizende, Helena Carolina Noal, Helena Duani, Isabela Moraes Gomes, Jamille Hemétrio Salles Martins Costa, Júlia di Sabatino Santos Guimarães, Julia Teixeira Tupinambás, Juliana Machado Rugolo, Joanna d’Arc Lyra Batista, Joice Coutinho de Alvarenga, José Miguel Chatkin, Karen Brasil Ruschel, Liege Barella Zandoná, Lílian Santos Pinheiro, Luanna Silva Monteiro Menezes, Lucas Moyses Carvalho de Oliveira, Luciane Kopittke, Luisa Argolo Assis, Luiza Margoto Marques, Magda Cesar Raposo, Maiara Anschau Floriani, Maria Aparecida Camargos Bicalho, Matheus Carvalho Alves Nogueira, Neimy Ramos de Oliveira, Patricia Klarmann Ziegelmann, Pedro Gibson Paraiso, Petrônio José de Lima Martelli, Roberta Senger, Rochele Mosmann Menezes, Saionara Cristina Francisco, Silvia Ferreira Araújo, Tatiana Kurtz, Tatiani Oliveira Fereguetti, Thainara Conceição de Oliveira, Yara Cristina Neves Marques Barbosa Ribeiro, Yuri Carlotto Ramires, Maria Clara Pontello Barbosa Lima, Marcelo Carneiro, Adriana Falangola Benjamin Bezerra, Alexandre Vargas Schwarzbold, André Soares de Moura Costa, Barbara Lopes Farace, Daniel Vitorio Silveira, Evelin Paola de Almeida Cenci, Fernanda Barbosa Lucas, Fernando Graça Aranha, Gisele Alsina Nader Bastos, Giovanna Grunewald Vietta, Guilherme Fagundes Nascimento, Heloisa Reniers Vianna, Henrique Cerqueira Guimarães, Julia Drumond Parreiras de Morais, Leila Beltrami Moreira, Leonardo Seixas de Oliveira, Lucas de Deus Sousa, Luciano de Souza Viana, Máderson Alvares de Souza Cabral, Maria Angélica Pires Ferreira, Mariana Frizzo de Godoy, Meire Pereira de Figueiredo, Milton Henriques Guimarães-Junior, Mônica Aparecida de Paula de Sordi, Natália da Cunha Severino Sampaio, Pedro Ledic Assaf, Raquel Lutkmeier, Reginaldo Aparecido Valacio, Renan Goulart Finger, Rufino de Freitas, Silvana Mangeon Meirelles Guimarães, Talita Fischer Oliveira, Thulio Henrique Oliveira Diniz, Marcos André Gonçalves, Milena Soriano Marcolino

**Affiliations:** 1 Computer Science Department, Universidade Federal de Minas Gerais, Av. Presidente Antônio Carlos, 6627, Belo Horizonte, Brazil; 2 Universidade Federal de Minas Gerais, Av. Presidente Antônio Carlos, 6627, Belo Horizonte, Brazil; 3 Institute for Health Technology Assessment (IATS/ CNPq), R. Ramiro Barcelos, 2359, building 21, room 507, Porto Alegre, Brazil; 4 Medical School and University Hospital, Universidade Federal de Minas Gerais, Av. Professor Alfredo Balena, 190, room 246, Belo Horizonte, Brazil; 5 Universidade Federal de São João del-Rei, R. Sebastião Gonçalves Coelho, 400, Divinópolis, Brazil; 6 Department of Statistics, Universidade Federal de Minas Gerais, Av. Presidente Antônio Carlos, 6627, ICEx, room 4071, Belo Horizonte, Brazil; 7 Faculdade de Ciências Médicas de Minas Gerais, Al. Ezequiel Dias, 275, Belo Horizonte, Brazil; 8 Hospital UNIMED BH, Av. Do Contorno, 3097, Belo Horizonte, Brazil; 9 Hospital Risoleta Tolentino Neves, R. das Gabirobas, 01, Belo Horizonte, Brazil; 10 Hospital São João de Deus, R. do Cobre, 800, São João de Deus, Brazil; 11 Hospital Regional Antônio Dias, R. Maj. Gote, 1231, Patos de Minas, Brazil; 12 Hospital Santo Antônio, Pç. Dr. Márcio Carvalho Lopes Filho, 501, Curvelo, Brazil; 13 Hospital Santa Rosália, R. Dr. Onófre, 575, Teófilo Otoni, Brazil; 14 Faculdade de Medicina de Botucatu-Universidade Estadual Paulista “Júlio de Mesquita Filho”, Av. Prof. Mário Rubens Guimarães Montenegro, s/n-UNESP-Campus de Botucatu, Botucatu, Brazil; 15 Hospital SOS Cárdio, Rod. SC-401, 121, Florianópolis, Brazil; 16 Hospital Mãe de Deus, R. José de Alencar, 286, Porto Alegre, Brazil; 17 Hospital de Clínicas de Porto Alegre, R. Ramiro Barcelos, 2350, Porto Alegre, Brazil; 18 Hospital Nossa Senhora da Conceição and Hospital Cristo Redentor, Av. Francisco Trein, 326, Porto Alegre, Brazil; 19 Hospital Julia Kubitschek, R. Dr. Cristiano Rezende, 2745, Belo Horizonte, Brazil; 20 Hospital Santa Casa de Misericórdia de Belo Horizonte, Av. Francisco Sales, 1111, Belo Horizonte, Brazil; 21 Universidade Federal de Santa Maria/Hospital Universitário/EBSERH, Av. Roraima, 1000, building 22, Santa Maria, Brazil; 22 Hospital Márcio Cunha, Av. Kiyoshi Tsunawaki, 48, Ipatinga, Brazil; 23 Hospital Semper, Al. Ezequiel Dias, 389, Belo Horizonte, Brazil; 24 Hospital Metropolitano Odilon Behrens, R. Formiga, 50, Belo Horizonte, Brazil; 25 Universidade Federal da Fronteira Sul, Av. Fernando Machado, 108E, Chapecó, Brazil; 26 Hospital João XXIII, Av. Professor Alfredo Balena, 400, Belo Horizonte, Brazil; 27 Hospital São Lucas PUCRS, Av. Ipiranga, 6690, Porto Alegre, Brazil; 28 Hospital Bruno Born, Av. Benjamin Constant, 881, Lajeado, Brazil; 29 Hospital Luxemburgo, R. Gentios, 1350, Belo Horizonte, Brazil; 30 Hospital Universitário Ciências Médicas, R. dos Aimorés, 2896, Belo Horizonte, Brazil; 31 Pontifícia Universidade Católica de Minas Gerais, Av. Dom José Gaspar, 500, Belo Horizonte, Brazil; 32 Hospital Moinhos de Vento, R. Ramiro Barcelos, 910, Porto Alegre, Brazil; 33 Moinhos Research Institute, 910 Ramiro Barcelos Street, 5 floor, Porto Alegre, Brazil; 34 Fundação Hospitalar do Estado de Minas Gerais–FHEMIG, Cidade Administrativa de Minas Gerais, Edifício Gerais, 13rd floor, Rod. Papa João Paulo II, 3777, Belo Horizonte, Brazil; 35 Hospitais da Rede Mater Dei, R. Gonçalves Dias, 2700, Belo Horizonte, Brazil; 36 Hospital Eduardo de Menezes, R. Dr. Cristiano Rezende, 2213, Belo Horizonte, Brazil; 37 Hospital Tacchini, R. Dr. José Mário Mônaco, 358, Bento Gonçalves, Brazil; 38 Instituto Orizonti, Pç. Engenheiro Flávio Gutierrez, Belo Horizonte, Brazil; 39 Hospital das Clínicas da Universidade Federal de Pernambuco, Av. Prof. Moraes Rego, 1235, Recife, Brazil; 40 Hospital Santa Cruz, R. Fernando Abott, 174, Santa Cruz do Sul, Brazil; 41 Hospital Metropolitano Doutor Célio de Castro, R. Dona Luiza, 311, Belo Horizonte, Brazil; 42 Hospital Universitário Canoas, Av. Farroupilha, 8001, São José, Canoas, Brazil; 43 Universidade Federal de Ouro Preto, R. Dois, Ouro Preto, Brazil; 44 Hospital Regional do Oeste, R. Florianópolis, 1448 E, Chapecó, Brazil; 45 Telehealth Center, University Hospital, Universidade Federal de Minas Gerais, Avenida Professor Alfredo Balena, 110 room 107. Santa Efigênia, Belo Horizonte, MG CEP 30130-100 Brazil

**Keywords:** Infectious diseases, Computational science

## Abstract

The majority of early prediction scores and methods to predict COVID-19 mortality are bound by methodological flaws and technological limitations (e.g., the use of a single prediction model). Our aim is to provide a thorough comparative study that tackles those methodological issues, considering multiple techniques to build mortality prediction models, including modern machine learning (neural) algorithms and traditional statistical techniques, as well as meta-learning (ensemble) approaches. This study used a dataset from a multicenter cohort of 10,897 adult Brazilian COVID-19 patients, admitted from March/2020 to November/2021, including patients [median age 60 (interquartile range 48–71), 46% women]. We also proposed new original population-based meta-features that have not been devised in the literature. *Stacking* has shown to achieve the best results reported in the literature for the death prediction task, improving over previous state-of-the-art by more than 46% in Recall for predicting death, with AUROC 0.826 and MacroF1 of 65.4%. The newly proposed meta-features were highly discriminative of death, but fell short in producing large improvements in final prediction performance, demonstrating that we are possibly on the limits of the prediction capabilities that can be achieved with the current set of ML techniques and (meta-)features. Finally, we investigated how the trained models perform on different hospitals, showing that there are indeed large differences in classifier performance between different hospitals, further making the case that errors are produced by factors that cannot be modeled with the current predictors.

## Introduction

Although over 11 billion doses of coronavirus disease 2019 (COVID-19) vaccines have been administered worldwide, wide swaths of unvaccinated people (due to an uneven and slow rollout, as well as anti-vaccine movements) could allow the virus to further mutate and potentially spawn more transmissible and increasingly deadly variants. All of this means that COVID-19 is still an issue that governments worldwide will need to keep grappling with^1,2^.

Given this scenario, there is an urgent need for disease stratification tools upon hospital admission, to allow early identification of risk of death in COVID-19 patients, assisting in the management of disease and optimizing resource allocation, hopefully assisting to save lives. Although several models and scores, based on traditional statistical methods and/or artificial intelligence (AI), have been proposed^3–7^, the majority present methodological flaws and technological limitations. Indeed the majority of previous studies: (i) rely on limited sample sizes; (ii) lack consideration of covariate correlations (between the probability of the prediction and the accuracy), external validation, or systematic evaluation of multiple models; (iii) use inadequate evaluation metrics, and/or a small number of predictors, and, finally (iv) exploit **at most a single model** for the prediction task. All these issues mean that effective and reliable prognostic prediction models are still in need^3,8,9^.

In this context, the contributions of this article are **manyfold**. *First,* we address most of the methodological flaws of previous work, while exploiting a large dataset from a multicenter cohort on Brazilian COVID-19 patients, admitted from March 2020 to November 2021, with 10,897 patients (median age 60 [interquartile range 48–71], 46% women). *Second*, we provide a comparative study of multiple techniques to build prediction models for mortality, including modern ML (neural) algorithms and traditional statistical techniques, as well as meta-learning (ensemble) approaches, many of which had never been applied to this task. *Third*, we propose new original population-based meta-features that, to the best of our knowledge, have not been exploited in any other work in the literature for death prediction. *Fourth*, we provide an in-depth discussion of our results regarding effectiveness, explainability and reliability. *Fifth*, we conduct an extensive investigation of the predictive power of the base features as well as the (new) population and classifier-based meta-features at the Stacking level. *Sixth*, we analyze how our models’ performance behaves in each of the hospitals included in our study, a type of evaluation rarely performed, due to data unavailability.

Our extensive experimental evaluation demonstrates that Stacking can achieve the best results reported in the literature for the death prediction task, while the use of meta-features can help improve results considerably, particularly when applied to simpler techniques like Least Absolute Shrinkage and Selection Operator (*LASSO regression*). Our In-depth analysis of the Stacking errors also shows large differences in prediction rates among hospitals. The analysis reveals that mortality may be largely dependent on variables external to the patient, such as which hospital performs the care, possibly due to factors such as differences in therapeutic approach, expertise and experience of team members, among others.

The rest of this article is organized as follows. “Related work” section reviews the literature on other prediction models for COVID-19 mortality, using artificial intelligence techniques or traditional statistical methods. This is followed by the presentation of the “Experimental methodology” section, and the “Results and discussion” section. “Conclusions” section concludes the article.

## Related work

Although an increasing number of prediction models have been proposed for the early assessment of the prognosis of COVID-19 patients, there is still a lot of work that needs to be done at the level of COVID-19 prognostic models design.

In this context, AI techniques enable the rapid and efficient discovery of insights across large heterogeneous populations. In addition, an algorithmic approach provides an objective evaluation and can capture nonlinear interactions that hardly are observed in the medical analyses^4^. Therefore, there is potential for improved results, especially taking into account the large datasets available at this point of the pandemic.

An exploratory search in Medline and MedRxiv databases (details of the search strategy are provided in the Supplementary Material) identified 214 studies. They used a broad range of analytical approaches to stratify patients by their mortality risk upon admission (Table S1). The existing literature largely focuses on European, American and Chinese hospitals, which are represented by 75.70% of the studies. However, models validated in only one country cannot be extrapolated to the global population, since there is heterogeneity among countries in different characteristics such as populations features (including genetics, race, ethnicity, prevalence of comorbidities), socioeconomic factors, access to healthcare, and their healthcare systems (hospitals’ patient load, practice and available resources)^10^.

Another important point is the sample size. The evaluation of a larger population allows certain metrics of model performance to be estimated with more accurate and reliable results. In contrast, smaller samples reduce the ability to identify risk factors and increase the likelihood of overfitting^11^. Among the analyzed models, 13.02% were developed and validated with a modest sample of 500–1000 patients, and 42.99% used even a smaller sample, with less than 500 patients. Less than half (32.55%) of the studies used a sample with more than 1000 patients. In this study, we had the asset of working with a large dataset of 10,897 patients, while previous studies with this size (> 10,000 patients) are limited (only 10.69% of the total).

Most of the studies (72.42%) used only traditional statistical methods, including multivariate logistic regression, LASSO and Cox regression analysis. Artificial intelligence techniques were used in 26.04% of the studies, among them machine learning stood out, including random forest (RF), XGBoost and SVM. Only a very small percentage of the studies used modern neural network methods in their studies as we did in ours. Most importantly, basically all studies exploited **at most a single technique** (either based on traditional statistics or in machine learning) for the prediction task. We on the other hand, exploit, compare and combine multiple ML techniques by means of a specific meta-learning (ensemble) technique—Stacking^12^—that learns how to better combine multiple models.

Overall, the majority of the developed models are limited by methodological bias, for example, with the absence of external validation in 72.89%, thus the assessment of accuracy in such studies may be overestimated. Less than a quarter (around 21.49%) reported having followed the methodological recommendations from the Transparent Reporting of a multivariable prediction model for Individual Prognosis Or Diagnosis (TRIPOD)^11^.

Another issue is that most studies used a patient sample from an early time in the pandemic course. Only eleven studies (5.14%) included patients after 2020, which means that most models lack generalizability to a newer sample of COVID-19 patients, after the development of vaccines and other therapeutic advancements. Our study included patients up until November, 2021 meaning that we could contemplate the diversity in the patients’ clinical characteristics by including two different pandemic moments (before and after large-scale immunization).

The model performance was evaluated in most studies by measuring the area under the curve (AUC). The mean AUC for the training set ranged from 0.63 to 0.99 for traditional statistical methods, and 0.72–0.99 for models using AI techniques. However, due to the very high skewness of the datasets (i.e. mortality corresponds to a very low percentage of the cases in the datasets, in other words, the non-death class dominates the distribution) neither AUC nor accuracy are adequate metrics^13^.

To properly assess the performance of different models, it is of utmost importance to use other metrics that consider imbalance issues, such as macro-average F1-score (macro-F1), used in 5.14% of studies. For example, Li et al.^14^ developed a deep-learning model and a risk-score system based on 55 clinical variables and observed that the most crucial biomarkers distinguishing patients at mortality imminent risk, were age, lactate dehydrogenase, procalcitonin, cardiac troponin, C-reactive protein, and oxygen saturation. The deep-learning model predicted mortality with an AUC of 0.852 and 0.844, for the training and the testing sets respectively, which is considered excellent. However, the performance of the proposed algorithm on training and testing datasets measured by the F1-score dropped to 0.642 and 0.616.

Few studies (Table S1) deeply analyzed the impact of the variables in the final model or on the final model outcome. Notably, Ikemura et al.^15^ used SHAP-values to analyze feature-importance. Additionally, most studies did not investigate how reliable the made predictions are in terms of the correlation between the probability of the prediction and the accuracy. This analysis has implications on the practical use of this technology. An accurate but unreliable method has its practical applicability diminished. We explicitly tackle these issues in our study.

Indeed, ours and Ikemura et al.^15^ are the only works in the literature that show that a particular combination strategy—Stacking—that learns how to combine multiple methods—can produce effectiveness results that can beat the best single ML method, considering evaluation metrics such as F-score and AUROC. Both studies also provide interpretability analyses regarding which variables/features (e.g., vital signs, biomarkers, comorbidities, etc.) are the most influential in generating an accurate model. Despite similarities, there are key differences between theirs and our work. We, for instance, propose new original population-based and information theoretic based meta-features that have not been exploited in any other work in the literature, for the problem of COVID-19 death prediction at admission time. Our analyses indeed show that the new proposed meta-features have much higher prediction capabilities than the base (patient predictors) features. The application of these meta-features to this problem is an original contribution. Furthermore, Ikemura et al. included patients from the early phase of the pandemic only, from March 1 to July 3, 2020. We have included patients from March 2020 to November 2021. Finally, our interpretability analyses of the predictive capability of the features consider both the base (patient) and the new meta-features, while Ikemura et al. consider only the base features. This type of joint interpretability analysis at the meta-level (i.e. Stacking level) along with the base features is unheard in the literature.

## Experimental methodology

### Study design

This is a substudy of the Brazilian COVID-19 Registry, a multi-hospital cohort study previously described in^16^. All protocols were approved by the National Commission for Research Ethics (CAAE: 30350820.5.1001.0008). The development, validation, and reporting of the models followed guidance from the Transparent Reporting of a Multivariable Prediction Model for Individual Prediction or Diagnosis (TRIPOD) checklist and the Prediction model Risk Of Bias Assessment Tool (PROBAST)^11,17^.

### Study participants

Consecutive adult patients with laboratory-confirmed COVID-19^18^ admitted consecutively in any of the 39 participating hospitals from March/2020 to November/2021 were enrolled. Individuals transferred between hospitals, and those with unavailable data from the first or last hospitals were excluded, as well as those admitted for other reasons but developed COVID-19 symptoms during their stay. The study protocol has been previously published^9^. In total, 10,897 adult Brazilian COVID-19 patients (median age 60 [interquartile range 48–71], 46% women) were included.

### Data collection (patient features)

Trained hospital staff or interns collected medical data using Research Electronic Data Capture (REDCap) tools^19^. Variables used to develop the models were obtained at hospital presentation. A set of potential predictor features for in-hospital mortality was selected a priori, as recommended, including comorbidities, lifestyle habits, clinical assessment and laboratory data upon hospital admission: age; days from symptom onset; heart and respiratory rate, mechanical ventilation, oxygen inspiration fraction, platelets, urea, C-reactive protein, lactate, gasometry results (pH, pO_2_, pCO_2_, bicarbonate), hemogram parameters (hemoglobin, neutrophils, lymphocytes, neutrophils to lymphocytes ratio, platelets) and sodium upon hospital admission. For more details, see Supplementary Material S1^9,11^. We call these *patient* or *base* features (we will use both terms interchangeably), to contrast with other meta-features used in the Stacking and derived from population information, as described later.

To ensure data quality, comprehensive data checks were undertaken. Error checking code was developed in R, to identify data entry errors, as previously described^9^. The results were sent to each center for checking and correction before further data analysis, model development and validation.

### Outcome

The outcome of interest was in-hospital mortality from any cause.

### Meta-features

In order to push the performance limits of our models, besides the base (patient) features, we experimented with the creation of novel artificial (meta-)features (we call these new features meta-features because they are derived from other base features or from the application of ML models on them), which strive to make classes more separable. We experimented with two main types in the meta-models (ensembles), namely features derived from the population (population-based meta-features) and features derived from the output of classifiers (stacking-based features).

In more detail, for the Stacking (ensemble) models, we used a combination (meta-)model that learns how to better combine class probabilities from other models. The overall idea from this type of meta-feature is that, if classifiers are at least partially independent, for instance, due to sampling or different classification premises (e.g., probabilistic, geometric, etc.), their predictions will more likely be correct for different instances, resulting in an overall combined classification that will be more likely correct. For instance, in a binary classification problem, an ensemble of three completely independent, better-than-random classifiers (i.e. errors are never on the same instances), for any given instance there will be at least two correct decisions. This means that, even with weak learners, such a combined ensemble would potentially be a very strong classifier. The problem lies in how to effectively make the classifiers independent in such a manner, since for all practical purposes, completely independent classifiers are just an abstract concept. We, indeed, can strive to make more independent classifiers, for instance, using different models with different classifications premises, and even using a learnable combination strategy.

The other type of meta-feature we propose is population-based features, such as the lethality on the top-100 most similar patients, death-entropy on the top-100 most similar patients and age-entropy on the top-100 most similar patients (always calculated within the training set). The overall idea of this kind of meta-feature is to compare any given individual to the training population. It is one thing to pass an ‘age’ feature to a learning algorithm, but a completely different one, to pass an ‘age’ percentile, which scales that age feature with regards to the population. In a similar fashion, for patient P_i_, we derive features such as how many other patients, out of the K most similar to P_i_, have died (defined in Eq. 1), and what is the entropy of death in this same group (i.e. how orderly and regular are those patients with respect to “dying”, defined in Eq. 2). For this estimation, we must first define the similarity metric that compares each patient pair. We define it as a function f(P_i_, P_j_) that can compare two patients, P_i_ and P_j_, with respect to their defining features. For this function, we use the cosine similarities (Eq. 3) of each patient’s feature vector. In order to avoid a quadratic O(n^2^) number of comparisons between patient pairs while defining the top-K most similar ones, we use spatial partitioning with KD-trees. KD-trees are data structures which split the hyperspace into hierarchical orthogonal hyper-planes, with the intention of limiting the number of point comparisons that we need to consider, in order to find any number of closest points. In our experiments, after some preliminary experiments, we chose K = 100 as the size of the neighborhood.

Lethality of the topK most similar patients:

1 $$L(P_{i})= \frac{1}{K} \times \sum \limits_{j=1}^{K}y_{j}$$

Lethality entropy of the topK most similar patients:

2 $$L\left( {P_{i} } \right) = - \sum\limits_{j = 1}^{K} {P\left( {y_{j} } \right) \times \log \left( {P\left( {y_{j} } \right)} \right)}$$

Cosine similarity:

3 $$cos(\theta )=\frac{A \cdot B}{||A|| ||B||}$$

As such, the new proposed meta-features capture general aspects of our sample population, by directly computing populational metrics that gauge the patient with respect to its peers. These new meta-features are inspired in^20^ and have not been applied to death prediction.

**Figure 1:**
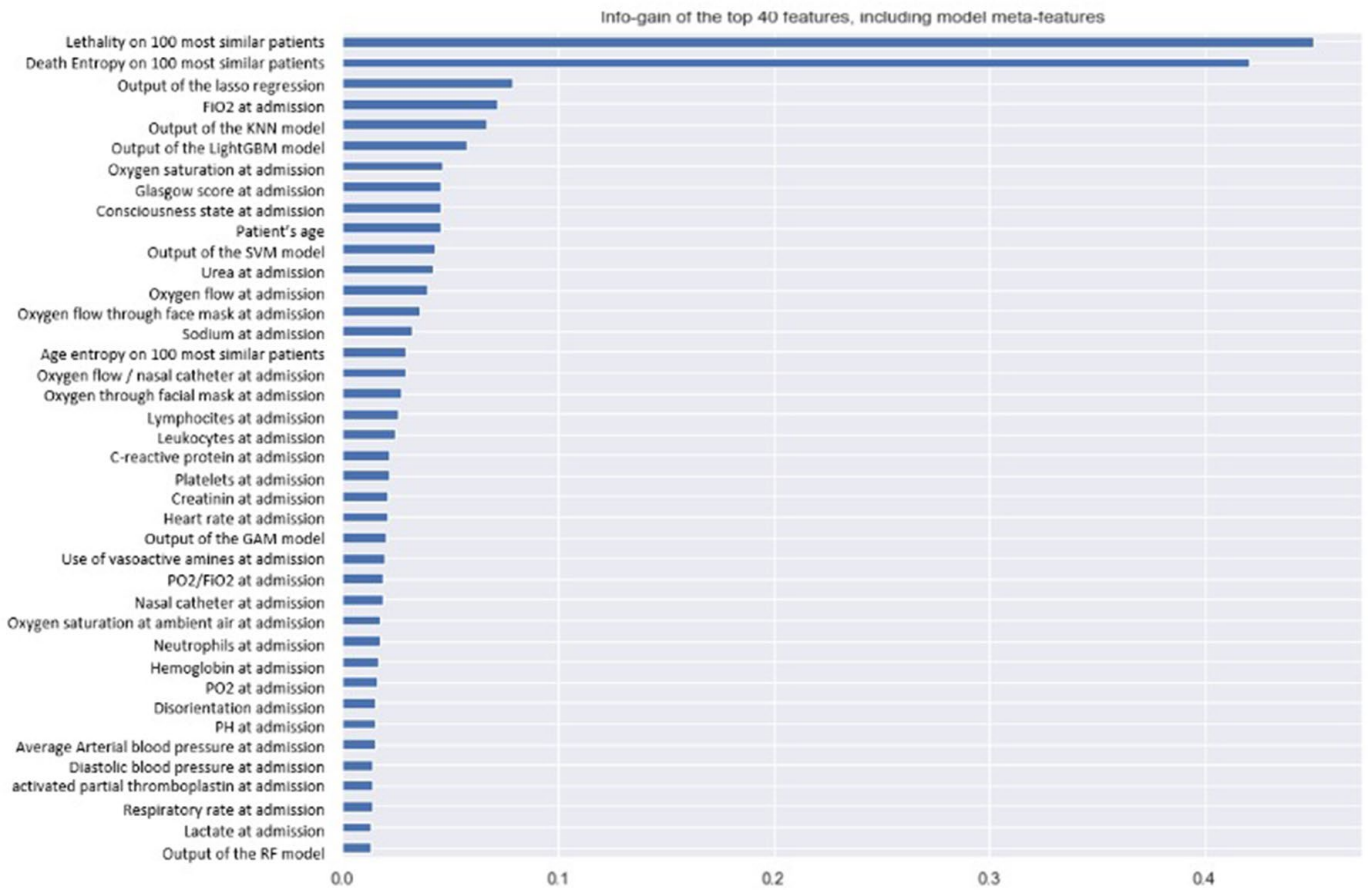
Info-gain on the best model features, including populational and classifier output based meta-features. FiO_2_: fraction of inspired oxygen; GAM: generalized additive models; KNN: K-nearest neighbors; LightGBM: light gradient boosting machines; pO_2_: partial pressure of oxygen; RF: random forest; SVM: support vector machines.

Figure 1 shows the information gain from the top-40 most discriminative features at the Stacking level, including all base (patient), stacking and populational meta-features. The top-3 most predictive features were the meta-features: (i) lethality and (ii) entropy of deaths of the 100 most similar patients; and (iii) output of Lasso regression. Lethality and entropy of deaths of the 100 most similar patients, which are population-based, were the best and runnerup features, with up to four times more discriminative power than the feature that comes in third place in the feature ranking—Output of Lasso Regression—which is also a meta-feature (in this case, a stacking meta-feature). The first base (patient) feature to show up in the rank is “FiO_2_ at admission”, coming in fourth place.

As for the specific machine learning (ML) models compared in our study, we trained three modern neural network benchmarks—the FNet transformer, a deep convolutional Resnet and a deep 1D convolutional neural network. We also experimented with a support vector machine classifier, a boosting model (microsoft research’s Light Gradient Boosting Machine), and the K-nearest neighbors algorithm, as well as a Stacking of these methods and variations with base and population based meta-features as input.

We compare these ML alternatives to traditional statistical methods, including LASSO regression, the current state-of-the-art, and the Generalized Additive Model (GAM). GAM has been recently used in the ABC_2_-SPH score^9^, developed by our group, but only to select variables for the LASSO regression. In the present analysis, we directly tuned GAM to the classification task, thus obtaining better results, as we shall see.

The choice of neural networks to be included in our study was motivated by current state-of-the-art methods, even though, in general, neural networks tend to perform better in situations where massive amounts of data are available, which is not our case, as we have a relatively small data sample^13,21^. Usually, the ability to compare distant input positions in the query vectors is related to the neural network’s depth. Transformer architectures, as introduced by Vaswani^22^, gained rapid success due to their capacity of doing so in a constant number of operations, achieving state-of-the-art results in many tasks. That was the reason we chose a FNet Transformer classifier. For comparison purposes, we also included a Resnet model, which held similar success for image classification, due to the capacity of building very deep networks. Due to the relative drop in the performance of neural networks when fewer data samples are present in training, we also included a training variant, in which we performed virtual adversarial training, as introduced in Miyato^23^. According to the virtual adversarial training the model’s decision boundary is smoothed in the most anisotropic direction through a gradient-based approximation.

Additionally, we included a standard support vector machine classifier, which learns a separation hyperplane between classes, while maximizing the separation margin, and a K-nearest neighbors classifier, which yields predictions based on spatial similarities between training samples and new query points. Motivated by the results shown in Ravid^24^, we included a boosting algorithm (LightGBM), which is usually an effective model in tabular data, as concluded in Ke^25^. As the final classifier, we exploited a meta-learning ensemble-based Stacking model, which learns to combine the prediction outputs of all previous classifiers aimed at improving final classification effectiveness. We compared all these methods to GAM and LASSO regression, the latter being the current state-of-the-art model for this task, as demonstrated in previous work.

We ran all classification experiments using a tenfold cross-validation procedure. This helps to have enough data for training the methods and test in significant portions of the data (which would not be the case if we split the data into completely independent partitions). Moreover, the repetitions provided by this setup allow us to assess the generality of the produced models across different training and test sets as well as to assess the variability of the results across different samples.

For model parameterization, we used the values presented in Table 1. For deep network models, we use an *early stop* to optimize the model, which optimizes the weights until the model has no improvement in the validation set.

**Table 1:** Parameterization of methods.

Methods	Parametrization
SVM	C: [10^–3^, 10^–2^, 10^–1^, 10^0^, 10^1^, 10^2^] Kernel: [linear, rbf, poly, sigmoid] class_weight: [None, 'balanced']
RF	N-estimators: [10, 50, 100, 200, 500, 1000, 2000]
KNN	Neighbors: [2, 4, 6, 8, 16, 32]
LASSO	Alpha: [10^–3^, 10^–2^, 10^–1^, 10^0^, 10^1^, 10^2^]
LIGHT_GBM	N-estimators: [10, 50, 100, 200, 500, 1000, 2000] learning_rate: [10^–3^, 10^–2^, 10^–1^, 30^–1^] colsample_by_tree: [0.5, 1.0]
CNN	Early stop
FNet	Early stop
FNet + VAT	Early stop
ResNet50	Early stop
GAM	No tunning
Stacking	Meta-classifier: logistic regression, alpha: [10^2^]

List of model names: CNN = convolutional neural network, FNet = fourier transformation neural network, FNet + VAT = fourier transformation neural network with virtual adversarial training, GAM = generalized additive models, KNN = K-nearest neighbors, LASSO = lasso regression, LIGHT_GBM = light gradient boosting machines, RF = random forest, SVM = support vector machines, STACKING = a stacking classifier, which combines the outputs of all others. Values in brackets are evaluated in the validation set of the cross validation process.

Finally, we ran all our classifiers with the base (patient) features in isolation or combined with the new population-based meta-features. We also exploited these new population-based meta-features in the Stacking model, which combines the outputs of all other classifiers.

### Experimental setup and evaluation

Multiple imputation with chained equations (MICE) was used to handle missing values on candidate variables (outcomes were not imputed) for all non-tree based algorithms. Mortality outcome was used as a predictor in MICE in the derivation dataset, but not in the validation dataset. The predictive mean matching (PMM) method was used for continuous predictors and polytomous regression for categorical variables (two or more unordered levels). The results of 10 imputed datasets, each with 10 iterations, were combined following Rubin's rules^26^. Tree based models such as Gradient Boosting (LightGBM) and Random Forests can natively handle missing data, and do it under the assumption that data is missing for a reason, as the lack thereof may carry predictive capacity and produce tree splits with positive information gain. For this reason, we do not input data fed into those two algorithms.

In order to properly assess the performance of different models, **six** different metrics were used, including Precision (Eq. 4), Recall (Eq. 5), both micro-average and macro-average F1-score (micro-F1 and macro-F1, Eq. 6), the area (AUROC) under the receiver operating curve (ROC-Curve) for the ‘death’ label, as well as Log Loss (Eq. 7). While common in healthcare-related literature, the AUROC values can be misleading, especially when there is a considerable class imbalance^27^, and even more so when the class of interest is rare (which is usually the case). Therefore, we also included the micro and macro F1 scores as evaluation metrics. The F1 score is the harmonic mean between precision and recall scores, for each class (i.e. one score to estimate how well the model can predict which patients will die, and one to estimate the same regarding which patients will not die). The "average" part, described as either "micro" or "macro", refers to how these results are aggregated. In "macro" averaging, all classes are taken as equally important, while in "micro" averaging, class imbalance is not accounted for in the final result and all individual predictions are considered equally important^28^.

Precision:

4 $$Precision = \frac{Tru{e}_{positives}}{Tru{e}_{positives} + Fals{e}_{positives}}$$

Recall:

5 $$Recall = \frac{Tru{e}_{positives}}{Tru{e}_{positives} + Fals{e}_{negatives}}$$

F1-score:

6 $$\mathrm{F}1 =2 \times \frac{precision \times recall}{precision + recall}$$

Log loss:

7 $$\mathrm{LogLoss }=- \frac{1}{2}\times \sum \limits_{i=1}^{n}\mathrm{y_i } \cdot \mathrm{log}(\mathrm{p}(\mathrm{y_i})) + (1-\mathrm{y_i}) \cdot \mathrm{log}(1-\mathrm{ p}(\mathrm{y_i}))$$

### Ethics approval and consent to participate

The study protocol was approved by the Brazilian National Commission for Research Ethics (CAAE 30350820.5.1001.0008). Individual informed consent was waived due to the severity of the situation and the use of deidentified data, based on medical chart review only. The authors confirm that all methods were carried out in accordance with relevant guidelines and regulations.

## Results and discussion

Classification results for the prediction of death are shown in Table 2. All reported results are the average values of the respective metrics in the ten test folds. The versions of the classifiers that use only the base (patient) features, for the sake of simplicity, have only the name of the classifier. When this input is enhanced with the new population-based meta-features, we made this explicit in the Table.

**Table 2:** Micro-F1, macro-F1 and AUROC results for the prediction of COVID-19 in-hospital death.

	Macro-F1	Micro-F1	Precision-death	Recall-death	Log loss	AUROC
Stacking	0.654	0.821	0.562	0.354	6.032	0.826
LGBM	0.648	0.825	0.555	0.345	6.177	0.824
Lasso + population meta-features	0.633	0.816	0.550	0.319	6.355	0.794
STACKING + population meta-features	0.631	0.809	0.544	0.320	6.593	0.759
GAM	0.630	0.813	0.565	0.309	6.456	0.620
RF + population meta-features	0.626	0.816	0.581	0.299	6.338	0.811
LGBM + population meta-features	0.625	0.812	0.563	0.301	6.504	0.751
CNN1D	0.625	0.776	0.422	0.412	7.721	0.721
SVM + population meta-features	0.619	0.814	0.561	0.281	6.421	0.782
Resnet50	0.617	0.780	0.458	0.381	7.588	0.764
RF	0.617	0.817	0.584	0.275	6.317	0.809
GAM + population meta-features	0.616	0.817	0.580	0.279	6.323	0.609
FNet	0.611	0.779	0.439	0.350	7.642	0.720
SVM	0.608	0.814	0.574	0.255	6.424	0.813
LASSO	0.595	0.809	0.555	0.241	6.611	0.811
Resnet50 + RBF-kernel	0.593	0.752	0.383	0.371	8.577	0.698

List of model names from top to bottom (ordered by MacF1): CNN = convolutional neural network, FNet = fourier transformation neural network, FNet + VAT = fourier transformation neural network with virtual adversarial training, GAM = generalized additive models, KNN = K-nearest neighbors, LASSO = least absolute shrinkage and selection operator regression, LGBM = light gradient boosting machines, RF = random forest, SVM = support vector machines, Resnet50 = Residual Neural Network (with 50 residual blocks), STACKING = a stacking classifier, which combines all others.

As shown in Table 2, the differences among the best methods were somewhat small. It was not possible to perform statistical significance testing because there exists no unbiased estimator for the variance of cross-validation-based performance estimates^29^.

As we can see, Neural network models (CNN—convolutional neural networks, and ResNet—Residual neural network) produced the worst results, while the Stacking, boosting ('LightGBM'—Light Gradient Boosting Machine), GAM with both patient and population-based meta-features produced the best overall results, when considering all the evaluation metrics, especially MacroF1, MicroF1 and AUROC. It is interesting to notice that GAM surpassed the original LASSO model that exploits only the patient features, which was the version used in the ABC_2_-SPH score and was considered the previous state-of-the-art.

The less effective results of the Neural networks are somewhat expected as the size of the dataset is not that huge, with about ten thousand samples. Typically, we expect neural networks of large capacity (millions to billions of parameters) to excel in tasks where very large datasets are available (millions to billions of training instances), which is still very rare in health-related problems (except when the database is extracted from big data sources^30^). In such large scale datasets, neural networks can capture very complex relationships. However, in smaller sample sizes, they show a remarkable tendency to overfitting, hence obtaining poor results in terms of validation error^13,21^.

In general, tree-based ensemble models such as random and boosting forests tend to be more robust to small sample sizes and to overfitting, which is exactly the behavior we observed in our experiments^31^. SVM and K-nearest neighbors (KNN), which are simpler models, with fewer parameters, also tend to perform reasonably well on smaller datasets being better than the neural network models.

We should stress that GAM showed very competitive results for this data sample. Unexpectedly, GAM was even better than the original LASSO (patient features only) and some traditional ML methods such as SVM and KNN. As mentioned in our work, we directly tuned GAM to the classification task, using the cross-validation procedure, which yielded superior performance. The most interesting results in terms of the single classifier models are those obtained with LightGBM (LGBM) using only the patient features, which surpasses all other models.

In any case, the model that consistently produced the highest results for most considered metrics was the **Stacking model,** which is a combination of the output of all other individual models, which, in turn, exploited all the provided base features (Table S2). When considering Micro and Macro-F1, Precision and Recall for death, AUROC and LogLoss (for LogLoss, the smaller, the better). Stacking did not did not loose to any other model in any metric, is the sole winner in terms of LogLoss and ties only with LightGBM in terms of AUROC. Some of the largest gains were in macroF1, with gains of up to 10%, and on Recall to predict death, with more than 46% of improvements, over LASSO, the previous state-of-the-art. In particular, recall for death is an important measure as we do not want to misidentify potential patients that might die if not properly treated.

Indeed, it is possible to observe in Table 2 that the combination of models by means of Stacking yielded improvements over most the best individual single models (SVM, RF, GAM, Lasso, the neural models, etc.), allowing us to better discriminate between patients with higher risk of death at admission presentation. The combination of models based on different classification premises potentially made Stacking more robust. If a single classifier makes a wrong prediction, the others can still make corrections, increasing the robustness of the final stacking model^32–35^.

Finally, with regards to the variations of classifiers that did include our proposed population-based meta-features (+ population-based meta-features), we can see a somewhat ‘dual’ behavior, in which these meta-features greatly improve model effectiveness with less learnable parameters, such as SVM, Lasso regression and GAM, while not really improving (and even worsening) the performance of more complex ones (like Stacking and LightGBM).

In the particular case of Stacking along with population meta-features, despite the high discriminative power of the latter, when both types of features are combined—populational and classifiers outputs—there are some effectiveness losses. This is possibly due to redundancy and loss of generality, as a consequence of increasing the complexity of the model given the higher dimensionality. We will further investigate these issues in future work.

Indeed, the population-based meta-features only yield effectiveness improvements when used along with some weaker classifiers (e.g., LASSO, RF and SVM). Stronger models such as LightGBM can build non-linear relationships among the features that have a discriminative power similar to that of the populational meta-features, while weaker learners benefit more from having single better predictors.

All these results make a strong case that our current Stacking strategies as well as the population-based meta-features push the problem's solution to its current state-of-the-art limits. Classifier errors at this point are mostly due to factors not captured in the input features, as patients that did not die but received a high-risk score were in general more severely ill and were indeed more likely to die. Next, we provide a deeper analysis of the meta-model´s features prediction capability, including the new proposed meta-features and the ML methods´ outputs along with the base features (i.e., vital signs, biomarkers, comorbidities, etc.).

**Figure 2:**
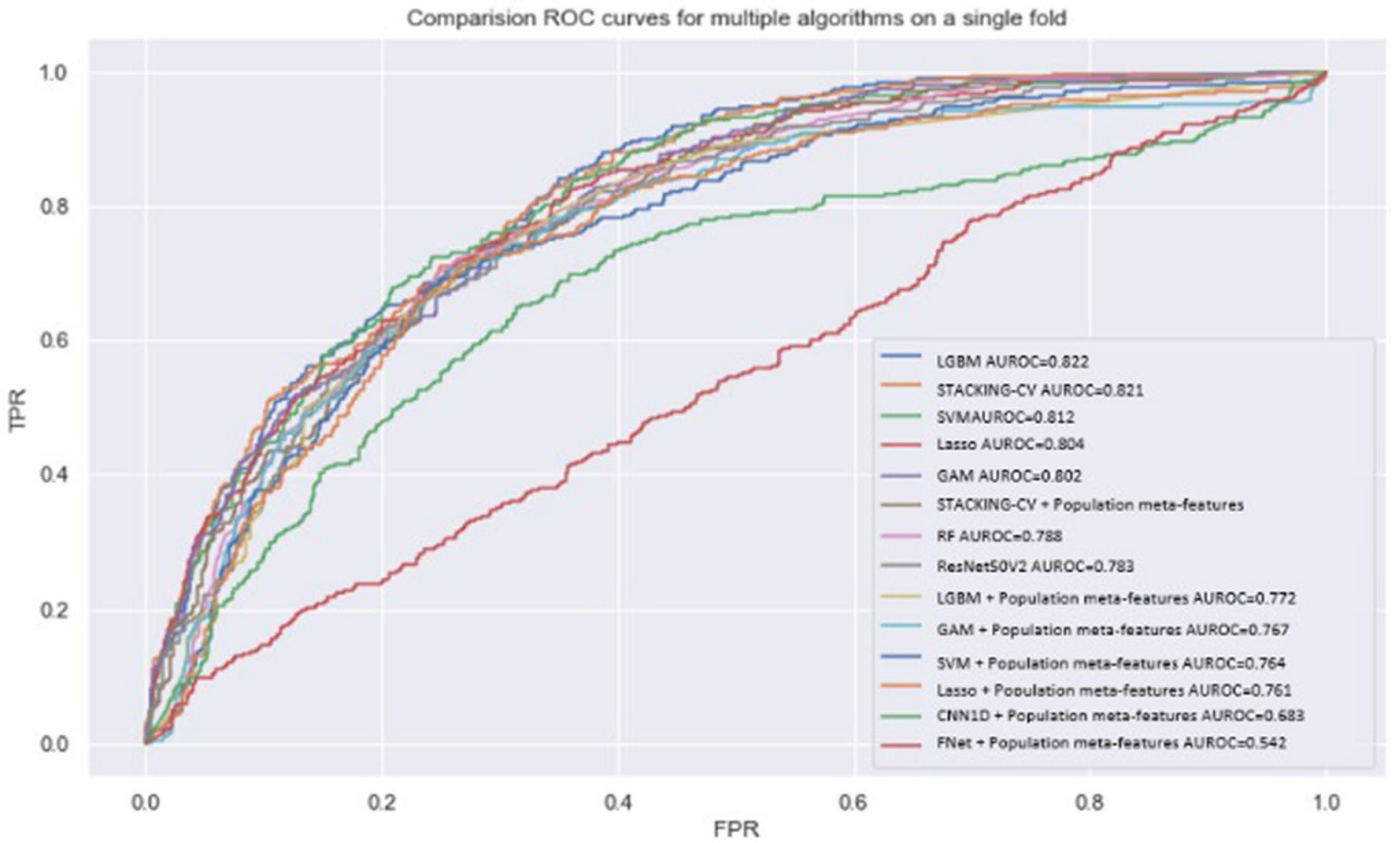
Receiver operating characteristic (ROC) Curve comparing multiple models, trained on the prediction of the death outcome.

As a final analysis, given the popularity of this metric in the health domain, we generated ROC curves for all evaluated models, shown in Fig. 2. In this figure, there is a group of models with inferior results, composed of neural network models and K-nearest neighbors, and a group of models with superior (indistinguishable) results, consisting of SVM, RF, LightGBM, GAM and the Stacking of models. Despite similarities in the curves and at AUROC values, these classifiers may yield quite different results when compared with micro-F1 and macro-F1, or class-specific F1 scores, which shows that (1) AUROC score is not an adequate metric for evaluating and comparing models, especially in face of high imbalance/skewness and that (2) even though some models, like Stacking and SVM have very similar AUROC scores, their capacity to discriminate relevant outcomes like death is quite different (e.g., 0.608 F1 score for SVM and 0.654 for Stacking, a difference of 7.5%).

Another interesting remark is that, using such curves, we can sensibly calibrate the trade-off between sensitivity and specificity, further customizing the way such models can be used. In particular, when applying Stacking, our model can be tailored to the early identification of high-risk patients with good discrimination capacity.

### Dimensionality reduction approaches

It is known that techniques to reduce the dimensionality or the number of variables, such as PCA (*Principal Component Analysis*), RFE (*Recursive Feature Elimination*) and SVD (*Singular Value Decomposition*), can eliminate redundant and irrelevant features. However, these compression methods in many practical situations do not result in classification effectiveness improvements, being more useful for reducing the training cost of models and their complexity. Indeed, PCA, RFE, SVD and other compression alternatives may result in losses in the predictive power of the ML algorithms, as was the case in our tests, shown in Table S3. In those experiments, we applied PCA and SVD transformations to our inputs before training and testing and then proceeded with a tenfold cross-validation procedure, but performance losses are non negligible.

### Explainability of the patients' features

Various prognostic factors have been proposed in the stratification of COVID-19 patients, based on their risk of death, including clinical, laboratory and radiological variables. Among these risk factors, stand out advanced age, multiple comorbidities on admission (such as hypertension, diabetes mellitus, cardiovascular diseases and others), abnormal levels of C-reactive protein (CRP), lymphocytes, neutrophils, D-dimer, blood urea nitrogen (BUN) and lactate dehydrogenase (LDH)^5,7,9,32^.

A very interesting feature of some ML models, in particular decision trees, RF and boosting forests, is the explainability of these models. This is still a very active research area, but modern advances in tools and visualization alternatives allow us to represent which features were most important to the model and at which polarities and intervals. The best model in our tests was the Stacking. However, this is a meta-model, which inputs are the outputs of other classifiers. Because of that, and since we want to explain a classifier that works on the level of the patient features themselves instead of a meta-level of other classifier outputs, in the next feature analysis we use the results of LightGBM, the runner up model. Indeed, tree-based boosting and bagging algorithms rank as some of the most explainable machine learning models, and also lead many benchmarks, particularly for tabular data where data samples are not that large. Their unique combination of explainability, reliability and performance, added to the fact that *Stacking* is a meta-classifier are why we will exploit the boosting model (which, in our case, outperformed the bagging model—Random Forests/RF) to analyze the found correlations among variables.

**Figure 3:**
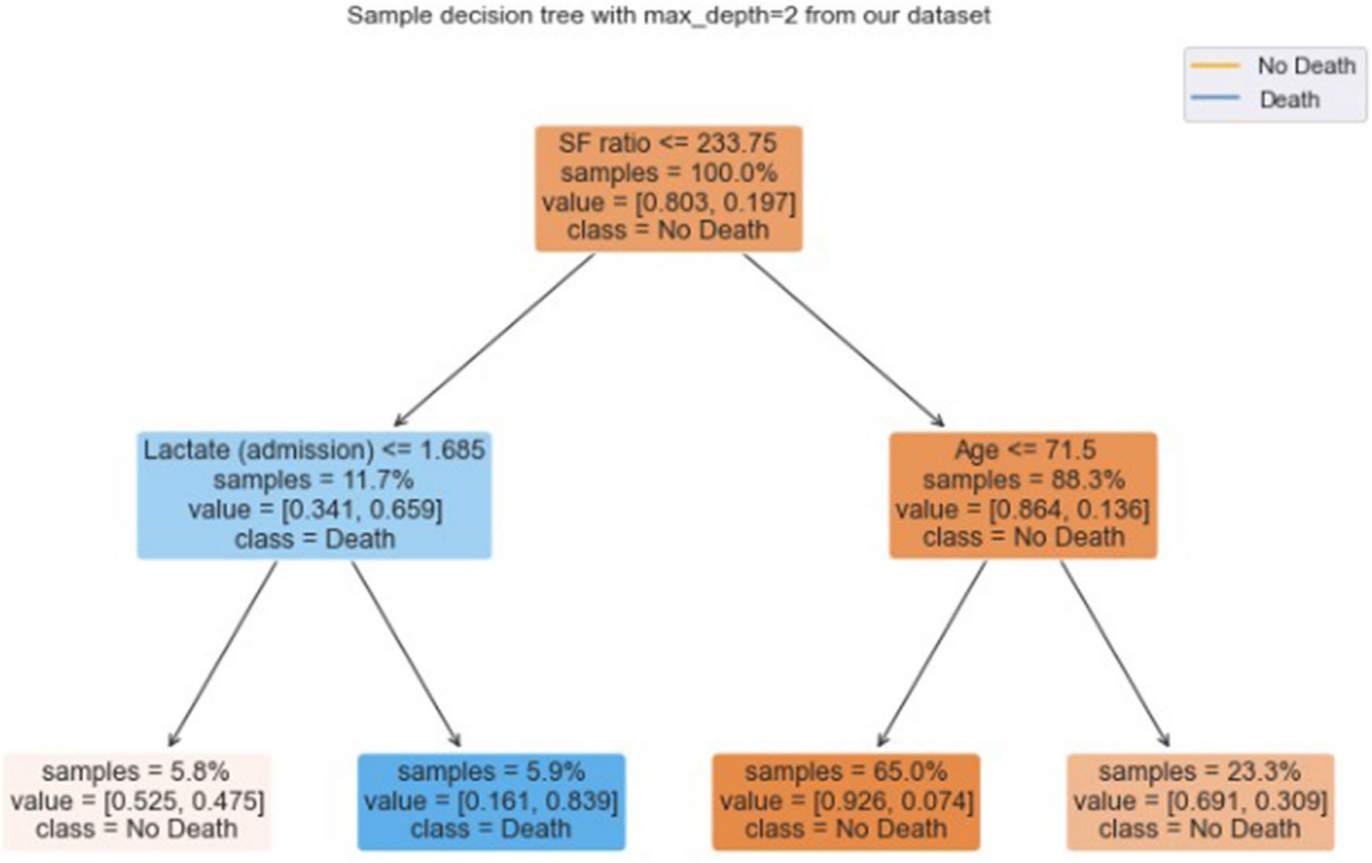
A sample decision tree with depth 2, trained on our dataset. At each level but the last, the first line of text in each box shows the variable and its cut before the split.

In a sense, some traditional models, such as regression models, also have a good explainability, as we can assess the coefficients of each attribute to measure how important a feature is. These models however do not measure up in terms of effectiveness when compared to modern tree-based algorithms in many scenarios, especially in cases with larger datasets^33^. Another key difference between these models is that, in the case of regression models, we have to explicitly remove collinear variables, but these variables, even though they might not improve classification performance, still yield valid model explanations. In addition to that, tree based models can return explanations in the form of intervals, such as the behavior seen in Fig. 3 for sodium and bicarbonate levels, which imply there is a 'safe interval' at which death risk is lower, while either extreme (i.e. too low or too high) has a predictive value for the possibility of a COVID-19 related death.

In decision tree-based algorithms, however, each node represents a feature. The closer to the root (i.e. the 'first' node of each tree), the more the feature is able to differentiate the data classes. For example, in Fig. 3, the feature 'SF ratio'' with a value less than 233 and the feature 'lactate' with a value less than 1.68 mmol/L results in a subset with 5.9% of the dataset where the 'death' outcome is more common.

These algorithms look for the values of the features that further separate the classes, while trying to decrease the coefficient or entropy values of the class label (which are measures of purity and information) in each partition in each decision tree. This coefficient is called the GINI Index. Such index and the entropy score tend to isolate records that represent the most frequent class in a branch.

**Figure 4:**
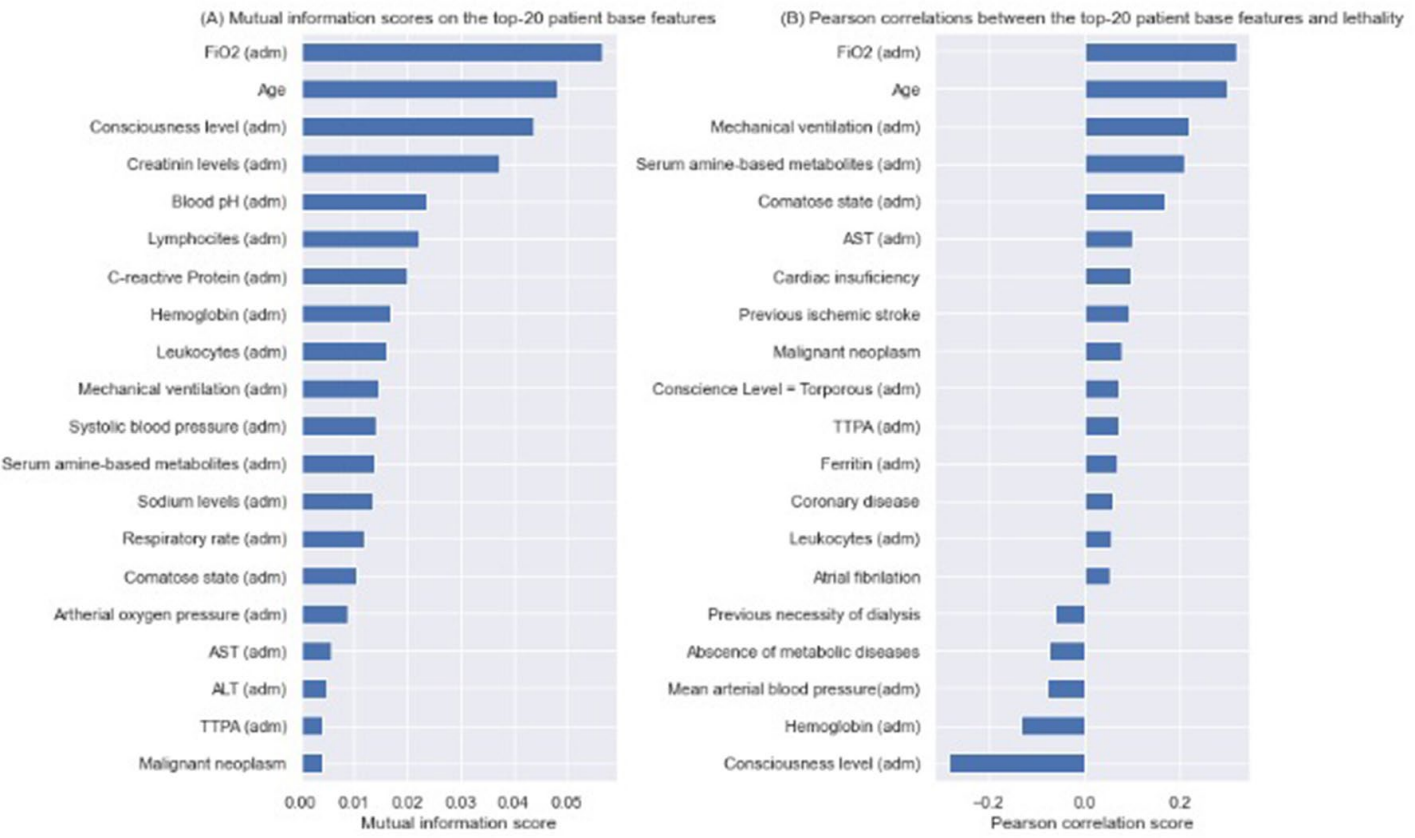
Mutual information scores on the top-20 base patient features (A) and Pearson correlation scores between the top-20 most predictive features and lethality. Adm: admission; ALT: alanine aminotransferase; AST: aspartate transaminase; FiO_2_: fraction of inspired oxygen; TTPA: partial activated thromboplastin time.

Figure 4 shows mutual information (or information gain) score values for the baseline features and Pearson correlation scores between the top-20 most predictive features and inhospital mortality. Information gain allows us to query how much knowing the value of one feature removes uncertainty regarding the distribution of another variable (i.e. in this, case, the outcome variable) ^34^, while Pearson correlations measure how correlated two variables are by comparing their covariance to the geometric mean of their variances. The calculation of mutual information is shown at Eq. (8), where I(X, Y) is the mutual information score, and H denotes Shannon entropy (defined in Eq. 9). The intuition behind mutual information is that, if knowing the value of Y completely removes uncertainty over X, X perfectly predicts Y, and, on the other hand, if no uncertainty is removed, Y is not correlated to X and, therefore, cannot be predicted by it. Additionally, the Equation for Pearson correlation scores is shown in Eq. (10). Pearson correlation scores range from -1 (perfect negative correlation) to + 1 (perfect positive correlation), and a score of 0 indicates no correlation.

Mutual information score:

8 $$I(X, Y) =\mathrm{H}(\mathrm{X}) -\mathrm{ H}(\mathrm{X}|\mathrm{Y})$$

Shannon entropy:

9 $$H(X) = - \sum \limits_{i=1}^{n}p(x_{i}) \times lo{g}_{b}(P(x_{i}))$$

Pearson correlation:

10 $$P(X,Y) = \frac{cov(X, Y)}{\sqrt{var(X) \cdot var(Y)}}$$

With the help of the information gain score and Pearson correlation values in Fig. 4, we can extract interesting knowledge from our features. We can see, for instance, that the most important features in the prediction of death by COVID-19 are age and FiO_2_ required at hospital presentation, and that their respective polarities are that the older the patient is, and the higher the supplemental oxygen flow, the higher the death risk. This is coherent with previous medical literature, and serves as an additional validation to the model. Other scores and a recent meta-analysis have shown age as a key prognostic determinant in COVID-19^36–39^. In a meta-analysis which included more than half million of COVID-19 patients from different countries, the risk increased exponentially after the fifth decade of life^38^. It is important to highlight that this fact could be influenced by both the physiological aging process and the individuals' functional status and reserve, which may hinder the intrinsic capacity to fight against infections, increasing susceptibility to the infection and severe clinical manifestations^40^. On the other hand, the FiO_2_ might imply an additional correlation, as receiving a higher flow of supplementary oxygen correlates to a more severe and more extensive lung infection, and thus being more likely to die. This result is consistent with previous literature, as it is well known that lung involvement is the mainstay for assessing disease severity, and oxygen requirement upon hospital admission has been shown to be an independent predictor for severe COVID-19 in several studies^41,42^.

A recent Brazilian study in a center not included in the present analysis observed that frailty assessed using the Clinical Frailty Scale is a key predictor of COVID-19 prognosis. The authors identified different mortality risks within age and acute morbidity groups. As our study was based on chart review only, we could not assess frailty, but we agree with the study authors that it must not be neglected when assessing COVID-19 prognosis. In addition to helping identify patients with a higher risk of death, it can be valuable in guiding evidence-based discussions on realistic goals patients can achieve^40^.

Consciousness level at hospital presentation was the third best feature according to the information-gain score on predicting mortality. This attribute shares a negative correlation with lethality, meaning that lower scores (i.e. the patient has a less alert consciousness state) are more predictive of lethality. This also makes sense, as consciousness impairment might indicate way more severely ill patients, as the disease had to compromise either brain oxygen flow, blood flow or both.

An interesting remark is that there is no complete overlap between the rankings of information gain and Pearson correlations, except for the very top attributes. Despite measuring similar things, this difference mainly stems from different ranking premises, which in general tend to overlap mainly for the most predictable variables of the target outcome. These two scores measure similar but slightly different things, as both are statistical in nature, but Pearson correlation score measures a normalized covariance between two variables, while the Information-gain measures the drop in entropy (or surprise, novelness of information, etc.) that we get in X by knowing Y.

### Model errors

**Figure 5:**
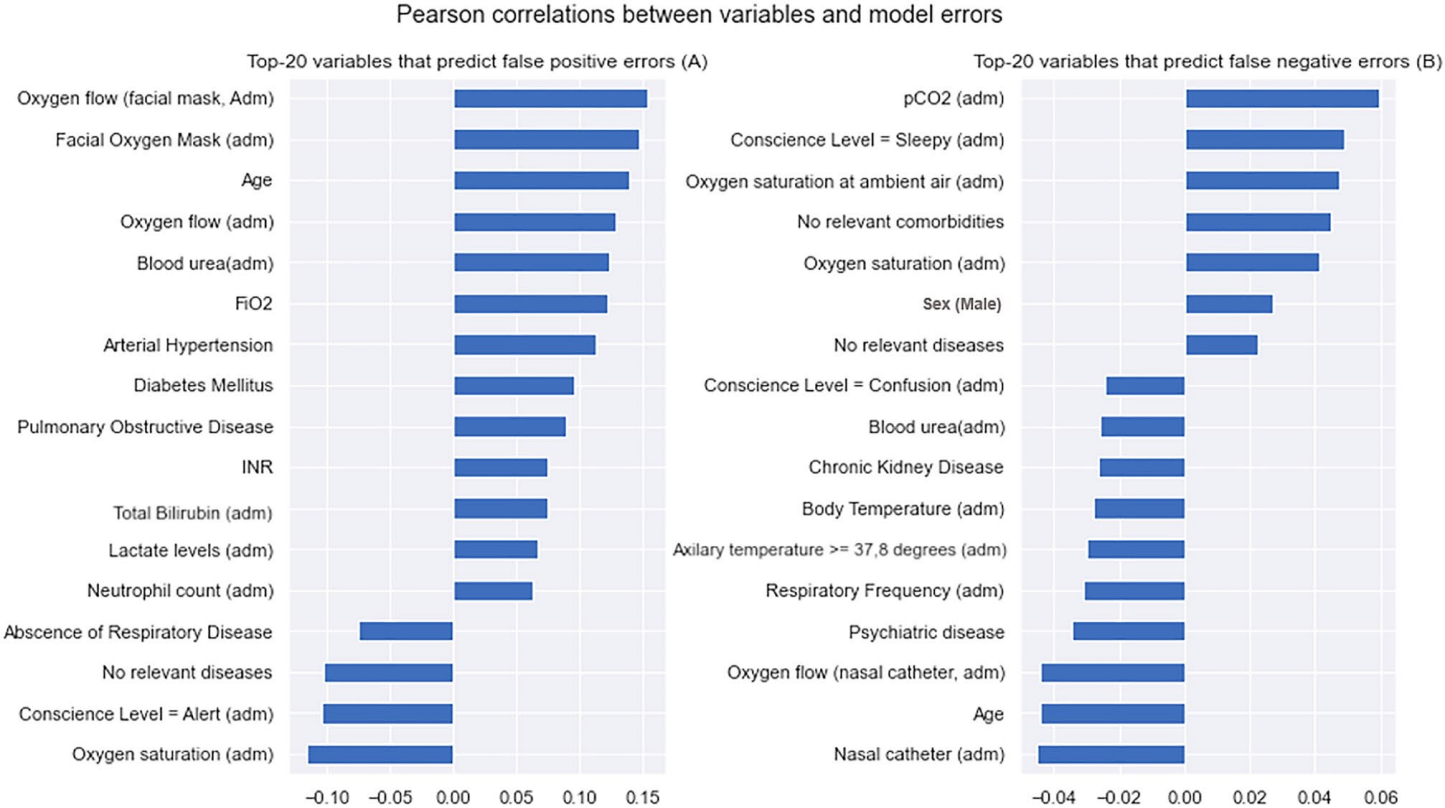
Pearson correlation scores between model variables and false positive/negative errors. Adm: admission; FiO_2_: fraction of inspired oxygen; INR: international normalized ratio; pCO_2_: partial pressure of carbon dioxide.

**Figure 6:**
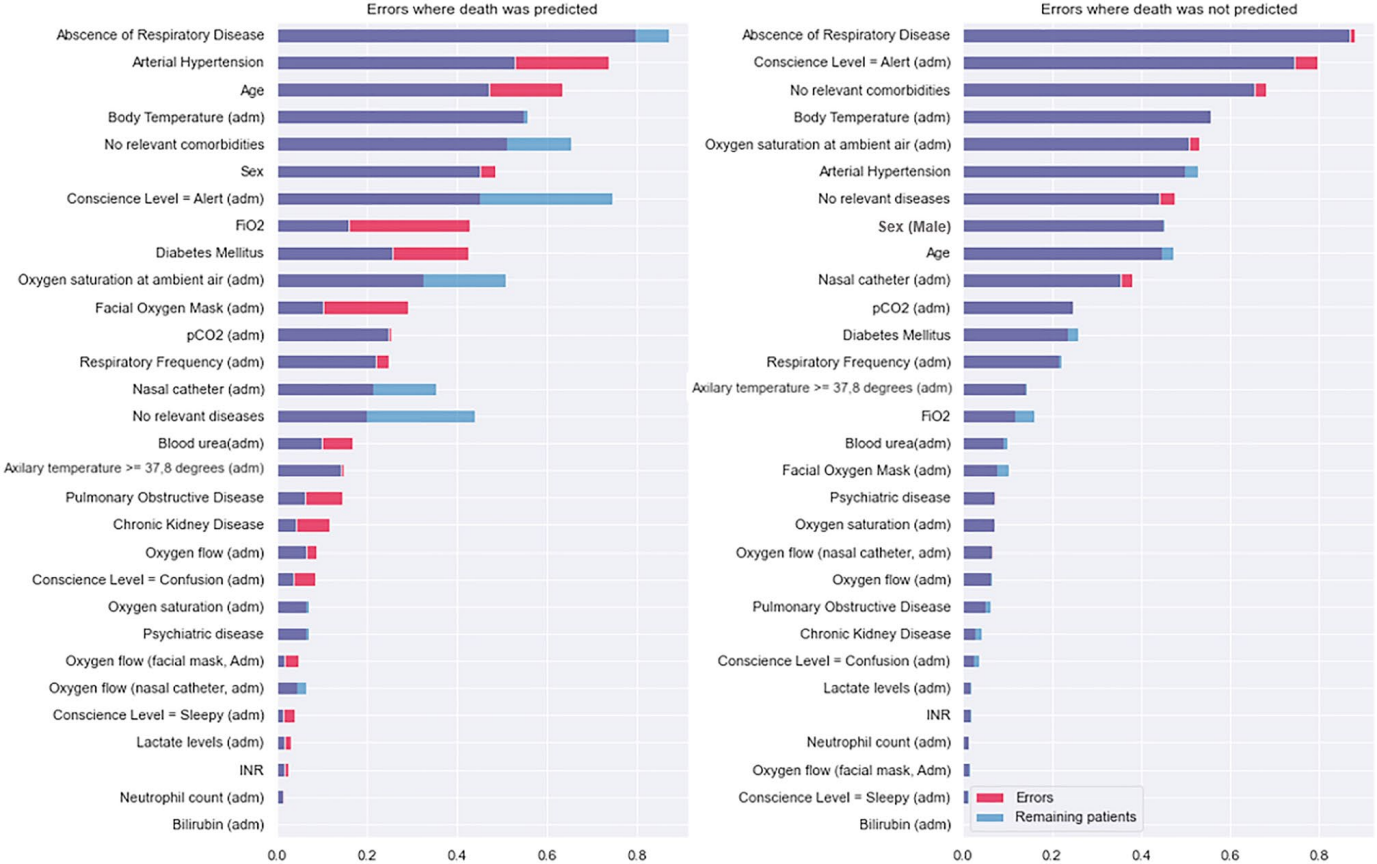
Comparison of mean normalized values between errors and non-errors. Adm: admission; FiO_2_: fraction of inspired oxygen; INR: international normalized ratio; pCO_2_: partial pressure of carbon dioxide.

To better understand the type and quality of produced errors, we dive into the analysis of which base patient features better predict these model errors on our Stacking model, as well as the populational characteristics of such instances (Figs. 5 and 6).

Figure 5 shows Pearson correlation scores when trying to predict **false positive** (i.e. the model predicts a higher death risk but the patient ends up not dying) and **false negative** (i.e. the model predicts a lower death risk but the patient ends up dying) model errors. From Fig. 5A (left), we can see that false positive errors are more likely to occur when patients are admitted while already requiring high supplementary oxygen and have a more advanced age, for instance. Conversely, from Fig. 5B (right), we can see that false negative errors usually occur in the absence of comorbidities and with higher oxygen saturation levels at admission. Some other variables, like “blood urea” levels, for instance, probably are correlated to this type of error because they are also highly effective at explaining a model prediction of “death”, (as seen in the previous section, from the SHAP analysis).

Our analysis is further depicted in Fig. 6, which shows the normalized mean values of variables between the population that was misclassified and the ones that were correctly classified. This figure shows a comparison between two pairs of populations. The ones classified in the high lethality group (correct and incorrect) and the ones classified in the low lethality group (also correct and incorrect). From this igure, we show that, on average, the population misclassified in the high lethality group is more likely to have comorbidities, have a higher mean age, and a lower percentage of individuals with no disease reported. Conversely, the ones in the lower scored death risk are more similar to the general population.

From these comparisons, we can infer that classifier errors might not be due to any factor captured in our variables, as patients classified as “higher risk of death” are, indeed, more likely to have complications for being older and more sickly, while the opposite is also true, since patients that ended up dying from the “lower risk of death” group were very similar to the overall population that did not die.

### Per hospital analysis

**Figure 7:**
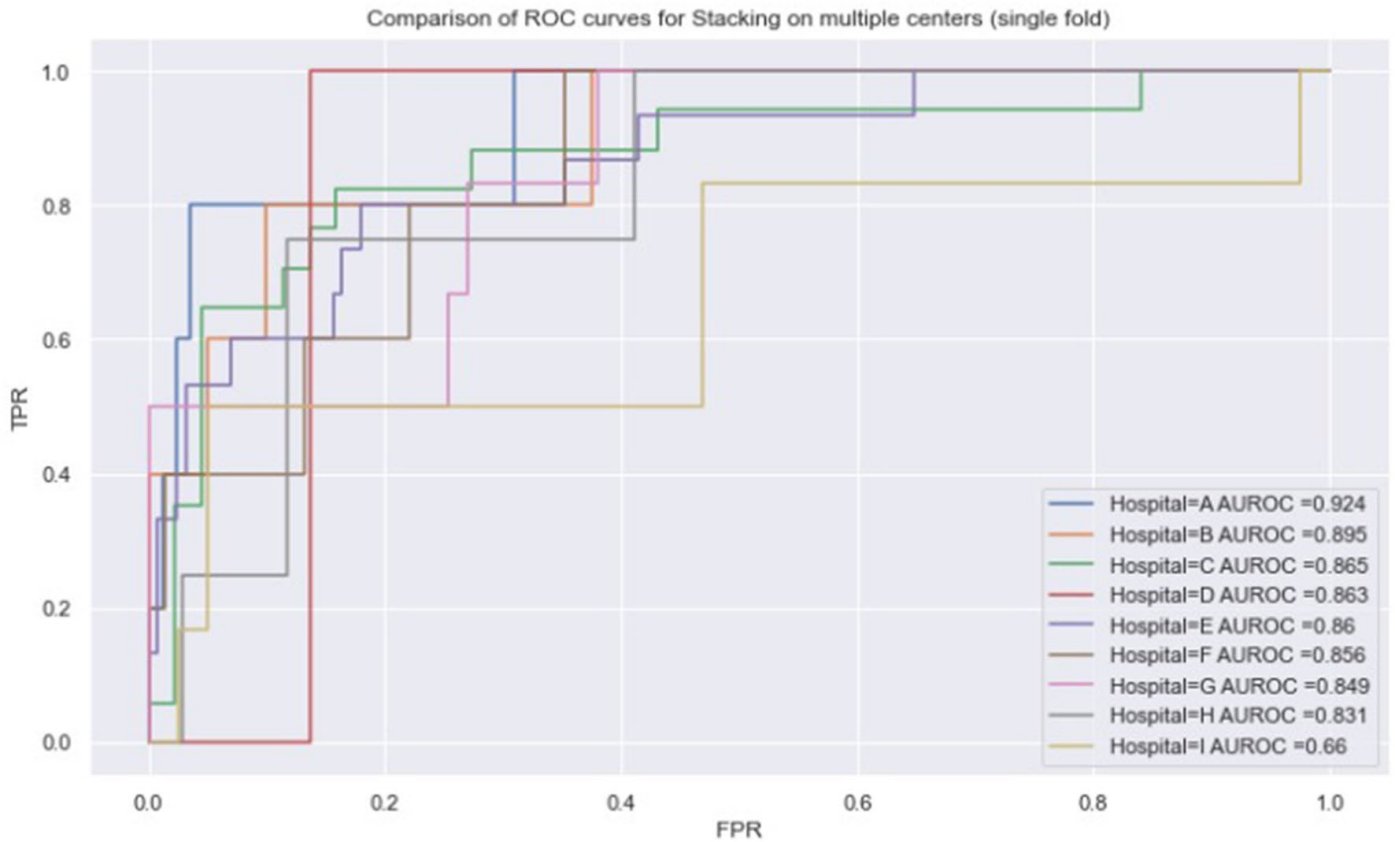
Comparison of ROC curves and AUROC results between different hospitals.

Further expanding our analysis on model errors, we have evaluated how our models´ effectiveness was affected when applied to different centers in the study. Figure 7 shows differences in AUROC between nine of the largest centers in our study, while Table 3 shows differences in F1-score for the death class.

**Table 3:** F1 scores for the death class, per hospital.

Center	A	B	C	D	E	F	G	H	I
Death-F1	0.667	0.5	0.43	0.0	0.476	0.285	0.285	0.0	0.462

Only results of the nine largest centers in our study were included.

These results show large differences in classifier performance among different hospitals. These differences, combined with differences in outcome distribution and posterior probability of death given input attributes of each patient probably explain part of the other classifier errors. Not only more severely ill patients that end up not dying and healthy patients that end up dying generate classifier errors, but also the fact that the decision boundaries themselves are probably slightly different among centers also generates misclassifications. These differences may come from various sources not explicitly captured in our features, as, for instance, different treatment protocols for COVID-19, better or worse material resources—such as mechanical respirators, availability of different drugs, personnel expertise, etc.

**Figure 8:**
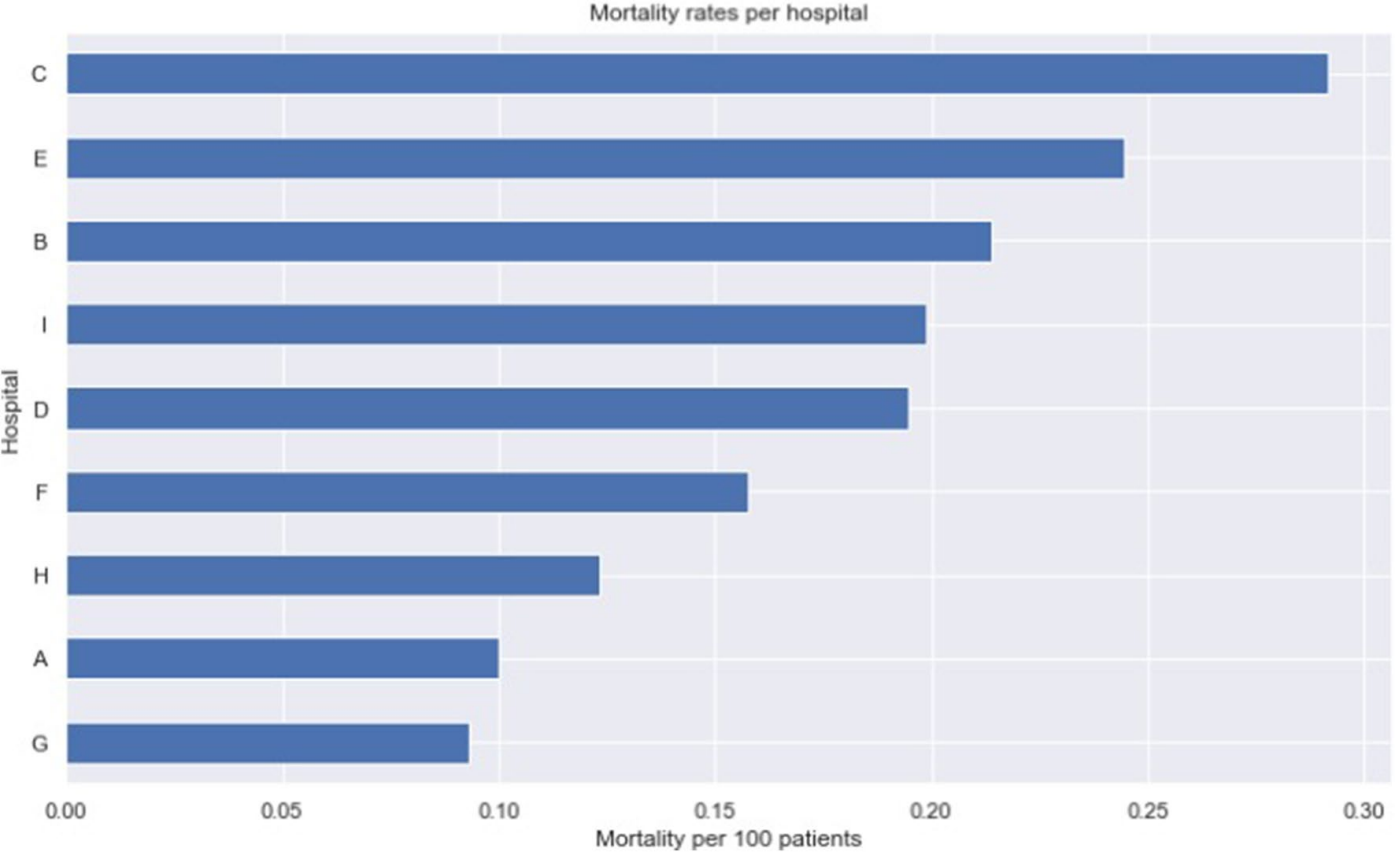
Comparison of ROC curves and AUROC results between different hospitals.

As we can see from a purely distributional perspective in Fig. 8, centers with either much higher and much lower mortality rates tend to have an overall better classifier performance. This is possibly related to easier decision boundaries since overall COVID-19 mortality rates depend on the hospital’s ability to treat and prevent (or not) such deaths, which yields probabilistic odds of survival that are both patient and center dependent. In that sense, a very severely ill patient with near-zero survival odds might be one that would die across multiple centers, thus being easy to classify, while a patient with high death risk, that has a probabilistic chance of not dying that varies in different centers might be harder to classify in one center than another (i.e. imagine his odds of survival are 50% in one center, but 10% in another, and yet 90% in a third one). Models are much more likely to make classification mistakes in the first center than in the latter two, as the outcome in the latter is much more predictable. Other key factors involve how well represented (i.e. higher number of samples) each center has in the training data, and how hard the decision boundary is in that sample.

### Reliability

Finally, we investigated issues related to the reliability of the models. Neural network models are, for instance, known for having irregular error rates, regardless of prediction confidence. At the other end of the spectrum, boosting and bagging models tend to have a very interesting reliability profile, with a tendency to have lower error rates at high confidence scores, and higher error rates at lower confidence scores. This generates a very useful perspective, in which we can tune the trade-off between accuracy and sensitivity for some specific classifiers.

**Figure 9:**
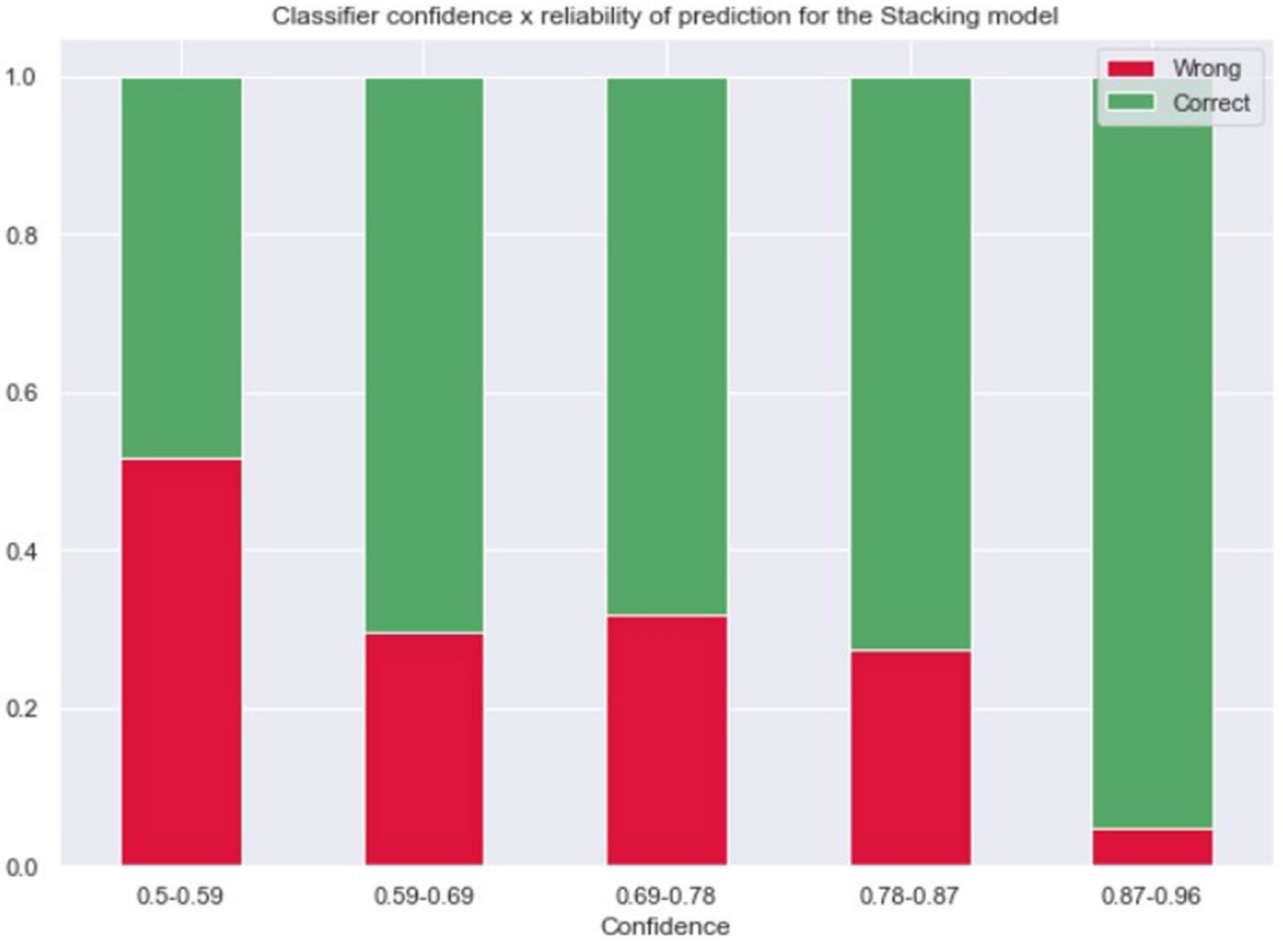
Error rates for each confidence threshold in the Stacking model without populational meta-features (which had the best macro-F1 result). The X-axis shows prediction ranges for the model's confidence score, while the y-axis shows the percentage of hits or misses for the model.

Accordingly, Fig. 9 shows the reliability profile for the best model (Stacking). Note that the model makes more correct predictions (hits, in green) when it is more certain of the prediction (range 0.87–0.96). This classifier yields a useful reliability profile with respect to its confidence score. This kind of characteristic means we can tune how many patients the model will indicate, as well as how sensitive or specific that indication can be. Such tuning can be tailored to any specific healthcare service, accounting for intensive care unit beds, available professionals, and so on.

## Conclusions

In this study, we strive to correct methodological issues and technological limitations of previous studies by experimenting with several modern AI techniques as well as state-of-the-art statistical methods to develop (meta-)models to predict COVID-19 mortality using a large Brazilian multi-hospital dataset containing features available at hospital presentation. We have also devised new population-based meta-features based on distances among similar patients, which have never been exploited in the task of death prediction before. In our experiments, ensemble models excelled in the prediction task, with a meta-learning strategy based on Stacking surpassing the state-of-the-art LASSO regression method by more than 46% for predicting death (Recall), with AUROC of 0.826 and Macro-F1 of 0.654. In particular, Recall for death is an important measure as we do not want to misidentify potential patients that might die if not properly treated. We have also performed an in-depth analysis of our best model´s errors, showing that these errors occur in patients that are indeed harder to classify, and that there is a large variation in classification performance per treatment center. We have empirically shown that mortality is also largely dependent on variables external to the patient, such as which hospital performs the care, possibly due to factors such as differences in therapeutic approach, selection bias (some centers may receive more severely ill patients, for instance), expertise and experience of team members, among others. These factors are hard to quantify and isolate, but seem important in determining future outcomes and therapeutic prioritization, and further research contributions could come from exploring representations and modeling of such factors.

## Supplementary Information

Supplementary Tables.

## Data Availability

Data are available upon reasonable request to the correspondent author.
