# Supplementary material for: Potential and limitations of machine meta-learning (ensemble) methods for predicting COVID-19 mortality in a large inhospital Brazilian dataset
Source: Sci Rep. 2023 Mar 1;13:3463. doi: 10.1038/s41598-023-28579-z (PMC9975879; doi:10.1038/s41598-023-28579-z)
Supplement: Supplementary file 1 — Supplementary Tables. [file 41598_2023_28579_MOESM1_ESM.docx]

**Table S1. Main characteristics of the studies**

| **Study** | **Study design** | **Patient time span** | **Country of derivation** | **Country of validation** | **Sample size (n)** | **Development sample (n)** | **Validation sample (n)** | **Development population** | **Validation population** |
| --- | --- | --- | --- | --- | --- | --- | --- | --- | --- |
| Abbasi et al., | Retrospective cohort study | February 20,  2020 to March  10, 2020 | Not clear | NA | 262 | 262 | NA | Consecutive adult patients (≥ 18 years old) hospitalized with laboratory-confirmed COVID-19  infection | NA |
| Abdollahpour  et al., | Case control | February 18 to  March 26 2020 | Iran | Iran | 513 | 202 | 50 | COVID + with complete data | Same |
| Abdulaal et al., | Retrospective study | February 2,  2020, to April  22, 2020 | United Kingdom | United Kingdom | 398 | 318 | 80 | All patients with real-time reverse transcription polymerase chain reaction (RT-PCR) test–  confirmed SARS-CoV-2 | Same |
| Acar et al., | Cohort | March 16 to  June 18 2020 | Turkey | NA | 709 | 709 | NA | inpatient that received treatment  for COVID-19 disease and to be over age of 18 | NA |
| Adderley el at., | Retrospective cohort | 1 January 2020  to 12 September 2020 (the last admission date was 16 August to  ensure a minimum of 28 days of follow- up). | United Kingdom | United Kingdom | 7139 | 1040 | 6099 | Setting Model development was performed in data from University Hospitals Birmingham (UHB). Patients of all ages diagnosed with COVID- 19 and hospitalised were included | External validation was performed in the  CovidCollab dataset, and also external validation of ISARIC 4C score in UHB data |
| Agarwal et al., | Retrospective  observational study | Not clear | India | NA | 203 | 203 | NA | Hospitalized COVID-19 patients | NA |
| Ageno et al., | Cohort | February 17 to  May 8 2020 | Italy | Italy | 610 | 335 | 275 | Patients with a diagnosis of COVID-19 aged 18 years or older admitted to the Emergency Department or to a Medical Ward directly from  the Emergency Department. | Patients from Verona and Modena |

| Akdur et al., | Retrospective cohort study | July to November 2020 | Turkey | Turkey | 655 | NA | NA | [1] Age ≥ 18 years; [2] SARS-  CoV-2 in respiratory tract specimens, detected by RT-PCR;  [3] high resolution computed tomography (HRCT) performed;  and [4] no missing medical records. | Same |
| --- | --- | --- | --- | --- | --- | --- | --- | --- | --- |
| Aliberti et al., | Cohort study | 30 March to 30  June 2020 | Brazil | NA | 2,078 | 2,078 | NA | All patients aged ≥50 years consecutively admitted to the hospital between 30 March and 30 June 2020. We included those with confirmed (detection of the new coronavirus using reverse transcription-polymerase chain reactions) cases of SARS-CoV-2  infection. | NA |
| Allenbach et al., | Prospective single-center observational cohort study | March 16th 2020 till the 4th of April | France | France | 152 | 152 | Internal validation: 152  External validation: 132 | Older than 18 years with initial requirement for hospitalization in medical ward, and diagnosed with COVID-19, defined as positive SARS-CoV-2 real-time reverse transcriptase–polymerase  chain reaction (RT-PCR) assay from nasal swabs. | Same |
| Altini et al., | Retrospective study | 14 March to 10 September 2020 | Italy | Italy | 303 | 303 | Not clear (k- fold cross- validation) | Patients diagnosed with COVID- 19 who were admitted  to the Polyclinic Hospital of Bari during the first phase of the  COVID-19 global pandemic | Same |
| Altschul et al., | Retrospective single-center cohort | March 1, 2020  to April 16,  2020 | United States | United States | 4711 | 2355 | 2356 | Patients with confirmed COVID- 19 from an academic hospital | The same as the development population (spitted 50/50%, apparently by admission  date) |

| Avendaño‐ Ortiz et al., | Not clear | Not clear | Spain | Not clear | 235 | 69 | 166 | Patients and 15 healthy volunteers  (HVs) from the emergency department (ED) of La Paz University Hospital | Prospective validation independent cohort of COVID-19 patients |
| --- | --- | --- | --- | --- | --- | --- | --- | --- | --- |
| Aznar-Gimeno et al., | retrospective cohort study | February 2020 to January 2021 | Spain | Spain | 3623 | 75% | 25% (internal validation) + 676 (external temporal validation) | patients with SARS-CoV-2 confirmed by RT-PCR who were hospitalized within the first 20 days after, and no more than 10  days before, the first positive SARS-CoV-2 PCR test. | Same |
| Bai et al., | Retrospective study | January 28,  2020, to  September 14, 2020 | China | Gemany | 1433 | 1352 | 81 | COVID-19 patients from January 28, 2020, to March 29, 2020, at Wuhan Union Hospital (also called Wuhan cohort), China, for model development. | Patients with confirmed COVID-19 disease were collected from the University Hospital of Würzburg (also called Würzburg cohort), Germany, from March 6, 2020, to September 14,  2020 |
| Banoei et al., | Retrospective | June 2020 | United States | United States | 250 | 172 | 78 | Patients with a polymerase chain reaction (PCR) test confirmed patients with COVID-19 admitted at the University of Miami Hospital, Miller School of Medicine, Miami, FL, United  States, since June 2020. | Same |
| Baqui et al., | Retospective observational study | February 25 to  September 21,  2020 | Brazil | Brazil | 231,112 | 184,889 | 46,223 | Patients hospitalized patients that had a positive RT-PCR test for SARS-CoV-2, that contain  geographic information and type of healthcare | Same (internal validation) |
| Bartoszko et al., | Retrospective cohort study | 1 March 2020  to 15 December 2020 | Canada | Canada | 127 | 127 | Not clear (internal validation) | Adult patients (C 18 yr) with polymerase chain reaction-  confirmed SARS-CoV2 infection (Seegene AllplexTM | Same |

|  |  |  |  |  |  |  |  | 2019 n-CoV assay; Seegene Inc., Seoul, South Korea) receiving invasive mechanical ventilation at any  point during their ICU stay were eligible for inclusion. |  |
| --- | --- | --- | --- | --- | --- | --- | --- | --- | --- |
| Bello-Chavolla et al., | Registry data from an open source database from the Mexican Ministry of  Health | First patient up to May 18,  2020 | Mexico | Mexico | 51633 | 41307 | 10326 | Patients with confirmed COVID- 19 from the open source Mexican Ministry of Health database (inpatients and outpatients) | The same as the development population (split by random sampling stratified by mortality status) |
| Bello-Chavolla et al., | Retrospective cohort | March 16th to August 17th, 2020 | Mexico | Mexico | 3007 | 1831 | 1176 | Patients aged >18 years with complete clinical data and had confirmed SARS-CoV-2 infection by RT-PCR test in  respiratory samples | Same |
| Bennouar et al., | Cohort, prospective, single-center study | July the 6th and August the 15th, 2020 | Algeria | NA | 120 | 120 | NA | Adult confirmed COVID-19 severe and critical form, and who had been tested on admission for vitamin D and serum calcium levels were included in this study. | NA |
| Bennouar et al., | Cohort | March 27 april  22 2020 | Algeria | Algeria | 576 | 329 | 247 | COVID + / suspected patients | Same |
| Berenguer et al., | Cohort | February 2 to  March 17,  2020 | Spain | Spain | 6161 | 4035 | 2126 | Patients with COVID-19 admitted to 127  hospitals distributed across all regions in Spain | Patients from COVID- 19@HULP in  Spain |
| Berry et al., | Retrospective  ,  observational, | March 1, 2020  and April 22,  2020 | United States | United States | 3123 | 1561 | 1562 | 1) Positive SARS-CoV-2 diagnosis by reverse-  transcriptase polymerase chain | Same |

|  | multicenter cohort study |  |  |  |  |  |  | reaction and 2) Hospitalization at one of HMH’s 13-hospitals within the time frame of March 1, 2020 until April 22, 2020. For the purposes of generating the mortality-risk model we also excluded 1) pregnancy, 2) enrollment in a randomized clinical trial, and 3) died on the  day of admission to the hospital. |  |
| --- | --- | --- | --- | --- | --- | --- | --- | --- | --- |
| Bertsimas et al., | International multicenter retrospective cohort | February to May 2020 | Italy, Spain, United States | Spain, Greece, United States | 3927 | 3062 | 865 | Adult patients who were admitted to the hospital with confirmed SARS-CoV-2 infection by polymerase chain  reaction testing of nasopharyngeal samples | Same |
| Boudou et al., | Retrospective cohort study | 29th February and 30th November, 2020 | Republic of Ireland | Republic of Ireland | 47,265  (cases were randomly partitioned into model training [80%] and validation [20%]  subsets) | 37,812 | 9,453 | Patients with laboratory- confirmed symptomatic COVID- 19 infection included data extracted from national COVID- 19 surveillance forms (i.e., age, gender, underlying health conditions, occupation) and geographically-referenced potential predictors (i.e.,  urban/rural classification, socio- economic profile). | Same (internal validation) |
| Brieghel et al., | Retrospective Cohort | March 2 to  June 2 2020 | Denmark | NA | 263 | 263 | NA | Adult patients with laboratory- confirmed COVID-19 admitted to Herlev and Gentofte Hospital for at least 24 hours between 2 March and 2 June 2020 | NA |
| Cai et al., | Retrospective cohort study | December 30,  2019, and  April 17, 2020 | China | China | 12862 | 6431 | 6431 | Adult hospitalized patients with laboratory confirmed COVID-19 | Same |

| Castro VM, McCoy TH, Perlis RH | Prognostic study | June 7, 2020,  to January 22,  2021 | United States | No | 2892 | 2892 | No | Individuals aged 18 years or older who were hospitalized at 1 of 2 academic medicais centers and 4 community hospitals, with a positive polymerase chain reaction test for SARS-CoV-2  within 5 days of admission | No |
| --- | --- | --- | --- | --- | --- | --- | --- | --- | --- |
| Chen et al., | Retrospective cohort | The first patient to January 31,  2020 | China | China | 1590 | 1590 | NA | Patients with confirmed COVID- 19 from 575 hospitals throughout China, excluding cases with incomplete medical records  (20.8%) | NA |
| Cheng et al., | Retrospective single-centre study | Between 8  February 2020  and 11 March  2020 | China | China | 305 | 305 | 305 | The diagnosis and clinical types of COVID-19 were classified according to the clinical guidelines (version 5 trial) developed by the National Health Committee of the People's Republic of China [(ht](http://www.nhc.gov.cn/))t[p://www.nhc.gov.cn/).](http://www.nhc.gov.cn/)) The diagnostic criteria were as follow: clinical diagnosis criteria of (i) fever or respiratory symptoms and (ii) leukopenia or lymphopenia; and (iii) computed tomography (CT) scan showing radiographic abnormalities in the lung. Patients with at least two clinical diagnostic criteria and a positive result for high- throughput sequencing or real-  time PCR assay were diagnosed as COVID-19-positive. | Same |
| Cho et al., | Cohort | January to  April 2020 | South korea | South  Korena | 5594 | 3729 | 1865 | PCR + COVID inpatients | Same |

| Chow et al., | Single-center retrospective observational cohort study | Derivation: March 1, 2020  to April 31,  2020  External validation: March 12,  2020 to April  7, 2020 | United States | United States | 3248 | 3208 | 40 | Sequential patients with COVID- 19 disease diagnosed by nucleic acid detection from nasopharyngeal or throat swabs at the University of California, Irvine Medical Center (UCI Health) | Patients in the validation cohort were randomly selected from a radiology database of patients who underwent imaging with a clinical concern for COVID-19 disease from March 12, 2020 to April  7, 2020 and were diagnosed with COVID- 19 by nucleic acid detection from  nasopharyngeal swabs |
| --- | --- | --- | --- | --- | --- | --- | --- | --- | --- |
| Chua et al., | Cohort | March 1 to  May 16 2020 | England | England | 983 | 770 | 290 | All individuals aged 18 or older who tested positive for SARS- CoV-2 nucleic acid by real-time reverse transcriptase PCR between 1 March and 16 May 2020 after presenting to the emergency department (ED) at Watford Hospital, West Hertfordshire NHS Hospitals Trust | A smaller population of SARS-CoV-2-positive  cases from Aintree Hospital, Liverpool |
| Chung et al., | Nationwide cohort | April 2020 | South Korea | South Korea | 5601 | 4480 | 1121 | COVID-19–confirmed patients obtained through the Korea  Disease Control and Prevention Agency (KDCA) in South Korea | Same |

| Chuperk et al., | Cohort | March 4  trought June 29  2020 | United States | United States | 4019 | 4019 | Not clear | Consecutive adults (age > 18 yr) admitted to the ICU with  laboratory confirmed COVID-19 admitted between  March 4 and June 29, 2020 | Same (internal validation) |
| --- | --- | --- | --- | --- | --- | --- | --- | --- | --- |
| Clift et al., | Population based cohort study | The derivation and first validation cohort period was 24 January  2020 to 30  April 2020. The second temporal validation cohort covered the period 1  May 2020 to  30 June 2020 | England | England | Not clear | 6.08 million | 2.17 million | People aged 19-100 years registered with participating general practices in England | Same |
| Corradi et al., | Cohort | March 5 to  March 30,  2020 | Italy | NA | 77 | 77 | NA | Patients with laboratory- confirmed COVID-19 infection admitted to our intermediate care unit | NA |
| Covino et al., | Prospective observational study | September 1 to  November 30  2020 | Italy | NA | 239 | 239 | NA | All the patients ≥ 80 years, consecutively admitted to  our ED with confirmed COVID- 19 over a three-months period (September 1st to November  30th, 2020). | NA |

| Dashti et al., | Cohort | Not clear | United States | United States | 12347 | 12347 | 1851 | Patients tested positive for COVID-19 at MGB facilities | Same |
| --- | --- | --- | --- | --- | --- | --- | --- | --- | --- |
| Deng et al., | Retrospective study | 30 January to  30 March 2020 | China | NA | 100 | 100 | NA | All hospitalized patients with positive SARS-CoV-2 nucleic acid through pharyngeal swab test by using the reverse transcription-polymerase chain reaction (RT-PCR) assay | NA |
| Di Castelnuovo et al., | Retrospective observational study | February 19th to May 23rd, 2020 | Italy | NA | 3971 | 3971 | NA | Hospitalised adult (≥18 years of age) patients who all had a positive test result for the SARS- CoV-2 virus at any time during their hospitalisation | NA |
| Ding et al., | Retrospective cohort study | January 18 to  April 25, 2020 | China | NA | 2073 | 2073 | NA | Hospitalized patients laboratory- confirmed patients with symptoms of fever, or respiratory symptoms such as cough or dyspnea, showing the radiologic features of viral pneumonia. | NA |
| Doganci et al. | Retrospective cohort study | 23 March to 18  May 2020 | Turkey | NA | 798 | 798 | NA | Confirmed COVID-19 cases from 23 March to 18 May, 2020 were included  consecutively | NA |
| Doher et al., | Retrospective cohort study | March 04 and  May 13, 2020 | Brazil | NA | 201 | 201 | NA | We included adult patients (≥18 years old) with confirmed severe acute respiratory syndrome  coronavirus 2 infection without CKD or dialysis | NA |

| Dominguez- Olmedo et al., | Retrospective study | March and June 2020 | Spain | Spain | 1823 | 1823 | Not clear | Hospitalized patients with confirmed COVID-19 | Same |
| --- | --- | --- | --- | --- | --- | --- | --- | --- | --- |
| Ebrahimi et al., | Retrospective study | 10 June to 26 December 2020 | Iran | Iran | 283 | 283 | Not clear | Patients with the age of ≥18 years who had previously recovered from COVID-19 disease but were re-infected. | Same (internal validation) |
| El-Raheem et al., | Retrospective cohort study | November- December, 2020 | Sudan | NA | 105 | 105 | NA | COVID-19 patients attended in Hospital | NA |
| Eskandar et al., | Retrospective study | March 1 and  April 16, 2020 | United States | NA | 581 | 581 | NA | All patients with real-time reverse transcriptase PCR– positive assay testing for SARS- CoV-2 RNA were included. | NA |
| Faisal et al., | Registry data | March 11,  2020 to June  13, 2020 | United Kingdom | United Kingdom | 6444 | 3924 | 2520 | Consecutive adult non-elective or emergency medical admissions (COVID-19 and non- COVID-19 patients) from one hospital, who were discharged over a course of three months and had electronic NEWS2 recorded | Consecutive adult non- elective or emergency medical admissions (COVID-19 and non- COVID-19 patients) from another hospital, who were discharged over a course of three months and had electronic  NEWS2 recorded |

| Fan et al., | Retrospective observational study | Not disclosed | China | Iran | 139 | 96 | 43 | Adult patients with confirmed SARS-CoV-2 infection in China | Adult patients with confirmed SARS-CoV-2 infection in Iran |
| --- | --- | --- | --- | --- | --- | --- | --- | --- | --- |
| Fernandes et al., | Cohort | March 1, 2020,  and 28 June,  2020 | Brazil | Brazil | 1040 | 728 (70%) | 312 (30%) | Hospitalized patients with a RT- PCR diagnostic exam for COVID-19 from a large hospital chain in the city of São Paulo | Same |
| Fumagalli et al., | Retrospective cohort | 22 February  2020 to 10  April 2020 | Italy | NA | 516 | 516 | NA | Consecutive patients≥18 years admitted for COVID-19 | NA |
| Galiero et al., | Retrospective observational cohort study | March-June 2020 | Italy | NA | 618 | 618 | NA | All adult patients (≥ 18 years) with laboratory confirmed SARS-CoV-2 infection, who completed their hospitalization (discharged or dead) in the period | NA |
| Galloway et al., | Retrospective cohort | March 24,  2020 to April  17, 2020 | England | NA | 1157 | 1157 | NA | Patients with confirmed COVID- 19 from 2 academic hospitals | NA |
| Garrafa et al., | Retrospective cohort | March 2020 and December 2020 | Italy | Italy | 2782 | 1474 | 632 | COVID-19 patients admitted to the ED and hospitalized from the first wave (March-April 2020) | COVID-19 patients admitted to the ED and hospitalized from the first wave (March-April 2020 |

| Gatti et al., | Retrospective study | March 9 April  10 2020 | Italy | NA | 346 | 346 | NA | Patients admitted to the emergency department of two large North-Western Italy hospitals between March 9 and April 10, 2020, at the peak of the local Covid-19 pandemic outburs | NA |
| --- | --- | --- | --- | --- | --- | --- | --- | --- | --- |
| Giradin et al., | Observational study | March 2 to  May 24 2020 | United States | NA | 4446 | 4446 | NA | PCR + COVID inpatients | NA |
| Goméz et al., | Retrospective single-center cohort | February 24,  2020 to March  16, 2020 | Spain | NA | 163 | 163 | NA | Adult patients with suspected COVID-19 admitted to one university hospital | NA |
| Gopalan et al., | Retrospective study | 1st May 2020 and 30th November 2020 | India | No | 746 | 746 | Not clear | De-identified data from case records of COVID-19 confirmed patients (by real-time Reverse Transcription Polymerase Chain Reaction tests obtained from nasal or oropharyngeal swabs), hospitalized to a public sector COVID-19 tertiary care centre | Same (internal validation) |
| Gorham et al., | Retrospective study | March 10 (i.e. the date of the first admitted patients) and April 30, 2020 | Belgium | NA | 41 | 41 | NA | Adult patients with COVID-19 by RT-PCR admitted to the ICU with repeated measures os IL-6 | NA |

| Guan et al., | Retrospective cohort study | 27 January and  5 April, 2020 | China | China | 1270 | 554 | 233 (internal)  286 (external) | Hospitalized laboatory- confirmed COVID-19 patients | Same |
| --- | --- | --- | --- | --- | --- | --- | --- | --- | --- |
| Gude- Sampedro et al., | Retrospective cohort study | 6 March 2020  to 7 May 2020 | Spain | Spain | 2492 | 1745 | 747 | Hospitalized patients diagnosed with COVID-19 | Same |
| Gue et al., | Retrospective single-center cohort | March 10,  2020 to May  30, 2020 | United Kingdom | NA | 316 | 316 | NA | Consecutive patients with confirmed COVID-19 from a general hospital, who had clinical symptoms at admission | NA |
| Gupta et al., | Retrospective cohort study | February 6 and  August 26,  2020 | United Kingdom | United Kingdom | 74 944 | 66 705 | 8239 | Consecutive adults (aged ≥18 years) who had highly suspected or PCR-confirmed COVID-19 | Same |

| Gupta et al., | Observational cohort | Derivation: March 1, 2020,  and April 30,  2020;  Validation: June 1, 2020,  and December 31, 2020 | United States | United States | 3739 | 1672 (but in certain places of study they cite 1673) | 2067 | All patients older than 18 years admitted to Ochsner Health system hospitals with COVID-19 infection throughout Louisiana from March 1, 2020, through  April 30, 2020 | All hospitalized patients with  COVID-19 infection from June 1, 2020,  through December 31,  2020 |
| --- | --- | --- | --- | --- | --- | --- | --- | --- | --- |
| Hajifathalian et al., | Retrospective cohort | March 4, 2020  to April 9,  2020 | United States | United States | 929 | 664 | 265 | Adult patients with confirmed COVID-19 patients presenting to emergency department of 2 hospitals in Manhattan (did not exclude patients who were discharged within 24 hours) | Adult patients with confirmed COVID-19 patients presenting to emergency department of 9 hospitals in Massachusetts (did not exclude patients who were discharged within 24 hours) |
| Halalau et al., | Retrospective cohort | March 1, 2020  to April 1,  2020 | United States | United States | 2025 | Not clear | 1290 | Not clear | Confirmed SARS-CoV-2 patients who required hospital admission at 8 hospitals in Beamount, excluding patients who remained hospitalized beyond May 12, 2020 |
| He et al., | Retrospective cohort study | January 18,  2020, to March  24, 2020 | China | China | 1114 | 1114 | NA | Patients with a positive nucleic acid test for SARS-CoV-2 were collected from the COVID-19 designated hospitals in Wuhan  (excluding pregnants) | Same |

| He et al., | Retrospective study | February 1 and  March 29,  2020 | China | NA | 1031 | 1031 | NA | All consecutive inpatients who were hospitalized with laboratory-confirmed COVID-19 | NA |
| --- | --- | --- | --- | --- | --- | --- | --- | --- | --- |
| He et al., | Retrospective observational cohort | February 1,  2020, to  January 27,  2021 | United States | United States | 50,703 | 14,336 | 21,504 (test + validaion); 14,863  (prospective test data set) | Patients hospitalized with COVID-19  aged 18 and older, with a confirmed diagnosis or positive test of COVID-19 infection | Same (internal validation)  , but for prospective test were included patients with index date from September 7 to November 15, 2020 |
| Heber et al., | Observational cohort | Development: 7 January 2020  and 8 December 2020  Validation: 24 December 2020 and 07  April 2021; | Austria | Austria | 1126 (data not conclusive) | 679 (data not conclusive) | 447;  External validation: 341  (data not conclusive) | The model was developed and internally validated in a cohort of the Clinic Favoriten in Vienna, Austria.They had at least two blood samples and survived at least 4 days | Same (internal validation). The model was temporarily and externally validated in a mixed cohort consisting of additional patients from the Clinic Favoriten and in patients from the Department of Pulmonology, Kepler University Hospital, Linz, Austria |
| Heldt et al., | Retrospective study | January 1st and May 26th, 2020 | United Kingdom | United Kingdom | 619 | 193 (target) | 426 (control) | Hospitalized adult patients with a confirmatory diagnosis of COVID-19 by quantitative rtPCR | Same |

| Heller et al., | Cross- sectional study | Not clear | Germany | NA | 35 | 35 | NA | This study was conducted at two separate sites, including one public hospital involved in patient care and one research laboratory specialised in trace element analysis, i.e., the non- profit Public Hospital Klinikum Aschaffenburg-Alzenau, Germany, and the Institute for Experimental Endocrinology of Charité-Universitätsmedizin Berlin, Germany | NA |
| --- | --- | --- | --- | --- | --- | --- | --- | --- | --- |
| Her et al., | Cohort | January 20  2020 to April  30 2020 | South Korea | South Korea | 5628 | 3940 | 1688 | Patients with confirmed COVID- 19  infection who were admitted to 120 hospitals in Korea between January 20, 2020, and April  30, 2020. | Same (internal validation) |
| Hohl et al., | Observational cohort | March 1, 2020, and January 31, 2021 | Canada | Canada | 8761 | 6758 | 2054 | Patients adult (age ≥ 18 yr) nonpalliative patients with confirmed COVID-19 who presented to the emergency department of a participating site between Mar. 1, 2020, and Jan.  31, 2021. | Same (internal validation) |
| Hu et al., | Retrospective study | 28 January  2020 and 11  March 2020 | China | China | 247 | 183 | 64 (external validation) | Patients who had pneumonia confirmed by chest imaging, and had an ≤94% of oxygen saturation while they were breathing ambient air or a ratio of the partial pressure of oxygen to the fraction of inspired oxygen  at or below 300 mm Hg and laboratory-confirmed COVID-19 | Same |

| Ikemura et al., | Retrospective study | March 1 and  July 3, 2020 | United States | United States | 4313 | 3468 | 845 | All patients who tested positive for COVID-19 at our institution between  March 1 and July 3, 2020 | Same (20% derivation or training) |
| --- | --- | --- | --- | --- | --- | --- | --- | --- | --- |
| Incerti et al., | Cohort | February 20  June 5 2020 | United States | United States | 13,658 | 10,926 | 2732 | Patients were required to be older than 18 years old and have:   1. a U07.1 or U07.2 diagnosis, 2. a positive SARS-CoV-2 diagnostic test (eg, either   molecular or antigen tests) or (3) a B97.29 diagnosis with the absence of a negative SARS- CoV-2 molecular test within a  14-day window | Patients were randomly assigned to either training (80%) or test (20%) sets |
| Incerti et al., | Retrospective cohort study | February 20,  2020 and June  5, 2020 | United States | United States | 17,086 | 13,658 | 3,428 | Patients were required to be older than 18 years old and have:   1. a U07.1 or U07.2 diagnosis, 2. a positive SARS-CoV-2 diagnostic test (e.g., either   molecular or antigen tests) or (3) a B97.29 diagnosis with the absence of a negative SARS- CoV-2 molecular test within a 14-day window. | Patients were randomly assigned to either training (80%) or test (20%) sets. |
| Ismail et al., | Retrospective study | March 16 and  July 19, 2020 | United Arab Emirates | No | 371 | 371 | No | All adult patients (>18 years) admitted to the ICU of Al Ain Hospital between March 16 and July 19, 2020, with SARS-CoV- 2 infection confirmed using real- time reverse transcription  polymerase chain reaction (rt- PCR) on nasopharyngeal swabs | No |

|  |  |  |  |  |  |  |  | and radiologic evidence of respiratory infections |  |
| --- | --- | --- | --- | --- | --- | --- | --- | --- | --- |
| Ji et al., | Retrospective study | January 20 and  February 22,  2020 | China | China | 208 | 208 | Not clear | All the consecutive patients with COVID-19 admitted to Fuyang second people’s hospital or the fifth medical center of Chinese PLA general hospital clinical data | Same (internal validation) |
| Jimenez-Solem et al., | Prospective study | Between March 1st, 2020 and June  16th 2020 | Denmark | Denmark / External validation: United Kingdom | 3,944 | 3,944 | Not clear | All individuals undergoing a SARS-CoV-2 test (nasal and/or pharyngeal swap subjected to Real-Time Polymerase Chain Reaction testing) in the Capital and Zealand Regions (approximately 2.6 million citizens) of Denmark | Same |
| Jiwa et al., | Retrospective study | April and May, 2020 | United States | United States | 248 | 100 | 148 | All patients had been admitted and hospitalized with a clinical diagnosis and serological confirmation of COVID-19  infection. | Same |

| Kabootari et al., | Retrospective cohort study | February and August 2020 | Iran | Iran | 560 | 560 | Not clear | All adult patients (aged 18 years) with type 2 diabetes (n ¼ 560) who were hospitalised for COVID-19, according to the algorithms suggested by the WHO,16 at  a tertiary referral centre in Golestan province, Iran, between February and August 2020 | Same (internal validation) |
| --- | --- | --- | --- | --- | --- | --- | --- | --- | --- |
| Kamran et al., | Retrospective cohort study | Derivation: 1  January 2015  to 31 December 2019;  Validation: 1  March 2020 to  28 February  2021 | United States | United States | 33119 | 24419 | 8700 | Adults (≥18 years) admitted to hospital at Michigan Medicine, the academic medical center of the University of Michigan, with respiratory distress—that is, those admitted through the emergency department who received supplemental oxygen support. | The model was internally validated on adults (≥18 years) admitted to hospital at Michigan Medicine from 1 March 2020 to 28  February 2021 who required supplemental oxygen and had a diagnosis of COVID-19.  The external validation cohorts included adults (≥18 years) admitted to hospital at 12 external medical centers from 1 March 2020 to 28  February 2021 who required supplemental oxygen and had a diagnosis of COVID-19. |
| Kapoor et al., | Retrospective analysis | March 2020 to December 2020 | India | NA | 168 | 168 | NA | Lab-confirmed COVID-19 patients, with at least 18 years of age, from March 2020 till December 2020. | NA |

| Kar et al., | Retrospective cohort study | April to June 2020  (training), August to October 2020 (validation) | India | India | 2370 | 1393 | 977 | The participants were admitted to the hospital with symptoms and history suggestive of COVID with subsequent laboratory confirmation through RT-PCR tests. | Same |
| --- | --- | --- | --- | --- | --- | --- | --- | --- | --- |
| Karthikeyan et al., | Retrospective study | 10 January and  18 February  2020 | China | China | 375 | 300 (80%) | 75 (20%) | Hospitalized COVID-19 patients | Same |
| Katkat et al., | Retrospective and observational study | Not clear | NA | Turkey | 508 | NA | 508 | NA | Hospitalized patients with COVID-19 (≥ 18 years) |
| Kazemi et al., | Retrospective cohort | February 25,  2020 to April  25, 2020 | Iran | NA | 91 | 91 | NA | Adult patients with confirmed COVID-19 who had undergone CT scan <8 days from the beginning of symptoms, excluding the ones with RT-PCR more than 7 days from CT. CT score developed not based on the data. Authors tested CT score  and clinical variables in a model | NA |

| Kim et al., | Retrospective single-center cohort | February 19,  2020 to March  15, 2020 | Korea | NA | 38 | 38 | NA | Adult patients with confirmed COVID-19 admitted to a tertiary university hospital | NA |
| --- | --- | --- | --- | --- | --- | --- | --- | --- | --- |
| Kivrak et al., | Retrospective study | Not clear (they used a public database) | Italy | Italy | 1603 | 1603 | NA | Hospitalized COVID-19 patients | NA |
| Ko et al., | Not disclosed | January - July 2020 | China | South Korea | 467 | 361 | 106 | Hospitalized COVID-19 patients | Same |
| Kundi et al., | Cohort study | March 11,  2020, and June  22, 2020 | Turkey | NA | 18,234 | 18234 | NA | All hospitalized patients aged  ≥65 years old with at least one positive reverse transcriptase- polymerase chain reaction (RT- PCR) test for COVID-19 between March 11, 2020, and  June 22, 2020 | NA |

| Leoni et al., | Retrospective cohort study | February 22,  2020 to April  3, 2020 | Italy | Italy | 242 | 242 | Used Non- parametric bootstrap technique for resampling (1000x) | Consecutive critically ill patients admitted to the ICU with a positive result of real-time reverse transcriptase–polymerase chain reaction (RT-PCR) assay of nasal and pharyngeal swabs. | Same |
| --- | --- | --- | --- | --- | --- | --- | --- | --- | --- |
| Levy et al., | Retrospective and prospective cohort | March 1, 2020  to May 12,  2020 | United States | United States | 8391 | 6162 | 2229 | Adult patients with confirmed COVID-19 from 11 acute care hospitals in New York, from March 1, 2020 to April 23, 7 2020. Patients were excluded if they were still in the hospital at the study end point with a length of stay less than 7 days; if they were transferred to a hospital outside of the health system and their outcomes were unknown; or if they expired but were not marked as discharged in the EH | The same as the development cohort from another hospital in New York from March 1, 2020  to May, 7 2020, and all 12 hospitals from April 24, 2020 to May 6, 2020. |
| Li et al., | Retrospective cohort study | From first admission until April 10th 2020 | China | China | 4086 | 1780 | 1242 (Internal validation) / 1064  (External validation) | Hospitalized patients with confirmatory COVID-19 according to WHO guidelines | Same |
| Li et al., | Retrospective cohort | January 1 to  March 8, 2020 | China | China | 2039 | 1008 | 1031 | Hospitalized COVID-19 patients | Same |

| Liang et al., | Retrospective cohort | Between 21 November 2019 and 31  January 2020 | China | China | 1590 | 1272 | Internal: 318 External: Wuhan (940 patients), Hubei province (380 patients) and Guangdong province (73 patients) | Laboratory-confirmed hospitalized cases with COVID- 19 reported to the NHC | Same |
| --- | --- | --- | --- | --- | --- | --- | --- | --- | --- |
| Liang et al., | Retrospective cohort | November 21,  2019 to  January 31,  2020 | China | China | 2300 | 1590 | 710 | Patients with COVID-19 from 575 hospitals in 31 provincial administrative regions | Data from hospitals not included in the development cohort |
| Lin et al., | Not clear | Derivation: between January 10,  2020, and  February 24,  2020 /  Validation: February 2020  and July 2020 | China | Korea | 467 | 361 | 106 | COVID-19 patients in Wuhan, China | COVID-19 patients in 3 Korean medical institutions |
| Liu et al., | Retrospective cohort study | 9 February and  18 March 2020 | China | NA | 1751 | 1751 | NA | Patients with laboratory confirmation of COVID‐19 | NA |

| Liu et al., | Retrospective  , single- centre cohort study. | 10 January  2020 to 13  February 2020 | China | China | 308 | 308 | NA | All hospitalized patients diagnosed with COVID-19 | NA |
| --- | --- | --- | --- | --- | --- | --- | --- | --- | --- |
| Liu et al., | Retrospective cohort study | Between December 17,  2020, and  March 18,  2020 | China | NA | 216 | 216 | NA | Consecutive patients with solid tumors who had been diagnosed with COVID-19 and admitted to 32 hospitals in China from December 17, 2020, to March  18, 2020. Inclusion criteria were:  (1) pathologically confirmed solid tumor; (2) confirmed diagnosis of COVID-19 by  detection of SARS-CoV-2 via real-time PCR; (3) age >18 years old. | NA |
| Liu et al., | Retrospective study | From February 9th, 2020 to March 20th, 2020 | China | NA | 147 | 147 | NA | Consecutive patients with confirmed COVID-19 who were admitted to three temporarily organized Intensive Care Units (ICUs) of the Zhongfaxincheng campus of Tongji Hospital, affiliated to Huazhong University of Science and Technology in the city of Wuhan from February 9th, 2020 to March 20th, 2020, were  retrospectively analyzed | NA |

| Llanera et al., | Retrospective and observational cohort | 1 January to 30  June 2020 | England | No | 1004 | Not clear, sample sizes of 564, 245 and 195 for the training,  testing, and validation datasets | Not clear, sample sizes of 564, 245  and 195 for the training, testing, and validation datasets | All patients admitted to 7 hospitals in the region with COVID-19 between 01 January 2020 to 30 June 2020 with a known diagnosis either of type 1 diabetes, type 2 diabetes, other forms of diabetes, or newly diagnosed diabetes on admission were included irrespective of the  reason for admission | Same (internal validation) |
| --- | --- | --- | --- | --- | --- | --- | --- | --- | --- |
| Lopez-Escobar et al. | Cohort | March 1  trought June 10  2020 | Spain | NA | 2088 | 2088 | NA | Hospitalized patients with COVID-19 due to confirmed or suspected infection by  SARS-CoV-2 who were admitted to any of the 10 hospitals of the HM Hospitales group across different regions (including Madrid, Barcelona and Galicia) | NA |
| Lorente et al., | Cohort | Not clear | Spain | NA | 53 | 53 | NA | COVID + admitted in ICU | NA |
| Lu et al., | Retrospective observational study | January 25 through February 25,  2020 | China | NA | 344 | 344 | NA | Severe and critically ill patients (intensive care patients) who were diagnosed with COVID-19 and were hospitalized in Tongji hospital from January 25 through February 25, 2020. The illness severity of COVID-19 was defined according to the Chinese  management guideline for COVID-19 (version 6.0) | NA |

| Lu et al., | Retrospective single-center cohort | January 21,  2020 to  February 5,  2020 | China | NA | 577 | 577 | NA | Patients with confirmed or suspected COVID-19 from one hospital | NA |
| --- | --- | --- | --- | --- | --- | --- | --- | --- | --- |
| Luo et al., | Retrospective study | Between January 9,  2020, and  March 31,  2020 | China | NA | 1018 | 1018 | NA | The inclusion criteria were as follows: (a) adults (aged over 18 years), who understood and agreed to participate in this experiment and (b) PCR test was positive for virus nucleic acid of SARS-COV-2. | NA |
| Ma et al., | Retrospective study | January 12,  2020 to March  20, 2020 | China | China | 262 | 262 | Bootstrap method | All hospitalized patients diagnosed with COVID-19 | Same |
| Ma et al., | Single- centred, retrospective, observational study | Between 15  January and 15  March 2020 | China | China | 305 | 292 | 13 | Patients aged 14 years or older and patients who were diagnosed with COVID-19 pneumonia according to the interim  guidelines from the World Health Organization | Same |

| Machado-Alba et al., | Observational study | March and August 2020 | Colombia | No | 780 | 780 | No | All subjects of any age, sex and city of residence treated for COVID-19 between March 6 and August 31, 2020 were selected | No |
| --- | --- | --- | --- | --- | --- | --- | --- | --- | --- |
| Magro et al., | Retrospective cohort study | February 22nd and April 7th, 2020 | Italy | Italy | 2191 | 1810 | 381 | Hospitalized patients with a diagnosis of COVID-19 confirmed with a positive real- time reverse transcriptase polymerase chain reaction (RT- PCR) from nasal and pharyngeal | Same |
| Mahdavi et al., | Retrospective study | February 20th, 2020, and May  4th, 2020 | Iran | NA | 492 | 492 | NA | Hospital admission due to the initial diagnosis of COVID-19 infection by a physician according to the 5th Iranian COVID-19 guideline were randomly selected from the hospital’s patient data pool. | NA |
| Mancilla- Galindo et al., | Retrospective cohort study | 28 February  and 23 July  2020. | Mexico | Mexico | 856186 | 264026 | 592160 | All patients with a positive reverse transcription-polymerase chain reaction (RT-PCR)  for SARS-CoV-2 were included to maximise the power and generalisability of results | Only patients with a positive  RT-PCR for SARS-CoV-  2 and complete unduplicated data were included to validate the  model |

| Mann et al., | Retrospective observational study | March 5, 2020  and August 14,  2020 | United States | United States | 2193 | 1690 | 398 | Patients who tested positive for severe acute respiratory syndrome coronavirus 2 during hospitalization or were discharged with an ICD-10 code for COVID-19 (U07.1) were included. | Same |
| --- | --- | --- | --- | --- | --- | --- | --- | --- | --- |
| Manocha et al., | Observational cohort study | March 3 and  April 6, 2020 | United States | United States | 1053 | 446 | 440 | Consecutive patients with COVID‐19 were admitted to NYP/Weill Cornell Medicine and NYP/Lower Manhattan Hospital | Same |
| Marcolino et al., | Retrospective cohort | Derivation: March–July, 2020;  Validation: August– September | Brazil | Brazil (Spain: external validation) | 5506 | 3978 | Validation: 1054;  External validation: 474 | Consecutive patients (≥ 18 years) with confirmed COVID-19 admitted to the participating hospitals before 31 July | Consecutive patients (≥ 18 years) with confirmed COVID-19 admitted to the participating hospitals, from 01 August to 30  September 2020. Independent external validation was also performed in a cohort of patients from Vall d’Hebron University Hospital, in Barcelona, Spain, admitted from 01  March to 31 May 2020 |
| Martínez- Lacalzada et al., | Retrospective cohort | 23rd March to 21st May 2020 | Spain | Spain | 10433 | 7850 | 2583 | Consecutive patients hospitalized for COVID-19 (defined as a positive result on real-time reverse-transcription-PCR (RT-  PCR) for the presence of severe acute respiratory  syndrome coronavirus 2 (SARS- CoV-2) in | Similar |

|  |  |  |  |  |  |  |  | nasopharyngeal swab specimens or sputum samples), first hospital admission for COVID-19, and hospital discharge or in-hospital death |  |
| --- | --- | --- | --- | --- | --- | --- | --- | --- | --- |
| Mayneris- Perxachs et al., | Retrospective study | March 1st and May  1st, 2020 | Spain and Italy | Spain and Italy | 5345 | 3065 | 331 | Consecutive unselected patients who were admitted to the hospital and diagnosed with pneumonia by SARS-CoV-2 were included. In Primary Health care centers, all consecutive patients fulfilling  inclusion criteria were also analyzed | Same |
| Mei et al., | Retrospective cohort study | 8 January and  19 March  2020. | China | China | 1364 | 1088 | 276 | Hospitalized confirmed adult patients with COVID-19 by a real-time reverse transcription- PCR assay with nasal and pharyngeal swab specimens | Same |
| Mei et al., | Retrospective cohort | January 21,  2020 to  February 27,  2020 | China | China | 492 | 237 | Validation 1:  120 and  validation 2:  135 | Adult patients with confirmed COVID-19, diagnosed with pneumonia by CT scan, from one hospital in Wuhan. Patients who died within the first 24 hours, with not clinical outcome  available or who refused to participate were excluded | The same as the development population, from other 3 hospitals |

| Mendizabal et al., | Prospective cohort study | April 15 to  October 1 2020 | Latin American countries | NA | 2211 | 2211 | NA | Patients ≥17 years old, hospitalized with SARS-CoV-2 infection confirmed by the real- time polymerase method (RT- PCR) as per the local site- specific protocol | NA |
| --- | --- | --- | --- | --- | --- | --- | --- | --- | --- |
| Momeni- Boroujeni et al., | Retrospective query | February 2020 until the end of March 2020 | United States | NA | 553 | 553 | NA | Patients admitted to SUNY Downstate  Medical Center with COVID-19- related symptoms and confirmed Polymerase Chain Reaction (PCR)-positive | NA |
| Monterde et al., | Restrospectiv e Study | June 15 to  December 8  2020 | Spain | NA | 4607 | 4607 | NA | All COVID-19 hospitalizations reported in eight public hospitals of Catalonia (North-East Spain) | NA |
| Moulaei et al., | Retrospective study | February 9,  2020, to  December 20,  2020 | Iran | Iran | 1500 | 1500 | Not clear | Laboratory-confrmed COVID-19 hospitalized  patients (n=1500) were extracted from a database registry in Ayatollah Taleghani Hospital, afliated to Abadan  University of Medical Sciences | Same (internal validation) |

| Murri et al., | Retrospective study | March 5, 2020  to February 5,  2021 | Italy | Italy | 2384 | 921 | 1463 | All patients admitted to Fondazione Policlinico Gemelli with COVID-19 | Cohort of patients admitted between November 6, 2020, and  February 5, 202 |
| --- | --- | --- | --- | --- | --- | --- | --- | --- | --- |
| Nascimento et al., | Retrospective  ,  observational study | March to April 2020 | Brazil | NA | 105 | 61 | NA | Patients with COVID-19 in the intensive care unit (ICU) | NA |
| Neant et al., | Cohort | February 5 to  April 1, 2020 | France | NA | 655 | 655 | NA | Hospitalized patients with a laboratory-confirmed SARS-  CoV-2 infection  were enrolled in the French COVID cohort | NA |
| Nguyen et al., | Retrospective cohort study | March 20,  2020 and  December 29,  2020 | United States | United States | 1312 | 80% | 20% | Patients who (1) had a positive nasopharyngeal PCR test for SARS-CoV-2 and (2) were  admitted to the hospital within ± 3 days of the positive result. | Same |

| Nicholson et al., | Retrospective cohort | First patient to May 19, 2020 | United States | United States | 1042 | 578 | 464 | Consecutive adult patients with laboratory-confirmed COVID-19 patients from Mass General Brigham hospitals | Consecutive adult patients with laboratory-confirmed COVID-19 infection who were admitted for illness related to COVID-19 to four hospitals in the Mass General Brigham health care system (Brigham and Women’s Hospital, BWH; Newton Wellesley Hospital, NWH; Brigham and Women’s Faulkner Hospital, BWFH; and North Shore Medical Center, NSMC) in the  Boston region |
| --- | --- | --- | --- | --- | --- | --- | --- | --- | --- |
| Núñez-Gil et al., | Retrospective cohort | 8 February and  1 April, 2020 | Italy and Spain | Italy and Spain | 908 | Not clear | Not clear | All patients receiving attention in any health center with  in-hospital beds, who have been discharged or have died at the time of the evaluation who had a positive COVID-19 test or if their attending physicians considered them  highly likely to have presented the infection, | Same (internal validation) |
| Obremska et al., | Prospective cohort | Derivation: March and July 2020;  Validation: June and August 15,  2020. | Poland | Poland | 865 | 129 | Validation: 239;  Prospective study: 497 | Patients with e diagnosis of SARSCoV-2 infection confirmed by a reverse transcriptasepolymerase chain reaction test of a nasopharyngeal swab, from County Hospital in Boleslawiec.. | Patients with diagnosis of SARSCoV-2 infection confirmed by a reverse transcriptasepolymerase chain reaction test of a nasopharyngeal swab, from Regional Specialist  Hospital in Wroclaw |

| Oh & Song | Case control | January 1 to  June 4 2020 | South korea | NA | 2047 | 2047 | NA | Type 2 DM patients diagnosed with COVID-19 | NA |
| --- | --- | --- | --- | --- | --- | --- | --- | --- | --- |
| Oualim et al., | Retrospective study | March 21,  2020 to April  24, 2020 | Morocco | NA | 89 | 89 | NA | All adult patients (≥18 years old) who were diagnosed with COVID-19 according to WHO interim guidance, and confirmed by RNA detection of the SARS- CoV-2 in onsite clinical Laboratory | NA |
| Pan et al., | Retrospective cohort study | February 2 to  April 15 2020 | China | China | 123 | 98 | 25 | Critically ill laboratory- confirmed COVID-19 patients (ICU admission) | Same |
| Peiró et al., | Retrospective observational study | 16 March 2020  to 15 May  2020 | Spain | NA | 196 | NA | NA | Consecutive patients admitted to our emergency department with symptoms and confirmed laboratory test of COVID-19 and available concentrations of cardiac troponin I (cTnI), D-  dimer, CRP and LDH | NA |

| [Philippe](https://www.ncbi.nlm.nih.gov/pubmed/?term=Philippe%20A%5BAuthor%5D&cauthor=true&cauthor_uid=33449299) et al., | Bbi-centric cross- sectional study | March 13 and  June 26, 2020 | France | NA | 208 | 208 | NA | Adult (≥ 18-years old) hospitalized and ambulatory patients in two French hospitals with a positive test for RT-PCR for COVID-19 | NA |
| --- | --- | --- | --- | --- | --- | --- | --- | --- | --- |
| Pigoga et al., | Retrospective cohort | April 1 to  september 1  2020 | Sudan | Sudan | 467 | 374 | 93 | All patients were adults 18 years and over who presented to study sites between 01 April to 01 September 2020; met WHO criteria for a suspected, probable, or confirmed case of SARS- CoV-2 infection; and had a recorded disposition of either  death or discharge. | Same (internal validation) |
| Pimentel et al. | Retrospective observational study | COVID: 13th  March 2020 to 28th April 2020  Viral pneumonia: 2013 to 2017 | United Kingdom | NA | COVID: 497  Viral pneumonia: 485 | COVID: 497  Viral pneumonia: 485 | NA | The “COVID-19” cohort (CV) included patients admitted to the study hospitals from 13th March 2020 to 28th April 2020 with a positive COVID-19 RT-PCR test. The “viral pneumonia” (VI) cohort included patients admitted with viral pneumonia to the study hospitals for the years 2013 to 2017 | NA |
| Plečko et al., | Observational  , multicenter, retrospective cohort | March 2020  and April 2021 | Netherlands | Netherlands | 2901 | 1480 | 937 | Adults (>18 years) admitted to intensive care units (ICUs) with a confirmed SARS‐CoV‐2 infection, between March 2020 and April 2021 | Same |

| Ponce et al., | Observational  , prospective, longitudinal study | 1 May 2020  and 31 December 2020 | Latin America | Latin America | 870 | 697 | 173 (Internal validation cohort or test set, correspond to 20% sample) | Patients aged more than 18 years with SARS-CoV-2 infection confirmed by RT-PCR of nasopharyngeal swabs and acute kidney injury (AKI) from 1 May 2020 to 31 December 2020. | Same |
| --- | --- | --- | --- | --- | --- | --- | --- | --- | --- |
| Qeadan et al., | Retrospective cohort | January trought June 2020 | United States | NA | 22493 | 22493 | NA | Female patients aged 18-44 years old utilizing the Cerner COVID- 19 de-identified cohort | NA |
| Qin et al., | Retrospective study | February 04,  2020 to March  04, 2020 | China | NA | 247 | 247 | NA | All patients were laboratory confirmed COVID-19 cases, which met the diagnostic criteria for confirmed cases of the COVID-19 Diagnosis and Treatment Plan (Trial Seventh Edition) [7] issued by the National Health Commission of the People's Republic of China.  Patients were excluded based on the following criteria: patients younger than 18 years; time from illness onset to hospital admission exceeded 14 days; pregnant or parturient; patients  transferred to the “mobile cabin hospital” during hospitalization. | NA |

| Qin et al., | Retrospective multicenter cohort study | December 31,  2019 to March  4, 2020 | China | NA | 7106 | 7106 | NA | Patients diagnosed as COVID-19 and admitted to 9 hospitals in Hubei Province, China. COVID- 19 diagnosis was confirmed by at least one or both criteria of chest computed tomography manifestations and reverse transcription–polymerase chain reaction according to the New Coronavirus Pneumonia Prevention and Control Program (fifth edition) published by the National Health Commission of  China and World Health Organization interim guidance | NA |
| --- | --- | --- | --- | --- | --- | --- | --- | --- | --- |
| Romualdo et al., | Observational study | From March 14th to April 12th | Spain | NA | 66 | 66 | NA | Consecutive patients were admitted to our hospital by confirmed SARS-CoV-2 infection. COVID-19 was confirmed either by a positive result of real–time reverse transcriptase-polymerase chain reaction (RT-PCR) testing of a nasopharyngeal swab specimen or by positive result of serological testing and a clinically compatible  presentation | NA |
| Rothschild et al., | Not clear | Not clear | Israel | NA | 100 | 100 | NA | All patients had a diagnosis of COVID-19 confirmed by a polymerase chain reaction assay for severe acute respiratory syndrome-coronavirus-2 and underwent Speckle-Tracking Echocardiography examination  within 24 h of admission | NA |

| Ryan et al., | Retrospective study | Between 12  March and 12  April 2020 | United States | NA | 53001 | 53001 | NA | Patients aged 18 years or older | NA |
| --- | --- | --- | --- | --- | --- | --- | --- | --- | --- |
| Saldi et al., | Retrospective and prospective cohort study | July 2020 and  January 2021 | Indonesia | Indonesia | 1048 | 1048 | Not clear | All patients aged 18 years old and older  hospitalized with confirmed COVID-19 marked  by positive Reverse Transcriptase Polymerase Chain Reaction (RT-PCR) test | Same (internal validation) |
| Sankaranarayan an et al., | Retrospective electronic record study | March 1, 2020  to January 27,  2021 | United States | United States | 11807 | 9435 | 2372 | COVID-19 positive patients presenting to a Mayo Clinic site or health system | Same |
| Sauzay et al., | Retrospective derivation cohort | Derivation: February 21 to  March 30  2020;  Validaton: October 19th, 2020 to November  17th, 2020 | France | France | 235 | 154 | 81 | All consecutive adult patients (≥ 18 years old) admitted from February 21 st, 2020 to March 30th, 2020 in University Hospital (CHU) of Amiens and diagnosed with COVID-19 according to the viral detection of SARS-CoV2 (PCR). | All consecutive adult patients admitted from October 19th, 2020 to November 17th, 2020 in the same hospital and diagnosed with COVID- 19 with a similar test. |

| Schlauch et al., | Retrospective study | March to September 2020 | United States | United States | 46971 | 23485 | 23485 | Patients hospitalized with laboratory-confirmed SARS- CoV-2 infection, representing COVID-19 disease, at 176 HCA Healthcare-affiliated facilities across the Unites States between March 2 and September 23 and  were followed through October 7, 2020 | Same (50% testing) |
| --- | --- | --- | --- | --- | --- | --- | --- | --- | --- |
| Schöning et al., | Retrospective and observational External validation: prospective | Derivation: Between February 1st and August 31st Internal validation:  September 1st through  November 16th 2020 | Switzerland | Switzerland | 16418 | 158 | Internal validation: 40 External validation: 459 | Patients testing positive with sufficient data available | Same |
| Sensusiati et al., | Retrospective study | March 13 to  May 15, 2020 | Indonesia | NA | 111 | 111 | NA | (1) Patients with a chief complaint of one COVID-19-related symptom, including:  fever, dry cough, tiredness, aches and pains, nasal congestion, headache, conjunctivitis, sore throat, diarrhea, loss of taste or smell, a rash on skin, or discoloration of fingers or toes (WHO, 2020); (2) confirmed SARS-CoV-2 infection  by reverse-transcriptase | NA |

|  |  |  |  |  |  |  |  | polymerase chain reaction (RT PCR) using nasopharyngeal and oropharyngeal specimens; (3) patients who underwent chest x- ray (CXR) on the day of admission to the hospital. |  |
| --- | --- | --- | --- | --- | --- | --- | --- | --- | --- |
| Shah et al., | Prospective observational sutdy | March 16 to  June 7, 2021 | Scotland | No | Not clear | Not clear | No | Not clear | No |
| Shamout et al., | Retrospective cohort | Between March 3, 2020  and May 13,  2020 | United States | United States | 19957 chest X-ray exams collected from 4,722 patients | 19957 chest X-ray exams collected from 4,722 patients | 718 patients  and 832 images | The dataset consists of chest X- ray images collected from patients who tested positive for COVID-19 using the polymerase chain reaction (PCR) test, along with the clinical variables recorded closest to the time of image acquisition | Same |
| Shang et al., | Cohort | February 26 to  March 10,  2020 | China | NA | 159 | 159 | NA | Patients with confirmed COVID- 19 with more than 18 yrs | NA |

| Shang et al., | Retrospective Cohort | January 1,  2020 to March  27,2020 | China | China | 452 | 113 | 339 | Consecutive patients with confirmed COVID-19 from 2 hospitals in Wuhan, who had severe or critical illness | The same definition as the development population, but from a third hospital in Wuhan |
| --- | --- | --- | --- | --- | --- | --- | --- | --- | --- |
| Shao et al., | retrospective cohort study | Februry 4 to  April 10, 2020 | China | NA | 263 | 263 | NA | Patients confirmed COVID-19 with liver injury | NA |
| Shayganfar et al., | cohort | Not disclosed | Iran | NA | 176 | 176 | NA | COVID + suspected patients | NA |
| Singh et al., | Retrospective cohort | Georgia and United States- Texas: March 1, 2020 and  August 8,  2020;  Spain: February 24,  2020 and May  19, 2020;  United States_New  York: March | Spain (3 hospitals), Georgia (6 hospitals), United States- Texas (7 hospitals) and United States- New York (8 hospitals) | United States | 11,321 | 7028 | Testing: 3554;  External validation: 739. | Patients admitted due to a confirmed SARSCoV-2 infection from La Paz University Hospital (La Paz) in Madrid, Spain (3 hospitals), Emory Healthcare System (Emory) in Atlanta, Georgia (6 hospitals), Houston Methodist Hospital System (Houston Methodist)  in Houston, Texas (7 hospitals) and Mount Sinai Health System (Mount Sinai) in New York,  New York (8 hospitals). | Same (internal validation), while for external and independent data from Mount Sinai were publicly available and obtained via IMMPORT shared data repository (see https://[www.immport.org/](http://www.immport.org/)  ) |

|  |  | 21, 2020 and  June 23, 2020 |  |  |  |  |  |  |  |
| --- | --- | --- | --- | --- | --- | --- | --- | --- | --- |
| Sinkovits et al., | Cohort | Not disclosed | Turkey | NA | 102 | 102 | NA | Adult (age >18 years) cohort of SARS-CoV-2  infected hospitalized patients | NA |
| Sîrbu et al., | Observational study (not defined) | 3rd of March 2020 and the 30th of April 2020, and e 3rd of September and the 24th of December 2020 | Italy | Italy | 823 | 313 | 510 | COVID19 patients hospitalised during the frst COVID-19 wave at the Pisa University Hospital, in Italy | Same (internal validation) |
| Soto-Mota et al., | Observational  prospective study | September and  December 2020 | Mexico | Mexico | 166 | NA | NA | Consecutive hospital admissions  (for RT-PCR-confirmed COVID- 19 infection) | NA |

| Soto-Mota et al., | Retrospective Cohort | April 30, 2020  to May 20,  2020 | Mexico | NA | 400 | Score developed by consensus | 400 | NA | Consecutive patients with confirmed COVID-19 from 12 hospitals, with complete clinical information and outcome |
| --- | --- | --- | --- | --- | --- | --- | --- | --- | --- |
| Sottile et al., | Prospective and retrospective cohort | March 2020  and July 2020 | United States | United States | 27,296 | 21,837 | 5,459 | All patients (with and without COVID-19)>14 years old hospitalized during the study period without a do not resuscitate order | COVID-19 positive cohorts |
| Sottile et al., | Retrospective and prospective cohort | RETROSPEC TIVE:  Between August 2011  March 4, 2020  \| PROSPECTIV  E: March 15th 2020 - July  2020 | United States | United States | 109,383 | 82,087 | 27,296 (1,358  SARS-CoV-2  positive) | Patients >14 years old hospitalized at any of UCHealth’s 12 acute care hospitals. Admission to either an intensive care unit (ICU) or intermediate care unit. | Same |
| Sourij et al., | Prospective and retrospective study | 15 April 2020  to 30 June  2020 | Austria | Austria | 238 | Not clear | Not clear | People aged 18 years or older with a confirmed positive throat swab for SARS-CoV-2 and a confirmed diagnosis of type 1 diabetes, type 2 diabetes or prediabetes were included in the registry (either known or newly  iagnosed) | Same (internal validation) |

| Stachel et al., | Retrospective cohort study | 3 March 2020–  28 April 2020 | United States | United States | 3395 | 2054 | Internal validation: 477 / external temporal validation: 864 | Adult inpatients with admitted and subsequently discharged either alive or dead during the study period | Same |
| --- | --- | --- | --- | --- | --- | --- | --- | --- | --- |
| Sun et al., | Retrospective cohort study | 10 January and  18 February  2020 | China | China | 485 | 375 | 110 | Patients’ blood samples collected at a chinese hospital | Same |
| Surme et al., | Cohort | March 9 to  April 8, 2020 | Turkey | NA | 336 | 336 | NA | Patients aged ≥18 years who were diagnosed with COVID-19 pneumonia by the Department of Infectious Diseases and Clinical  Microbiology between March 9 and April 8, 2020.  SARS-CoV-2 testing was performed through real-time reverse transcription-polymerase chain reaction | NA |
| Synolaki et al., | Cohort | March 10 to  July 7 2020 | Greece | Greece | 235 | 117 | 118 | Consecutive COVID-19 patients hospitalized at University Hospital, Alexandroupolis and “AHEPA” Hospital,  Thessaloniki from March 10, 2020 and had an outcome until July 7, 2020 | Not clear |

| Thomson et al., | Prospective observational cohort study | March to May 2020 with follow-up to June 2020 | United Kingdom | NA | 156 | 156 | NA | Severely ill patients with laboratory-confirmed COVID-19 | NA |
| --- | --- | --- | --- | --- | --- | --- | --- | --- | --- |
| Turrini et al., | Retrospective cohort | Not clear | Italy | NA | 205 | 205 | NA | Patients aged between 17 and 100 years ahopitalized for SARS-Cov-2 pneumonia | NA |
| Vagliano et al., | Prospective study | February 15th and July 1st 2020 | Netherlands | Netherlands | 2690 | 2,69 | No | All patients admitted between February 15th and July 1st 2020 with confirmed COVID-19 to a Dutch ICU extracted from the Dutch National Intensive Care Evaluation (NICE) registry. | Same (internal validation) |
| Van Dam et al., | Retrospective analysis | 3 March and  until 25 May  2020 | Netherlands | NA | 642 | 642 | Not clear | Adults admited to  the hospital with symptoms suggestive of COVID-19 and positive result of the PCR or (very) high suspicion of COVID-19 according to the  chest CT scan | Not clear |

| Varol et al., | Single‐centre case cohort study | March 11,  2020 and May  15, 2020 | Turkey | NA | 383 | 383 | NA | All hospitalized patients diagnosed as confirmed or probable COVID‐19 pneumonia | NA |
| --- | --- | --- | --- | --- | --- | --- | --- | --- | --- |
| Vela et al., | Retrospective study | March 1 and  September 15,  2020  (development period), September 16 and December 27, 2020  (validation period) | Spain | Spain | 7718329 | 7.5 million | 218,329 | Data from the entire population of Catalonia | Individuals with PCR- confirmed COVID-19, who were  infected after developing the model |
| Velasco- Rodriguez et al., | Cohort | February 27 to  April 17, 2020 | Spain | Spain | 2070 | 1270 | 800 | Consecutive hospitalized adult patients with  confirmed moderate or severe COVID-19 from four hospitals | Not clear |
| Vepa et al., | Retrospective  , case-control analysis. | 01/03/2020 and  22/04/2020 | United Kingdom | United Kingdom | 355 | 70% | 30% | Adult patients diagnosed with positive RT-PCR nasopharyngeal swabs or CT scans with changes  suggestive of COVID-19 | Same |

| Verma et al., | Retrospective cohort study | Between November 1,  2019, and June  30, 2020 | Canada | NA | 1027 | 1027 | NA | All adults over 18 years admitted to an inpatient medical service or medical–surgical ICU, including coronary care units, and discharged between Nov. 1, 2019, and June 30, 2020 | NA |
| --- | --- | --- | --- | --- | --- | --- | --- | --- | --- |
| Vicka et al., | Retrospective study | 2020 | No | Lithuania | 249 | No | 249 | No | Patients who were admitted to a tertiary referral university hospital in the  year of 2020 and tested positive for SARS-CoV-2. |
| Villa et al., | Cohort | Between March 1 and  May 31, 2020 | Italy | Italy | 249 | 187 | 62 | PCR + COVID inpatients | Same |
| Wang et al., | Multicenter Retrospective Study | Not disclosed | China | NA | 275 | NA | NA | Consecutive adult patients (aged  ≥18 years) from 13 designated ICUs with confirmed with SARS-CoV-2 infection by quantitative polymerase chain reaction test of throat swab samples or sputum samples  according to the WHO guidance. | NA |

| Wang et al., | Multicenter retrospective study | January 31,  2020, to April  17, 2020 | China | NA | 119 | 119 | NA | Hospitalized patients with laboratory confirmed COVID-9 by Rt-PCR. Patients with comorbidities, pregnant women and patients younger than 18 years old were excluded | NA |
| --- | --- | --- | --- | --- | --- | --- | --- | --- | --- |
| Wang et al., | Cohort | Derivation: January 7,  2020, 17:58 31  to February 11,  2020, 22:01  Validation: January 1,  2020, to  February 20,  2020 | China | China | 296 | 296 | 44 | Consecutive patients diagnosed with  29 COVID-19 in the First People’s Hospital of Jiangxia District in Wuhan | Randomly  33 collected patients with COVID-19 who had been hospitalized in the Infection department of 34 Union Hospital in Wuhan |
| Wang et al., | Cohort | January to 25  Februay 24  2020 | China | NA | 134 | 134 | NA | COVID + patients | NA |
| Wang et al., | Retrospective study | Between January 1 and  August 7, 2020 | United States | United States | 3740 | 3366 | 374 | Patients who tested positive with COVID-19 and were treated at NYU (New York University)  Langone Health | Same |

| Wang et al., | Retrospective single-center cohort | January 28,  2020 to March  4, 2020 | China | China | 243 | 199 | 44 | Adult patients with confirmed COVID-19 from one university hospital | Data for enrolled patients were partitioned into two complementary subsets: the training set of patients from four wards was used to establish the predictive model, and the testing set of patients from another ward was used to validate  the analysis. |
| --- | --- | --- | --- | --- | --- | --- | --- | --- | --- |
| Wang et al., | Retrospective study | January 6,  2020 and  March 6, 2020 | China | China | 400 | 261 | 68 (test) ;  61 (external test) | Patients from Renmin Hospital of Wuhan University (Wuhan dataset) were randomly assigned to a training set (n = 271) and a test set (n = 68) at a  ratio of 4:1. | Patients from Renmin Hospital of Wuhan University (Wuhan dataset) were randomly assigned to a training set (n = 271) and a test set (n  = 68) at a  ratio of 4:1. Patients from the other three centers constituted an  external test set (n = 61). |
| Weng et al., | Retrospective cohort | January 1,  2020 to  February 15,  2020 | China | China | 301 | 176 | 125 | Adult patients with laboratory- confirmed COVID-19 from 2 hospitals | The same as the development population (the criteria used to divide patients in training and testing sets was not clear) |
| Williams et al., | Retrospective cohort | Development cohort: any time prior to 2020;  validation cohort: January 1st 2020 to  April 20, 2020 | United States, South Korea, Spain, Australia, Japan, Netherlands | South Korea, Spain, United States | 2.126.784 | 2,082,277 | 44.507 | Healthcare database of 6 countries, in which adult patients with GP, EP or OP visit with influenza or flu-like symptoms, at least 365 days of prior observation, and no symptoms in the preceding 60 days | Adult patients with confirmed with COVID- 19, presenting at an initial healthcare provider interaction in a GP, ER or OP visit, and who had no diagnosis of influenzae or  pneumonia and no flu-like |

|  |  |  |  |  |  |  |  |  | symptoms in the preceding 60 days |
| --- | --- | --- | --- | --- | --- | --- | --- | --- | --- |
| Wongvibulsin et al., | Cohort | March 5 to  December 4  2020 | United States | United States | 3494 | Not clear | Not clear | Not clear | Not Clear |
| Wu et al., | Single-center retrospective study | January 27,  2020, to  February 26,  2020 | China | China | 270 | 210 | 60 | Patients infected with laboratory-identified SARS- CoV-2 | Same |
| Xie et al., | Retrospective cohort | January and February 2020 | China | China | 444 | 299 | 145 | Patients with confirmed COVID- 19 from one hospital in Wuhan who had been discharged or died | Patients with confirmed COVID-19 from another hospital in Wuhan,  excluding 6 patients who died quickly |

| Yan et al., | Retrospective cohort study | February 17 to  March 22,  2020 | China | NA | 2569 | 2569 | NA | Consecutive patients with highly suspecting COVID‐19 were admitted to Wuhan Third Hospital & Tongren Hospital of Wuhan University | NA |
| --- | --- | --- | --- | --- | --- | --- | --- | --- | --- |
| Yasukawae et al., | Cohort | April 18 to  May 9 2020 | United States | NA | 113 | 105 | NA | PCR + COVID inpatients | NA |
| Yavuz et al., | Cohort | March 8 to  April 8 2020 | Turkey | NA | 113 | 113 | NA | Confirmed cases of SARS-CoV- 2 pneumonia  aged 60 and over admitted to the emergency department of the University of Health Sciences Haydarpasa Numune Training and Research Center during a month from March 8, 2020 | NA |
| Yoo et al., | Retrospective cohort | March 1, 2020  to April 28,  2020 | United States | United States | 4.840 | 1.613 | 1.614 | Adult patients with confirmed COVID-19 from 5 hospitals, up to 99 years-old. The sample was randomly split in 3 datasets, the  second one was used for development | The same as the development population: randomly split in 3 datasets, the third one was used for validation |

| Yu et al., | Retrospective cohort study | 12th and 19th February 2020 | China | NA | 141 | 141 | NA | Consecutive hospitalized patients aged ≥75 years with at least one positive test result of the pathogen | NA |
| --- | --- | --- | --- | --- | --- | --- | --- | --- | --- |
| Yu et al., | Cohort | February 1 to  May 4 2020 | United States | United States | 3491 | 2793 | 698 | Hospitalized COVID-19 in- patients | Same |
| Zayat et al., | Retrospective  , single- center study | March 1, 2020  to April 20,  2020 | Germany | NA | 17 | 17 | NA | All adult inpatients (≥18 years old) who were diagnosed with COVID-19, according to the WHO interim guidance, who developed severe COVID-19 disease with ARDS, requiring ECMO support | NA |
| Zeng et al., | Cohort | January 9 to  March 25 2020 | China | China | 351 | 246 | 105 | Consecutive COVID-19 patients were those admitted to Third People’s Hospital of Yichang between 9  January to 25 March 2020. | Same |

| Zhang et al., | Single-center, retrospective, observational study | January 29,  2020, to March  23, 2020 | China | NA | 352 | 352 | NA | Patients from Jan 29, 2020, to  Mar 23, 2020, who had been diagnosed with COVID-19, according to WHO interim guidance | NA |
| --- | --- | --- | --- | --- | --- | --- | --- | --- | --- |
| Zhang et al., | Retrospective cohort | January 12,  2020 to  February 9,  2020 | China | China | 828 | 516 | 312 | Adult patients with confirmed COVID-19 from one hospital | Adult patients with confirmed COVID-19 from the same hospital in a different time span (February 8-9, 2020) and from another hospital |
| Zhang et al., | Retrospective cohort | Not reported | China | United Kingdom | 1001 | 775 | 226 | Adult patients with confirmed COVID-19 from one hospital | Adult patients with confirmed COVID-19 from another hospital |
| Zhao et al., | Retrospective study | March 9, 2020 to April  20, 2020 | United States | United States | 641 | 454 | 187 | Hospitalized patients with laboratory-confirmed COVID- 19, with age >= 18 years old | The same (70% for training and 30% for testing) |

| Zhou et al., | Retrospective multicentre cohort study | December 29,  2019 and  January 31,  2020 | China | NA | 191 | 191 | No | All adult inpatients (≥18 years old) with laboratoryconfirmed COVID-19 from Jinyintan Hospital and Wuhan Pulmonary Hospital (Wuhan, China) who had been  discharged or had died by Jan 31, 2020. | No |
| --- | --- | --- | --- | --- | --- | --- | --- | --- | --- |
| Zhou et al., | Retrospective single-center cohort | January 12,  2020 to  February 26,  2020 | China | NA | 118 | 118 | NA | Elderly patients (>60 years) with "clinically diagnosed" COVID- 19 (RT-PCR or chest CT) from one university hospital | NA |
| Zhu et al., | Cohort | January 18 to  March 7, 2020 | China | NA | 180 | 180 | NA | In-hospital patients confirmed COVID-19 from January 18, 2020, to March 7, 2020 | NA |
| Zinellu et al., | Retrospective study | Between 15  March and 15  May 2020 | Italy | NA | 105 | 105 | NA | Consecutive COVID‐19 patients admitted to the Respiratory Disease and Infectious Disease Units of the University Hospital of Sassari and the Pneumology Unit of the Santissima Trinità Hospital of Cagliari, Sardinia,  Italy | NA |

**Table S1. Continued**

| **Author** | **Model outcome** | **Outcome time** | **Variables collected on admission?** | **Original modelling approach** | **Imputati on** | **Use of AI techniques** | **Was a score produced?** |
| --- | --- | --- | --- | --- | --- | --- | --- |
| Abbasi et  al., | Mortality | In-  hospital | Yes | Multivariate regression analysis | No | No | No |
| Abdollahpo  ur et al., | Mortality | In-  hospital | Yes | Logistic + XGboost | No | Yes | No |
| Abdulaal et al., | In-patient death | In- hospital | Yes | Artificial neural network (ANN) | No | Yes | No |
| Acar et al., | Mortality | In- hospital | Yes | Logistic | No | No | Yes |
| Adderley el at., | Death within 28 days of admission | Secondar y care (in- hospital or post-  discharge  ) | Yes | Logistic regression, Least absolute shrinkage and selection operator  (LASSO, l1 penalised) and Gradient boosted model | Yes | No | No, this study externally validate the ISARC 4C score |
| Agarwal et al., | Severity and mortality | In- hospital |  | Multivariate logistic regression | No | No | No |
| Ageno et al., | Composite of need for non- invasive ventilation, need for orotracheal  intubation, or death | In- hospital | No, first day after admission | Backward Selection, Least Absolute Shrinkage and Selection Operator (LASSO), and Random Forest. | Yes | Yes | Yes |

| Akdur et al., | Time-to-death within 14 days and 90 days of ED admission | 14 and 90-days after hospital  admissio n | Yes | Logistic regression and Cox proportional hazard models | No | No | No (they tested existing scores) |
| --- | --- | --- | --- | --- | --- | --- | --- |
| Aliberti et al., | Mortality | Either in hospital or outside  hospital | Yes | Cox proportional hazards models | No | No | Yes |
| Allenbach et al., | Composite of  ICU admission or death | 14 days | Yes | Multivariate logistic regression | Yes | No | Yes |
| Altini et al., | Mortality or admission to the intensive  care unit | In- hospital | Not clear | Decision tree (DT), random forest (RF), Gaussian naive Bayes (GNB), support vector machines (SVM), K-nearest neighbors (KNN),  and adaptive boosting | Yes, KNNImp uter  algorithm | Yes | No |
| Altschul et  al., | Mortality | In-  hospital |  | Multivariate logistic regression | No | No | Yes |
| Avendaño‐  Ortiz et al., | Mortality | In-  hospital | Not clear | Univariate regression and binary logistic regression  mode | No | No | Yes |
| Aznar- Gimeno et al., | ICU admission or mortality | 30 days after hospital  admissio n | Yes | multilayer perceptron, random forest, and extreme gradient boosting (XGBoost) | Yes | Yes | Yes (https://github.com/ITAINNOVA/COVID  _IIS) |
| Bai et al., | Mortality | In-  hospital | Yes | Random Forest-based machine learning | Yes | Yes | No |
| Banoei et al., | Hospital Mortality | In- hospital | Yes | Statistically inspired modification of partial least square (SIMPLS) analysis, an algorithm of PLS (a linear machine learning method). A Latent class analysis (LCA) was carried to cluster the patients with COVID-19 to identify low- and high-risk patients. | Yes | Yes, an algorithm of PLS (a linear machine learning  method) | No |
| Baqui et al., | Death | In-  hospital | Not clear |  | No | Yes | No |

|  |  |  |  | XGBoost, K-Nearest  Neighbors, Neural Network, Random Forest and Support Vector Machine algorithms algorithm and logistic regression. |  |  |  |
| --- | --- | --- | --- | --- | --- | --- | --- |
| Bartoszko et al., | Risk of mortality from the  day 0 until day 15 and beyond, assessed dynamically every  three days | In- hospital | Also, but all data were collected at the time of ICU  admission (day 0) and in three-day intervals thereafter (day 3, 6, 9, 12, 15)  until the patient was discharged  from ICU | Univariate analysis, generalized estimating equations (GEE), HosmerLemeshow test and  bootstrapping | Yes, multiple imputatio n | No | Yes |
| Bello-  Chavolla et al., | Mortality | 30 days |  | Cox proportional risk regression analysis | No | No | Yes |
| Bello- Chavolla et al., | Severe COVID-19 as  a composite of death, ICU admission or requirement for intubation | 7, 10, 15,  20 and 30 days (main focus on day 15) | Yes | Elastic Net Cox regression | Yes | No | Yes |
| Bennouar et al., | In-hospital mortality | In- hospital | Yes | Kaplan-Meier survival curve, proportional Cox regression analyses | No | No | No |
| Bennouar et al., | Mortality/ICU/ MV/NIMV | 28 days | Yes | Cox | No | No | Yes (age, natremia, BUN, CRP, NLR, LDH, albumin) |

| Berenguer et al., | Mortality | 30 days | yes | Multivariable logistic regression | No | No | Yes (age, SaO2 adjusted by age, neutrophil to lymphocyte ratio; GFR; Dyspnea; and sex) |
| --- | --- | --- | --- | --- | --- | --- | --- |
| Berry et al., | Death due to any cause within 40 days of hospital  admission | 40 days after hospital admissio  n | Yes | Multivariate proportional hazard models | No | No | Yes |
| Bertsimas et al., | In-hospital mortality | In- hospital | Yes | XGBoost algorithm (model of choice). Comparison of XGBoost with two other ML methods: Logistic  regression and CART | Yes | Yes | Yes |
| Boudou et al., | Hospitalization  , intensive care and death | Not clear | Not clear | Generalised linear models and recursive partitioning and regression trees | No | No | No |
| Brieghel et al., | Mortality | 90 days | Admission and trought  hospital stay | Univariable and multivariable Cox regression | No | No | No |
| Cai et al., | 60-day all-  cause mortality | 60-days | Yes | LASSO, Cox Regression | Yes | No | No |
| Castro VM, McCoy TH, Perlis RH | (1) a composite  severe illness outcome, including admission to the intensive care unit (ICU),  mechanical ventilation, and (2) mortality | In- hospital | Not clear | Least absolute shrinkage and selection operator risk mode | Yes | No | No |
| Chen et al., | Mortality | 14, 21  and 28 days |  | Multivariate Cox regression analysis | No | No | Yes (nomogram) |

| Cheng et al., | Mortality | In- hospital | Yes | Univariate and multivariate Cox regression model | No | No | Yes |
| --- | --- | --- | --- | --- | --- | --- | --- |
| Cho et al., | Mortality (other 5 levels  of severity) | 14 and 28 days | Yes | COX | No | No | Yes (age, sex, comorbities, lymphocite count) |
| Chow et al., | Critical disease (ICU  admission, ventilation, and/or death) | Follow- up of outcomes for a minimum of 10  days | Not clear | Multivariable logistic regression model | Yes | No | No |
| Chua et al., | Mortality | In-  hospital | Not clear | Logistic regression | Yes  (MICE) | No | Yes (SpO2; obesity; Age; RR; stroke) |
| Chung et al., | Mortality | Not clear | Yes | AdaBoost, random forest, and eXtreme Gradient  Boosting (XGBoost); 5-layer deep neural network (DNN) | Yes | Yes | No |
| Chuperk et al., | Mortality | 28 days | Partially, there are variables that includes organ support in the first 2 weeks | XGBoost tree Machine learning model | Yes (Bagged forest) | Yes | No |
| Clift et al., | Mortality | Either in hospital  or outside hospital | Yes | LASSO | Yes | No | Yes |

| Corradi et al., | MV and/or mortality | In- hospital | Yes | Logistic | No | No | No |
| --- | --- | --- | --- | --- | --- | --- | --- |
| Covino et al., | Mortality | In- hospital | Admission and trought hospital stay | COX | No | No | No |
| Dashti et al., | Hospitalization and mortality | In- hospital | Yes | Logistic regression model and generalized linear model (GLM) | No | Yes (generalized linear model (GLM)) | Yes |
| Deng et al., | In-hospital mortality | In- hospital | Yes | Multivariable logistic regression | No | No | No |
| Di Castelnuov o et al., | Mortality | In- hospital | Yes | Cox proportional-hazards regression models and Random Forest | Yes | Yes (Random Forest) | No |
| Ding et al., | Mortality | In- hospital | Yes | Cox proportional hazards model | Yes | No | Yes (nomogram) |

| Doganci et al., | Mortality | In- hospital | Yes | Logistic regression models | No | No | Yes |
| --- | --- | --- | --- | --- | --- | --- | --- |
| Doher et al., | 60-day mortality after ICU admission | 60-day after ICU admissio n | ICU  admission | Generalized linear modelling with binomial distribution | Yes | No | No |
| Dominguez  -Olmedo et al., | Mortality | In- hospital | Yes | Extreme gradient boosting algorithm, Shapley Additive Explanations | No | Yes | No |
| Ebrahimi et al., | Overall survival | In- hospital | Yes | Elastic-net regularized Cox proportional hazards (PH) regression and model approximation via backward elimination | No | Yes | No |
| El-Raheem et al., | Mortality | In- hospital |  | Logistic regression model | No | No | No |
| Eskandar et al., | in-hospital deaths | in- hospital | Yes | Multivariate logistic regression | No | No | Yes |

| Faisal et al., | Mortality | In- hospital |  | Multivariable logistic regression | No | No | Yes (CARMc19_N and CARMc19_NB) |
| --- | --- | --- | --- | --- | --- | --- | --- |
| Fan et al., | Mortality | In- hospital | Admission and from hospitalization | Logistic regression | No | No | Yes |
| Fernandes et al., | Intensive Care Unit (ICU) admission, use of mechanical ventilation or death | In- hospital | Yes | Artificial neural networks, extra trees, random forests, catboost, and extreme gradient boosting | Yes | Yes | No |
| Fumagalli et al., | Mortality | In- hospital | Yes | Cox regression analysis | No | No | Yes |
| Galiero et al., | In-hospital mortality | In- hospital | Yes | Multivariate logistic regression | Yes | No | No |
| Galloway et al., | Composite of  transfer to ICU or death | In- hospital |  | LASSO logistic regression | No | No | Yes |

| Garrafa et al., | In-hospital mortality | In- hospital | Yes | Random forest, GBM, Logistic regression | Yes | Yes | Yes |
| --- | --- | --- | --- | --- | --- | --- | --- |
| Gatti et al., | Mortality | In- hospital | Not clear | Logistic regression | No | No | No |
| Giradin et al., | Mortality | In- hospital | Yes | COX and Random forest | No | Yes | No |
| Goméz et al., | Mortality | 30 days |  | Multivariable logistic regression | No | No | Yes (COVEB) |
| Gopalan et al., | Mortality or recovery | In- hospital | Yes | Univariate analysis and multivariable logistic regression | No | No | Yes, OUR ARDs’ score |
| Gorham et al., | ICU mortality | In- hospital | Yes | Generalized mixed model | No | No | No |

| Guan et al., | Death | In- hospital | | Yes | LASSO, XGBoost | Yes | Yes | No |
| --- | --- | --- | --- | --- | --- | --- | --- | --- |
| Gude- Sampedro et al., | death of any cause after RT- PCR diagnosis in the study period. | Study period | | Yes | multivariable logistic regression | No | No | Yes |
| Gue et al., | Mortality | 30 days | | Yes | Multivariable logistic regression | No | No | Yes (COVID-19 Mortality Socre) |
| Gupta et al., | Composite outcome  - in-hospital clinical deterioration, comprising any of the following: initiation of ventilatory support (non-invasive ventilation, invasive mechanical ventilation, or extracorporeal membrane oxygenation); admission to a high- ependency or intensive care unit;  or death. | | In- hos pital | Yes | Multivariable logistic regression | Yes | No | Yes |

| Gupta et al., | Intensive care unit patient management, number of ventilator days, maximal number of pressors/inotro pes, sequential organ failure assessment score, and significant clinical  organ-specific events encompassing cardiac events (myocardial injury, reductions in contractility, and arrhythmias), renal injury,  hepatic injury, thrombotic events, and death | In- hospital | Also, but all data were collected at the time of ICU  admission (day 0) and in three-day intervals thereafter (day 3, 6, 9, 12, 15)  until the patient was discharged from ICU | Univariate analysis, multivariable logistic regression, COX proportional hazards modelsleast absolute shrinkage  and selection operator (LASSO) regression | Yes, multiple imputatio n | No | Yes, AAALLPPPACA Risk Stratification Scorea |
| --- | --- | --- | --- | --- | --- | --- | --- |
| Hajifathalia n et al., | Mortality | 7 days  and 14 days | Yes | Multivariable logistic regression | Yes.  Imputatio n by  chained equations | No | Yes, COVID-AID |

| Halalau et al., | Hospital admission and in-hospital mortality | In- hospital |  | Multivariate logistic regression | No | No | Yes |
| --- | --- | --- | --- | --- | --- | --- | --- |
| He et al., | Death | In- hospital | Yes |  | No | No | No |
| He et al., |  |  | Yes | Firth logistic regression analysis | No | No | No |
| He et al., | 1. All-cause mortality; 2. ICU admission; (3) composite of   invasive mechanical ventilation or ECMO; (4)  composite of ARDS and respiratory failure | In- hospital | Yes | XGB, penalized logistic regression with Lasso or Ridge loss, random forest, decision tree, and LightGBM | Yes | Yes | No |

| Heber et al., | Mortality | In- hospital | Yes, it uses the data of the day of admission and the four subsequent  days | Logistic Regression Based Prediction Model and Benchmarking Using Machine Learning | No | Yes | Yes, with age on admission, lactate dehydrogenase, platelet count, C-reactive protein, presence of fever, and creatinine |
| --- | --- | --- | --- | --- | --- | --- | --- |
| Heldt et al., | Admission to intensive care, need for invasive mechanical ventilation and in-hospital mortality | In- hospital | Yes | Multivariate logistic regression, random forest and extreme gradient boosted trees. | Yes | Yes | No |
| Heller et al., | Mortality | Not clear | No | Not clear | No | No | No |
| Her et al., | Mortality | In- hospital | Yes | LASSO | Yes (random forest) | Yes | Yes |
| Hohl et al., | In-hospital mortality | The authors followed patients for 30 days if they were discharge  d from | Yes, all candidate predictor variables were recorded in the emergency department record | Logistic Regression Model | Yes | No | Yes, CCEDRRN COVID-19 Mortality Score |

|  |  | the emergenc y departme nt or until hospital discharge if their stay lasted longer than 30 days |  |  |  |  |  |
| --- | --- | --- | --- | --- | --- | --- | --- |
| Hu et al., | Mortality | In- hospital | Yes | Logistic regression, partial least squares (PLS) regression, elastic net (EN) model, random forest and bagged flexible discriminant analysis (FDA) | Yes | Yes | Yes, available at: https://phenomics.fudan.edu.cn/risk_score s/ |
| Ikemura et al., | Mortality | Patients were followed for 30 days or until death | Yes | H2O.ai autoML package trains and cross-validates common machine learning algorithms, such  as gradient boosting machine (GBM), extreme gradient boosting (XGBoost), general linear models (GLMs), random forest (RF), and deep learning (DL) | No | Yes | No |
| Incerti et al., | Mortality | In- hospital | Admission and from hospitalization | LASSO | Yes | Yes | No |

| Incerti et al., | Mortality | In-patient | Not clear |  | Multivariable logistic regression | Yes, Multivaria te imputatio n by chained  equation | No | No |
| --- | --- | --- | --- | --- | --- | --- | --- | --- |
| Ismail et al., | Mortality | In- hospital | Also, but obtained within 24 hours of admission to  the ICU. |  | Univariate, multivariable logistic regression, | No | No | No, but this study used Acute Physiology and Chronic Health Evaluation II (APACHE II) |
| Ji et al., | Progression of illness, days to progression, mortality, discharges, and length of  hospital stay | In- hospital | Yes | Univariate and  multivariate COX proportional hazards mode | | No | No | Yes, CALL |
| Jimenez- Solem et al., | Hospital and ICU  admission, use of mechanical ventilation and death | In- hospital | At time of SARS-CoV- 2 positivity (all patients, Diagnosis model)  The first 12 h of hospital admission (Admission model) 12 h up to ICU admission (Pre-ICU model)  12 h after ICU admission (Post-ICU model). | | Random forests | Yes | Yes (random forests) | No |

| Jiwa et al., | Any-cause mortality | In- hospital | Yes | Logistic regression | No | No | Yes |
| --- | --- | --- | --- | --- | --- | --- | --- |
| Kabootari et al., | Mortality | In- hospital | Yes | Multivariate pand univariable logistic regression, bootstrap | No | No | Yes |
| Kamran et al., | Mortality or any of three treatments indicating severe illness: mechanical ventilation, heated high flow nasal cannula, or intravenous  vasopressors. | In- hospital | Yes | Machine learning, logistic regression model | No | Yes | No |
| Kapoor et al., | COVID-19  disease severity, in- hospital mortality, and pulmonar  embolism risk | In- hospital |  | Logistic regression | NA | No | No |

| Kar et al., | Mortality | 7 and 28 days after admissio n | Yes | Logistic regression, random forest models, and eXtreme gradient boosting (XGB) algorithm | No | Yes | Yes |
| --- | --- | --- | --- | --- | --- | --- | --- |
| Karthikeya n et al., | mortality | In- hospital | Yes and every day until outcome | Neural networks, logistic regression, XGBoost, random forests, SVM, and decision trees | Yes | Yes | No |
| Katkat et al., | Mortality | In- hospital | Not clear | Univariate, backward-LR  stepwise multivariable cox regression analyses, multivariable regression models and Delong test | No | No | No, in this study various scores were validate |
| Kazemi et al., | Mortality | In- hospital |  | Multivariate logistic regression | No | No | Yes (authors created a CT score not based on the data) |
| Kim et al., | Mortality | In- hospital | Yes | Consensus | No | No | Yes |
| Kivrak et al., | Mortality | In- hospital | Yes | Deep learning, random forest, k-nearest neighbor and extreme gradient boosting | Yes | Yes | No |

| Ko et al., | Mortality | In- hospital | Yes | EDRnet (ensemble learning model based on deep neural network and random forest models) | Yes | Yes | Yes, BeatCOVID19 |
| --- | --- | --- | --- | --- | --- | --- | --- |
| Kundi et al., | Mortality | In- hospital | No | Multivariable logistic regression models | No | No | No |
| Leoni et al., | 28-day mortality | 28-day | Yes (ICU  admission) | Multivariable Cox regression | No | No | No |
| Levy et al., | Mortality | 7 days |  | LASSO logistic regression | Yes, imputatio n of means.  Variables with  >50%  missing values were excluded. | No | Yes, NOCOS Calculator |
| Li et al., | In-hospital mortality | In- hospital | Yes | multivariate Cox regression model | Yes | No | Yes |

| Li et al., | In-hospital death | In- hospital | Yes | Fine-Gray models | No | No | Yes |
| --- | --- | --- | --- | --- | --- | --- | --- |
| Liang et al., | Critical illness | In- hospital | Yes | LASSO and neural network | Yes | Yes (neural network) | Yes |
|  |  |  |  |  | Yes (if |  |  |
|  |  |  |  |  | <20%). |  |  |
|  |  |  |  |  | Predictive |  |  |
|  |  |  |  |  | mean |  |  |
|  |  |  |  |  | matching |  |  |
|  |  |  |  |  | to impute |  |  |
| Liang et al., | Composite of ICU  admission, need of invasive mechanical ventilation or death | In- hospital |  | LASSO logistic regression | numeric features, logistic regression to impute binary variables,  and Bayesian | No | Yes, COVID-GRAM |
|  |  |  |  |  | polytomo |  |  |
|  |  |  |  |  | us |  |  |
|  |  |  |  |  | regression |  |  |
|  |  |  |  |  | to impute |  |  |
|  |  |  |  |  | factor |  |  |
|  |  |  |  |  | features |  |  |

| Lin et al., | Mortality | In- hospital | Yes | Artificial neural network (ANN) and convolutional neural network (CNN) | No | Yes (Artificial neural network (ANN) and convolutional neural network (CNN) | No |
| --- | --- | --- | --- | --- | --- | --- | --- |
| Liu et al., | In‐hospital death | In- hospital | Yes | Multivariate regression analyses and Kaplan–Meier survival analysis | No | No | No |
| Liu et al., | Death | In- hospital | Yes | Logistic regression | No | No | No |
| Liu et al., | Mortality | In- hospital | Yes | Logistic regression model | No | No | Yes |
| Liu et al., | Mortality | In- hospital | Yes | Linear regression analyses | No | No | No |

| Llanera et al., | Death by day 7 of admission | In- hospital | Also | Univariate and multivariate logistic regression | No | No | No |
| --- | --- | --- | --- | --- | --- | --- | --- |
| Lopez- Escobar et al., | Mortality | In- hospital | Yes | Logistic regression | No | No | No |
| Lorente et al., | Mortality | In- hospital | Yes | Logistic | No | No | No |
| Lu et al., | Mortality | 28-days after admissio n | Day 1, 3, 7,  14 and 28 since admission | Multivariate Cox proportional hazards regression model | No | No | No |
| Lu et al., | Mortality | 12 days |  | Cox regression analysis | No | No | Yes |
| Luo et al., | Mortality | In- hospital | Yes | Univariate and multivariable logistic regression analyses | Yes | No | No |

| Ma et al., | Survival | In- hospital | Yes | Log-binomial model | No | No | Yes |
| --- | --- | --- | --- | --- | --- | --- | --- |
| Ma et al | Mortality | Not clear | Yes | Logistic regression, Random Forest and XGBoost | No | Yes (Random Forest and XGBoost) | No |
| Machado- Alba et al., | ICU admission and with mortality | In- hospital | Yes, some variables | Binary logistic regression | No | No | No |
| Magro et al., | In-hospital mortality | In- hospital | Yes | Fine and Gray proportional sub-distribution hazard model | Yes | No | Yes |
| Mahdavi et al., | Mortality | In- hospital | Yes | Support Vector Machine (SVM) | Yes | Yes | No |
| Mancilla-  Galindo et al., | Mortality | In- hospital | Also | Univariate and multivariable Cox regression model | No | No | Yes, PH-Covid19 |

| Mann et al., | In-hospital mortality | In- hospital | Yes | Logistic regression | No | No | Yes |
| --- | --- | --- | --- | --- | --- | --- | --- |
| Manocha et al., | 30-day mortality | In- hospital | Yes (Rx admission + follow up) | Logistic regression and bootstrapping | No | No | Yes |
| Marcolino et al., | Mortality | In- hospital | Yes | Logistic regression | Yes,multi ple imputatio n with chained equations  (MICE) | No | Yes, ABC2SPH |
| Martínez- Lacalzada et al., | Death, mechanical ventilation or admission to the intensive  care unit (ICU) | In- hospital | Yes | Logistic regression and least absolute shrinkage and selection operator (LASSO) | Yes, single imputatio n | No | No |
| Mayneris- Perxachs et al., | Mortality due to COVID-19  and admission to an ICU. | In- hospital and X | Also, in all cases,  glycemia and other analytes were measured using  routine laboratory  analyses in fasting | Logistic  regression analyses and machine learning (ML) algorithms | No | Yes | Yes |

|  |  |  | conditions within 24 hours of hospital admission |  |  |  |  |
| --- | --- | --- | --- | --- | --- | --- | --- |
| Mei et al., | All-cause mortality | 60 days after COVID- 19  diagnose | Yes | Multivariate logistic regression model | Yes | No | Yes |
| Mei et al., | Mortality | In- hospital | Yes | Cox proportional hazards regression analysis, LASSO | No | No | Yes |
| Mendizabal et al., | Mortality | In- hospital | Admission and from hospitalization | Logistic regression | No | No | No |
| Momeni- Boroujeni et al., | Mortality | In- hospital |  | Markov model and Regression Logistic | No | No | No |
| Monterde et al., | Critical illness in hospitalized COVID-19  patients was a composite that included the | Inhospital | Yes | Logistic regression models | No | No | No |

|  | need for invasive mechanical ventilation, transfer to the intensive  care unit (ICU), or in- hospital death |  |  |  |  |  |  |
| --- | --- | --- | --- | --- | --- | --- | --- |
| Moulaei et al., | Mortality | In- hospital | At admission or during hospitalization | ML algorithms including the J48 decision tree, random forest (RF), k-nearest  neighborhood (k-NN), multi-layer perceptron (MLP), Naïve Bayes (NB), eXtreme gradient boosting (XGBoost),  and logistic regression (LR) | No | Yes | No |
| Murri et al., | Case-fatality risk | In- hospital | Also, beyong during hospitalization | Machine learning, SHapley Additive exPlanations | No | Yes | Yes |
| Nascimento et al., | Mortality | In- hospital | Yes | Logistic | No | No | No |
| Neant et al., | Mortality | In- hospital | Admission and hospital stay | COX | No | No | No |

| Nguyen et al., | Composite ventilation, mortality | in- hospital | Yes | Logistic regression, XGBoost, and Gaussian process classifier | Yes | Yes | No |
| --- | --- | --- | --- | --- | --- | --- | --- |
| Nicholson et al., | Need of mechanical ventilation and in-hospital mortality | In- hospital | Not clear | Multivariate logistic regression | No | No | Yes: one to predict ventilation need (VICE score) and another one for death (DICE score) |
| Núñez-Gil et al., | Mortality | Not clear | Not clear | Univariate and mixed-logistic regression models | No | No | Yes, mortality risk score calculator |
| Obremska et al., | Mortality | In- hospital | Also, the majority variables were collected on admission, but also was collected in the mode of discharge. | Classifcation and regression tree (CART) | No | Yes | Yes, COVID-19 score |
| Oh & Song | Mortality | In- hospital | Yes | Logistic | No | No | No |

| Oualim et al., | In-hospital mortality | In- hospital | Yes | Kaplan-Meier survival analysi | No | No | No |
| --- | --- | --- | --- | --- | --- | --- | --- |
| Pan et al., | Death | In- hospital | Yes (ICU  admission) | SHAP algorithm, adaptive boosting (AdaBoost), gradient boosting decision tree (GBDT), eXtreme Gradient Boosting (XGBoost), and CatBoost | Yes | Yes | No |
| Peiró et al | 30-day all- cause death | 30-day | Yes | Multivariable Cox regressions | No | No | No |
| Philippe et al., | In-hospital mortality | In- hospital | Yes | Logistic regression, Kruskal–Wallis and Cochran– Armitage tests | No | No | No |
| Pigoga et al., | Mortality | In- hospital | Not clear | LASSO | yes (MICE) | Yes | Yes (Sex, age, comorbities, GCS, SBP. RR and SpO2) |
| Pimentel et al., | The start of continuous positive airway pressure (CPAP) or  non-invasive | In- hospital | Yes | Not clear | Yes | No | Yes |

|  | positive pressure ventilation (NIPPV/NIV),  admission to ICU or death |  |  |  |  |  |  |
| --- | --- | --- | --- | --- | --- | --- | --- |
| Plečko et al., | In-hospital mortality | In- hospital | ICU  admission | Logistic multivariable model with a LASSO penalty | Yes | No | Yes |
| Ponce et al., | Death | In- hospital | Also but not only | Gradient boosting decision trees (XGBoost), random forest, and an Elastic Net | Yes, k- nearest neighbour s (KNN) | Yes | Yes, AKI-COV score |
| Qeadan et al., | Mortality | Data colection | Admission and from hospitalization | Logistic regression | No | No | No |
| Qin et al., | Mortality | In- hospital | Yes | Logistic regression model | No | No | No |
| Qin et al., | Mortality | 28-day | Yes | Mixed-effects Cox model | No | No | No |

| Romualdo et al., | Mortality | In- hospital | Yes | Binary logistic regression analysis | No | No | No |
| --- | --- | --- | --- | --- | --- | --- | --- |
| Rothschild et al., | Mortality | Not clear | Yes | Nonadjusted and adjusted Cox proportional hazards models | No | No | No |
| Ryan et al., | Mortality | In- hospital | Yes | XGBoost | Yes | Yes | No |
| Saldi et al., | Mortality | In- hospital | Yes | Bivariate  and multivariate cox regression analyses | No | No | Yes, COVID-19 mortality score |
| Sankaranar ayanan et al., | Death | Within 21 days of the first positive PCR test  result | Yes, Some during hospitalization (max and min) | Traditional ML using the AutoGluon-Tabular framework and state-of-the-art techniques using recurrent neural networks utilized the TensorFlow Keras framework. | Yes | Yes | No |

| Sauzay et al., | Mortality | In- hospital | Not clear | Univariate and multivariate analyses were performed using a step-by-step backward Cox regression | No | No | Yes (Na; K; and Protrombin) |
| --- | --- | --- | --- | --- | --- | --- | --- |
| Schlauch et al., | “Discharged alive” or “death” | In- hospital | Not clear | Univariable logistic regression or multivariable logistic regression with elastic net (i.e. RTRM) | No | No | Yes, granular daily risk score |
| Schöning et al., | Severe clinical outcomes | Not clear | Up to 3 days before, or 1 day after positive testing | Logistic regression model | Yes | Yes (decision tree induction, regression trees (CART),  random forest, k- nearest neighbor (kNN),  support vector machines (SVM),  multilayer perceptrons (MLP)) | Yes |
| Sensusiati et al., | Mortality | In- hospital | Not clear | Logistic | No | No | Yes (age, NLR score, RALE score) |

| Shah et al., | Hospitalisation within the next 14 days and death within 28 days of  testing positive | Along of 4-week | Not clear | Cox regression model | No | No | No |
| --- | --- | --- | --- | --- | --- | --- | --- |
|  | Clinical |  |  |  |  |  |  |
|  | deterioration |  |  |  |  |  |  |
|  | (intubation, |  |  |  |  |  |  |
| Shamout et al., | admission to the intensive  care unit | 24, 48,  72, and 96 h | Not clear | Logistic regression and ML models (GMIC and GBM) | Yes | Yes (GMIC  and GBM) | No |
|  | (ICU), and in- |  |  |  |  |  |  |
|  | hospital |  |  |  |  |  |  |
|  | mortality) |  |  |  |  |  |  |
| Shang et al., | Mortality | In- hospital | Yes | Cox | No | No | No |
|  |  |  |  |  | Yes, |  |  |
|  |  |  |  |  | multiple |  |  |
|  |  |  |  |  | imputatio |  |  |
|  |  |  |  |  | n methods |  |  |
| Shang et al., | Mortality | In- hospital |  | LASSO logistic regression | for variables | No | Yes, CSS score |
|  |  |  |  |  | with |  |  |
|  |  |  |  |  | <10% |  |  |
|  |  |  |  |  | missing |  |  |
|  |  |  |  |  | values |  |  |

| Shao et al., | Mortality, MV, ECMO, high flow O2, low flow O2, no O2 and discharged | In- hospital | Admission and hospital stay | Logistic | No | No | Yes (ALT, CRP and LDH) |
| --- | --- | --- | --- | --- | --- | --- | --- |
| Shayganfar et al., | Mortality/ICU | In- hospital | Yes | Logistic | No | No | No |
| Singh et al., | Ventilator use and mortality | In- hospital | Variables obtained during the patient’s initial encounter up to 72 hours Following evaluation | 1. XGsBoost. 2. Random Forest Regression. 3. Logistic Regression depicts performance of the machine learning models with 10 markers selected   using the  corresponding feature selection method.   1. Deep profiler using 10 markers. | Yes | Yes | Yes |
| Sinkovits et al., | Mortality | In- hospital | Admission and from hospitalization | Cox | No | No | No |
| Sîrbu et al., | Discharged or deceased | In- hospital | Yes | Logistic regression (LR), decision trees (DT), random forests (RF), naive bayes (NB) and support vector machines (SVM) | Yes, nearest- neighbour approach | Yes | No |

| Soto-Mota et al., | Area under the curve (AUC) of each COVID-19  mortality prediction method | In- hospital | Yes |  | Yes | No | No (they tested existing scores) |
| --- | --- | --- | --- | --- | --- | --- | --- |
| Soto-Mota et al., | Mortality | In- hospital |  | Consensus | No | No | Yes (LOW-HARM) |
| Sottile et al., | Mortality | In- hospital |  | Logistic regression models, estimating the stacked model, and evaluating the  stacked mode | Yes | No | Yes, novel real-time mortality score for the COVID-19 pandemic |
| Sottile et al., | Mortality | In- hospital | Yes | Logistic regression, stacked model, machine learning. | Yes, MICE | Yes | Yes |
| Sourij et al., | Mortality | In- hospital | Yes | Univariate and multivariate logistic regression | No | No | Yes |

| Stachel et al., | Death during admission in inpatients with COVID-19  confirmed by PCR within prior 60 days  of visit | In- hospital | Yes | AI techniques - logistic regression (LR), decision tree (DT),  gradient boosting decision trees (GB), support vector machine (SVM) and neural network (NN). | Yes | Yes - tested different AI techniques to choose the one with best performance | No |
| --- | --- | --- | --- | --- | --- | --- | --- |
| Sun et al., | Mortality | Time to outcome (0, 3,  6,9,12  days earlier) | No (min, max) | Cox, k-NN, SVM, DT, BPNN, PNN, RNN, LSTM  and T-LSTM | No | Yes | No |
| Surme et al., | Mortality | In- hospital | Admission and hospital stay | Logistic | No | No | No |
| Synolaki et al., | Mortality | Not clear | Admission and hospital stay | Logistic | Yes | No | Yes (follistatin; activin A; activin B; CRP; LDH; ICU admission; NLR; Age; comorbities; d dimer) |
| Thomson et al., | ICU survival | 28 days’ follow-up | Yes (ICU  admission) | Multivariable logistic regression | No | No | No |

| Turrini et al., | Mortality | In- hospital | COX univariate and multivariate logistic regression | | No | No | Yes, Prognostic score model |
| --- | --- | --- | --- | --- | --- | --- | --- |
| Vagliano et al., | Mortality | In- hospital | Yes, at admission and after 24 hours | Machine Learning and Regression Logistic | Yes | Yes | No |
| Van Dam et al., | Mortality and admission to intensive care unit | In- hospital |  | Logistic regression | Yes, multiple imputatio n | No | Yes, Risk Stratification in the Emergency Department in Acutely ill Older Patients (RISE UP) score |
| Varol et al., | Mortality | Not clear | Yes | Logistic regression | No | No | Yes |
| Vela et al., | Hospital admission, transfer to intensive care unit (ICU), and death. | In- hospital |  | Generalized linear models (Poisson regression) | No | No | No |
| Velasco-  Rodriguez et al., | Mortality | In- hospital | Admission  and trought hospital stay | Logistic regression | Yes | No | No |

| Vepa et al., | Inpatient mortality, required ventilatory support, and duration of  inpatient treatment | In- hospital | Not clear | Bayesian Network Modelling | Yes | Yes | No |
| --- | --- | --- | --- | --- | --- | --- | --- |
| Verma et al., | Mortality, unplanned readmission to any medical service or medical– surgical ICU service at any participating hospital within 30 days of discharge, admission to the ICU, total hospital length of stay and  ICU length of stay | In- hospital | Yes | Multivariable regression | Yes | No | Yes |
| Vicka et al., | Mortality | In- hospital | Also (Upon admission to the hospital, the 4C Mortality Score  was calculated.  Upon admission to | Univariate regression analysis, multivariate regression analysis | No | No | No, in this study various scores were validate |

|  |  |  | the ICU, the APACHE II, SAPS II  and SOFA scores were implemented) |  |  |  |  |
| --- | --- | --- | --- | --- | --- | --- | --- |
| Vila et al., | Mortality | In- hospital | Admission and hospital stay | COX | No | No | No |
| Wang et al., | In-hospital mortality | In- hospital | Yes | Multivariate logistic regression | No | No | No |
| Wang et al., | Severe COVID or death | In- hospital | Yes | Logistic regression analysis | No | No | No |
| Wang et al., | Mortality | In- hospital | Not only | Logistic regression analysis and XGBoost model | No | Yes (XGBoost) | No |
| Wang et al., | Mortality | In- hospital | Admission  and in hospital stay | GLM | No | No | No |

| Wang et al., | Deceased, ventilated, or admitted to the intensive care unit (ICU) | In- hospital | In part | XGBoost and Logistic regression | Yes | Yes (XGBoost) | No |
| --- | --- | --- | --- | --- | --- | --- | --- |
| Wang et al., | Mortality | 28 days |  | Multivariable logistic regression | No | No | Yes (FAD-85) |
| Wang et al., | Death (non- survivor) and discharge or hospitalization (survivor) | In- hospital | Not clear | Deep learning and radiomics, and logistic regression | No | Yes | No |
| Weng et al., | Mortality | In- hospital |  | LASSO logistic regression | Yes, for variables with  <10%  missing values (>10%  were excluded from model developm  ent). RF. | No | Yes (ANDC) |

|  | Hospitalization |  |  |  |  |  |  |
| --- | --- | --- | --- | --- | --- | --- | --- |
|  | with |  |  |  |  |  |  |
|  | pneumonia, |  |  |  |  |  |  |
|  | hospitalization |  |  |  |  |  |  |
|  | with | In- |  |  |  |  |  |
| Williams et al., | pneumonia  requiring intensive | hospital  and 30 days after |  | LASSO logistic regression | No | No | Yes, 3 scores (COVER-F for death) |
|  | services or | index rate |  |  |  |  |  |
|  | death and |  |  |  |  |  |  |
|  | death in the 30 |  |  |  |  |  |  |
|  | days after |  |  |  |  |  |  |
|  | index date |  |  |  |  |  |  |
| Wongvibuls in et al., | Mortality and severe illness | 7 days | Admission and hospital stay | RF SLAM | Yes | Yes | Yes |
| Wu et al., | Mortality | Not clear | Not clear | Cox regression analysis | No | No | Yes |
| Xie et al., | Mortality | In- hospital |  | Multivariate logistic regression | No | No | Yes |

| Yan et al., | Mortality | In- hospital | No | Cox regression models | No | No | No |
| --- | --- | --- | --- | --- | --- | --- | --- |
| Yasukawaet al., | Mortality | In- hospital | Yes | Logistic | No | No | No |
| Yavuz et al., | Mortality | In- hospital | Admission and from hospitalization | Logistic regression | No | No | No |
| Yoo et al., | Mortality | In- hospital | Yes | Gray`s K-sample tests, DeLong's test | No | No | Yes |
| Yu et al., | In-hospital death | In- hospital | Yes | Logistic regression | No | No | Yes |
| Yu et al., | Mortality | Not clear | Not clear | Catboost | Yes | Yes | No |

| Zayat et al., | In-hospital mortality | In- hospital | Yes | Multivariate parametric survival regression analysis | No | No | No |
| --- | --- | --- | --- | --- | --- | --- | --- |
| Zeng et al., | Disease deterioration | In- hospital | Not clear | Logistic regression And LASSO regression | No | LASSO | ys |
| Zhang et al., | Mortality | From hospital admissio n to May 2, 2020 | No | Cox proportional HR models | No | No | No |
| Zhang et al., | Mortality | 14 days  and 28 days | Yes | Cox regression analyses | Yes. Multiple imputatio ns (method not  reported) | No | Yes |
| Zhang et al., | Death and poor outcome (developing ARDS,  receiving intubation ou ECMO  treatment, ICU admission or death) | In- hospital | Yes | LASSO logistic regression | No | No | Yes, DCS, DCSL, DL |

| Zhao et al., | Intensive care unit (ICU) admission and mortality | In- hospital | Not clear | Logistic regression | No | No | Yes, risk scores for mortality |
| --- | --- | --- | --- | --- | --- | --- | --- |
| Zhou et al., | Mortality | In- hospital | Yes | Univariable and multivariable logistic regression methods | No | No | No |
| Zhou et al., | Mortality | In- hospital | Yes | Multivariable logistic regression | No | No | Yes, NLAUD |
| Zhu et al., | Mortality | In- hospital | Admission and from hospitalization | COX | No | No | No |
| Zinellu et al., | Mortality | In- hospital | Yes | Cox proportional hazards regression | No | No | No |

**Table S1. Continued**

| **Study** | **Number of variables were tested in the development cohort** | **Univariate analysis** | **How many patients died in the derivation**  **dataset?** | **External validation** | **AUC in derivation cohort** | **AUC in validation cohort** | **F1 score** | **TRIPOD** |
| --- | --- | --- | --- | --- | --- | --- | --- | --- |
| Abbasi et al., | 35 | No | 56 | No | 0.839 (standard error  0.03; CI, 0.78–0.9) | NA | No | No |
| Abdollahpour et al., | Not clear | Yes | Not disclosed | No | 0,989 | Not disclosed | No | No |
| Abdulaal et al., | 22 | Yes | 17 | Yes | Cross-validated (on the training and validation set) 90.12% | Cross-validated (on the training and validation set) 90.12% | No | No |
| Acar et al., | Not clear | Yes | 75 | No | 0,922 | Na | No | No |
| Adderley el at., | Not clear (~67) | No | Derivation: 288; External validation: 1668, | Yes | UHB: 0.779 (0.744 to  0.813);  UHB-R†: 0.791 (0.761  to 0.822) | CovidCollab 0.767  (0.754 to 0.780);  UHB—4C score: 0.753 (0.720 to 0.785) | No | No |
| Agarwal et al., | 10 | Yes | 51 | No | Not clear | NA | Yes | No |
| Ageno et al., | Not clear (~15) | No | 181 | No | 0.78 | 0.66 | No | No |
| Akdur et al., | NA (they tested existing scores) | Yes | 128 | Yes (they validated existing scores) | Base model (Age, gender, PPI and SI) alone: 14-day mortality  > 0.938 (0.912–0.963),  90-day mortality > 0.951 (0.931–0.972);  Base model + CT score:14-day mortality > | NA | No | No |

0.968 (0.956–0.981);

90-day mortality: 0.987

(0.981–0.993). NEWS

score alone:14-day mortality: > 0.908

(0.871–0.944), 90-day

mortality > 0.907

(0.871–0.943); NEWS

+CTSS: 14-day

mortality: 0.955 (0.940–

0.970) and 90-day

mortality: 0.955 (0.940– 0.970); Base model + NEWS: 14-day

mortality: 0.963 (0.949–

0.977); 90-day

mortality: 0.977 (0.966– 0.988); Base model + NEWS + CTSS: 14-day: 0.973 (0.962–0.984);

90-day: 0.991 (0.986–

0.996). Quick sofa

alone: 14-day: 0.829

(0.779–0.879); 90-day:

0.835 (0.793–0.876).

Quick sofa + CTSS: 14- day:0.949 (0.927–

0.971); 90-day: 0.967

(0.950–0.984). Base

model + Qsofa: 14-day: 0.961 (0.947–0.975),

90-day: 0.977 (0.966–

0.987). Qsofa+base model+ CTSS: 14-day: 0.972 (0.961–0.984);

|  |  |  |  |  | 90-day: 0.991 (0.986–  0.996) |  |  |  |
| --- | --- | --- | --- | --- | --- | --- | --- | --- |
| Ali et al., | NA | NA | 117 | NA | NA | NA | No | No |
|  |  |  |  |  | Prognostic model |  |  |  |
|  |  |  |  |  | Without PRO-AGEa |  |  |  |
|  |  |  |  |  | With PRO-AGEa |  |  |  |
|  |  |  |  |  | Base model = age, sex |  |  |  |
|  |  |  |  |  | and Charlson |  |  |  |
|  |  |  |  |  | comorbidity score 30- |  |  |  |
|  |  |  |  |  | day mortality 0.66 |  |  |  |
|  |  |  |  |  | (0.64–0.69) 0.74 (0.71– |  |  |  |
|  |  |  |  |  | 0.76) |  |  |  |
|  |  |  |  |  | 60-day mortality 0.66 |  |  |  |
|  |  |  |  |  | (0.63–0.69) 0.73 (0.71– |  |  |  |
|  |  |  |  |  | 0.76) |  |  |  |
| Aliberti et al., | 17 | No | 605 | No | National Early Warning Score (NEWS) 30-day | NA | No | No |
|  |  |  |  |  | mortality 0.71 (0.68– |  |  |  |
|  |  |  |  |  | 0.74) 0.75 (0.73–0.78) |  |  |  |
|  |  |  |  |  | 60-day mortality 0.71 |  |  |  |
|  |  |  |  |  | (0.68–0.73) 0.75 (0.73– |  |  |  |
|  |  |  |  |  | 0.78) |  |  |  |
|  |  |  |  |  | Base model + NEWS |  |  |  |
|  |  |  |  |  | 30-day mortality 0.77 |  |  |  |
|  |  |  |  |  | (0.74–0.79) 0.79 (0.76– |  |  |  |
|  |  |  |  |  | 0.81) |  |  |  |
|  |  |  |  |  | 60-day mortality 0.77 |  |  |  |
|  |  |  |  |  | (0.74–0.79) 0.79 (0.76– |  |  |  |
|  |  |  |  |  | 0.81) |  |  |  |
| Allenbach et al., | 42 | Yes | 32 | Yes | 0.786 for the composite outcome and 0.803 for death (after correction for over-optimism; IC95% not reported) | 0.787 for the composite outcome and 0.827 for death (after correction for over-optimism;  IC95% not reported) | No | Yes |

| Altini et al., | 76 (Specifically, demographic data included variables, such as age and sex, the clinical characteristics examined were date of hospitalization, record the date of transfer to  ICU, date of discharge from all COVID units including the ICU, date of death, days of hospitalization; as for laboratory tests, a total of 69 hematochemical  parameters were analyzed) | No | 85 | No | Decision tree: 89,66 Gaussian Naive Bayes: 88,14  Support Vector Machine: 86,86  K-Nearest Neighbor: 77,80  Random Forest: 88,30  Adaboost: 89,10 | Not clear | No | No |
| --- | --- | --- | --- | --- | --- | --- | --- | --- |
| Altschul et al., | Not clear | Yes | 621 | Yes | 0.824 (0.814 to 0.851) | 0.798 (0.789 to 0.818) | No | No |
| Avendaño‐Ortiz et al., | 53 | Yes | 14 | No | The score obtained from this model showed an  AUC/ROC of 0.9753  for mortality prediction | Not clear | No | No |
| Aznar-Gimeno et al., | 165 | No | Not clear as it is a composite outcome (ICU admision + mortality) | Yes | 20-variable model: AUC = 0.8153; 95% CI  0.7655–0.8615 | AUC = 0.821; 95% CI  0.787–0.854 | No | No |
| Bai et al., | More than 300 clinical features | No | 140 | Yes | 85% | Yes | No | No |
| Banoei et al., | 108 | Yes | 31 | No | AUC=0.95 | AUC = 0.91 | No | No |
| Baqui et al., | 30 | Yes | 87223 | No | 0.813 (95% CI 0.810–  0.817) | Not clear | No | Yes |
| Bartoszko et al., | 61 | Yes | 53 | No | 0.9 (95% confidence interval [CI], 0.8 to 0.9). | Not clear | No | Yes |

| Bello-Chavolla  et al., | 12 | No | 4276 | Yes | 0.823 (95% CI not  reported) | 0.830 (95% CI not  reported) | No | No |
| --- | --- | --- | --- | --- | --- | --- | --- | --- |
| Bello-Chavolla et al., | 19 | Yes | 317 | Yes (they externally validated an existing score, but the novel score they produced was  not externally validated) | Nutri-COVID: c-  statistic = 0.797, 95%CI 0.765–0.826 | MSL-COVID  (external validation of an existing score): c- statistic = 0.722,  95%CI 0.690–0.753 /  Nutri-COVID (internal validation): 0.772, 95%CI  0.0.745–0.800 | No | Yes |
| Bennouar et al., | NA | Yes | 37 | No | Vit D 25-OH-  hydroxyvitaminD: 0.73  [0.63–0.82]; Serum  Calcium: 0.77[0.68–  0.85] | NA | No | No |
| Bennouar et al., | Not clear | Yes | 44 | No | 0,84 (mortality) | 0,90 (mortality) | No | No |
| Berenguer et al., | 17 | Yes | 849 | Yes | 0,822 | 0,845 | No | Yes |
| Berry et al., | 22 | Yes | 336 | No | NA | NA | No | Yes |
| Bertsimas et al., | 22 | No | 760 | Yes | AUC of 0.90 (95% CI,  0.87–0.94) | Greek cohort: 0.87  (95% CI, 0.84–0.91) /  United States cohort: | No | No |
| Boudou et al., | Not clear (~31) | No | 1326 | No | AUC de 0,955 (95% CI  0,951 0,959) | No | No | No |
| Brieghel et al., | Not disclosed | Yes | 76 | No | Not disclosed | NA | No | No |
| Cai et al., | Not clear (they were interested in testing just one parameter: neutrophil-  to-lymphocyte ratio) | Yes | 399 | No | (AUROC) value of 0.89 (95% CI, 0.87–0.91) | AUROC of 0.86 (95% CI,  0.84–0.88) | No | No |
| Castro VM,  McCoy TH, Perlis RH | Not clear | No | 167 | No | 0.83 (95% CI, 0.80-  0.87) | No | No | Yes |

| Chen et al., | 37 | No | 50 | No | 0.91 (95% CI, 0.85-  0.97) | NA | No | No |
| --- | --- | --- | --- | --- | --- | --- | --- | --- |
| Cheng et al., | 50 | Yes | 85 | No | D-dimer 0.88 (0.83–  0.92) / BUN 0.88 (0.83–  0.93) / CRP 0.87 (0.83–  0.91) / CRP+BUN 0.92 (0.89-0.96) / D-  Dimer+BUN 0.94 (0.90-  0.97) / The C-index for the prediction  nomogram was 0.94  (95% CI 0.90–0.97). | Through bootstrapping validation, the bias- adjusted C-index was confirmed to be 0.929 | No | No |
| Cho et al., | Not clear | Yes | 234 | No | Not disclosed | 0,918 (14 day) 0,896  (28 day) | No | No |
| Chow et al | 13 | Yes | Not clear | Yes | Concordance statistic: 0.948, 95% confidence  interval 0.900–0.997 | Concordance statistic: 0.940, 95% confidence  interval 0.870–1.009 | No | Yes |
| Chua et al., | Not clear | Yes | 294 | Yes | 0,73 | 0,63 | No | Yes |
| Chung et al., | 37 | No | NA | No | NA (only tested the final model using deep neural networks in the validation set) | 0.96 (0.01) | No | No |
| Chuperk et al., | Not disclosed | Yes | 1537 | NA | Not disclosed | NA | No | No |
| Clift et al., | 91 | No | 4384 | No | Not clear | Not clear | No | Yes |
| Corradi et al., | Not clear | Yes | 12 | No | 0,79 | NA | No | No |
| Covino et al., | Not disclosed | Yes | 77 | No | not disclosed | NA | No | No |

| Dashti et al., | 11 | No | 509 | No | Not clear | C-statistics of 0.77  [95% CI 0.73–0.80]  for hospitalization, and 0.84 [95% CI  0.74–0.94] for  mortality | No | No |
| --- | --- | --- | --- | --- | --- | --- | --- | --- |
| Deng et al., | NA | NA | 50 | No | Ferritin (0.822, 95% CI  0.737–0.907); PCT  (0.751, 95% CI 0.654–  0.848) and CRP (0.714,  95% CI 0.609–0.819) | NA | No | No |
| Di Castelnuovo et al., | 32 | Yes | 712 | No | Not clear | NA | F1 value 90.4% | No |
| Ding et al., | Not clear | Yes | 100 | No | C-index value of 0.876 (95% CI 0.833-0.918,  model 1) and 0.887  (95% CI 0.844-0.929,  model 2). - They used C-index not AUC | NA | No | No |
| Doganci et al., | 26 | Yes | 34 | No | Not clear | NA | No | No |
| Doher et al., | Not clear | Yes | 29 | No | No (Kaplan-Meier curves) | NA | No | No |
| Dominguez- Olmedo et al., | 32 | No | 262 | No | 0.97 (0.96-0.98) | 0.86 (95% CI 0.80-  0.91) | Yes Training - 0.76 (0.71-  0.81) / Validation - 0.77 (95%  CI 0.72-  0.83) | No |

| Ebrahimi et al., | Not clear (~35) | No | 27 | No | WBC count: AUC=0.772 (95% CI:  0.719–0.820, P<0.001;)  Creatinine: AUC=0.742 (95% CI: 0.687-0.792, P<0.001) | Not clear | No | No |
| --- | --- | --- | --- | --- | --- | --- | --- | --- |
| El-Raheem et al., | 21 | No | 30 | No | NA | NA | No | No |
| Eskandar et al., | 24 | Yes | 199 | No | Did not report | NA | No | No |
| Faisal et al., | Not clear | No | 323 | Yes | CARMc19_NB = 0.87  (95% CI 0.85-0.89) vs  CARMc19_N 0.86  (95% CI 0.84-0.87) | CARMc19_NB = 0.88  vs CARMc19_N = 0.86 | No | Yes |
| Fan et al., | Not clear | Yes | 31 | Yes | 0,91 | Not disclosed | No | No |
| Fernandes et al., | 57 | Yes | 92 | No | Considering only death as an outcome: 0.972 [0.95; 1.00] - best  model: extra-trees | Same | Yes | Yes |

| Fumagalli et al., | 20 | Yes | 120 | No | 0.90 (95% CI 0.87 to  0.93) | NA | No | No |
| --- | --- | --- | --- | --- | --- | --- | --- | --- |
| Galiero et al., | Not clear | Yes | 143 | No | NA | NA | No | No |
| Galloway et al., | 19 | No | 244 | No | 0.697 (0.652,0.741) | NA | No | No |
| Garrafa et al., | 17 + an existing chest X- ray score that was incorporated to the model | Yes | 423 | Temporal validation - using patients from the second COVID-19 wave in Italy (May  - December 2020) | 0.93 | 0.82 (temporal validation second-  wave: 0.73) | No | Yes |
| Gatti et al., | Not disclosed | Yes | 83 | No | 0,86 | NA | No | No |
| Giradin et al., | Not clear | Yes | 959 | NA | 0,76 | NA | No | No |

| Goméz et al., | 20 | No | 33 | No | 0.874 (0.816-0.933) | NA | No | No |
| --- | --- | --- | --- | --- | --- | --- | --- | --- |
| Gopalan et al., | ~39, including data on clinical manifestations, comorbidities, vital signs, and basic lab investigations | Yes | 259 | No | 0.85 (95% CI: 0.81–  0.89) | Not clear | No | No |
| Gorham et al., | NA they tested IL-6 levels (potential biomarker) | No | 13 | No | AUROC 0.73 [95% CI  0.57-0.89] | NA | No | No |
| Guan et al., | 48 | Yes | 41 | Yes | 0.921 (0.038) | 0.891 (0.053) | Training: 0.949 /  Internal validation: 0.933 /  External validation: 0.923 | No |
| Gude-Sampedro et al., | Not clear | Yes | 384 | No | 0.77 (95%CI: 0.76,  0.78) | 0.77 (95%CI: 0.75,  0.79) | No | Yes |
| Gue et al., | 15 | No | 145 | No | 0.793 (95% CI 0.745–  0.841) | NA | No | No |
| Gupta et al., | 40 | Yes | 16 885 | Yes | C-statistic 0·77 [95% CI 0·76 to 0·78]); (They used C-statistics not AUC) | C-statistic 0·77 [95% CI 0·76 to 0·78]);  (They used C-statistics not AUC) | No | Yes |
| Gupta et al., | Not clear (demographics, medical history, presenting symptoms, medications, select inpatient  therapies, labs, and clinical outcomes) | Yes | 403 (derivation) +  85 (validation) | No | 0.783 (95% CI, 0.76 to  0.81) | 0.81  (95% CI, 0.78-0.84) | No | Yes |
| Hajifathalian et al., | 38 | Yes | 93 | Yes | 7 days: 0.877 (95%CI  0.831–0.923); 14 days: | 7 day (0.851 [0.781 to  0.921]); 14 day (0.825  [0.764 to 0.887]) | No | Yes |

|  |  |  |  |  | 0.847 (95%CI 0.806–  0.888) |  |  |  |
| --- | --- | --- | --- | --- | --- | --- | --- | --- |
| Halalau et al., | Not clear | No | Not clear | Yes | Not available | 0.75 (0.71 – 0.78) | No | No |
| He et al., | NA interestied in testing only d-dimer | Yes | Not clear | No | AUC D-dimer: 0.661 (first test) 0.909 (last test) | NA | No | No |
| He et al., | Not clear | Yes |  | No | Reported Kaplan–Meier survival analysis of two  biomarkers | NA | No | No |
| He et al., | 386 | No | 6204 | Yes, using the postdevelopment prospective test data set collected from September 7  to November 15,  2020, | AUC 0.88, 95% CI  0.87-0.88 on the test data set | AUC 0.84, 95% CI  0.84-0.85 on the prospective test data set | No | No |
| Heber et al., | 12 subject matter-based predictors were pre- selected to be narrowed down by the described bootstrap approach. | No | Developement: 120;  Validation: 67; External validation: 42 | Yes | Tenfold cross validation resulted in a mean area under the receiver operating characteristic curve (AUROC) of 0.92, a mean calibration slope of 1.0023 and a  Brier score of 0.076 | At temporal-external validation, application of the previously developed model showed an AUROC of 0.88, a calibration slope of 0.95 and a  Brier score of 0.073 | No | No |
| Heldt et al., | 64 | No | 193 | No | Logistic regression AUC of 0.70, Random forest: 0.77and  XGBoost 0.76 | NA | F1 scores of 0.56–0.61 | No |
| Heller et al., | 3 | No | 6 | No | AUC of 71.1% for Zn, 74.5% for Se, and 76.5% for SELENOP /  SELENOP and Zn with age 94.42%. | NA | No | No |

| Her et al., | 35 | No | 169 | No | 0.97 | 0.96 | No | No |
| --- | --- | --- | --- | --- | --- | --- | --- | --- |
| Hohl et al., | Not clear (~19: age, sex, pregnancy, type of residence, mode of arrival at the emergency department, comorbidities, symptoms, heart rate on arrival, systolic blood pressure, oxygen saturation level, respiratory rate, Glasgow Coma Scale score, oxygen delivery in the emergency department, lowest oxygen saturation level, physician or nurse impression of respiratory distress, and use of alcohol, tobacco, vaping  or illicit substances) | No | 618 | No | 0.92 (95% confidence interval [CI] 0.90–0.93) | 0.92 (95% CI 0.90–  0.93) | No | Yes |
| Hu et al., | 51 | Yes | 68 | Yes | 0,895 (using the logistic regression model) | 0,881 (using the  logistic regression model) | No | No |
| Ikemura et al., | 48 | No | 1087 | No | 0.860-0.904 (Table 3) | Not clear | No (they used F2 score) | No |
| Incerti et al., | Not disclosed | No | Not disclosed (2163 total) | No | 0,88 | 0,87 | No | No |
| Incerti et al., | 55 | Yes | 2163 | No | 0,8822 | 0,8741 | No | No |

| Ismail et al., | Demographic data, referring facility, preexisting comorbid conditions, and the initial manifestations of COVID19 prior to hospital admission were recorded.  Laboratory parameters and therapeutic interventions on admission to the ICU were retrieved electronically from the local patient data management system (Cerner corp, North Kansas City, Missouri, United States) and recorded. The Acute Physiology and Chronic Health Evaluation II (APACHE II) score was calculated from  the data obtained within 24 hours of admission to the ICU [28]. | Yes | 75 | No | APACHE II score: (AUC = 0.77; 95% CI:  0.71–0.84, p<0.001)  NLR (AUC = 0.69; 95%  CI: 0.61–0.76, p<0.001)  LDH value (AUC = 0.66; 95% CI: 0.59–  0.73, p<0.001) | No | No | No |
| --- | --- | --- | --- | --- | --- | --- | --- | --- |
| Ji et al., | Not clear | Yes | 2 | No | 0.91 (95% CI 0.86 -  0.94) | C-index: 0.86  (95%CI 0.81-0.91) | No | No |
| Jimenez-Solem et al., | 20 | No | 324 | Yes | 0.906 at diagnosis,  0.818, at hospital admission and 0.721 at Intensive Care Unit (ICU) admission / hospital admission 0.820, ICU admission  0.802, ventilator  treatment 0.815 and  death 0.9020.820, ICU  admission 0.802, | External validation: 0.661 for predicting hospital admission,  0.529 for predicting ICU admission and 0.742 for predicting  mortality | No | No |

|  |  |  |  |  | ventilator treatment 0.815 and death 0.902 |  |  |  |
| --- | --- | --- | --- | --- | --- | --- | --- | --- |
| Jiwa et al., | 10 | Yes | 36 | No | Model A (2 variables: presence/absence of ischemia and consolidation): 0.74  (95% CI 0.65 to 0.82) /  Model B ((3 variables: older age, high supplemental oxygen requirement and elevated CRP): AUC 0.66 (95% CI 0.56 to  0.77) | Model A: 0.65 (95%  CI 0.55 to 0.76) /  Model B: 0.74 (95%  CI 0.65 to 0.83) | No | No |
| Kabootari et al., | >56. Not clear | No | 165 | No | 0.75  (95% CI 0.70e0.80) for model 1, 0.80 (95% CI  0.74e0.82) for model 2, and 0.86 (95% CI  0.83e0.90) for model 3. The corrected AUC for model 3 was 0.82 (95%  CI 0.79e0.89). | Not clear | No | No |
| Kamran et al., | 2686 | No | Not clear | Yes | Not clear | Internal validation cohort: 0.80 (95% confidence interval 0.77 to 0.84) ;  External validation in 12 medical centers  (AUROC range 0.77-  0.84) | No | Yes |
| Kapoor et al., | 127 | No | 33 | NA | No | Illustrated in Figure 2. | No | No |
| Kar et al., | 23 | Yes | Not clear | Yes (temporal validation) | 0,8685 | 0,782 | No | Yes |

| Karthikeyan et al., | 74 | No | 169 | No | Neural Net: 0.9895  (0.0057) / SVM 0.9903  (0.0014) / Logistic  Regression 0.9934  (0.00015) / Random  Forests 0.9858 (0.0020)  / XGBoost 0.9838 (0.0022) / Decision Tree 0.9771 (0.0033) | Neural Net: 0.9895  (0.0057) / SVM  0.9903 (0.0014) /  Logistic Regression 0.9934 (0.00015) /  Random Forests 0.9858 (0.0020) /  XGBoost 0.9838  (0.0022) / Decision  Tree 0.9771 (0.0033) | Yes (Neural Net: ) 0.9687  (0.006) / SVM 0.9577  (0.0046) /  Logistic Regression 0.9537  (0.0018) /  Random Forests 0.9467  (0.0065) /  XGBoost 0.9435  (0.0118) /  Decision Tree ) 0.914  (0.0182) | No |
| --- | --- | --- | --- | --- | --- | --- | --- | --- |
| Katkat et al., | Not clear (~34) | Yes | 68 | No | NA | R2CHA2S2-VASc (AUC:0.76, CI 95%  0.72-0.79, p<0.001), CHA2DS2-VASc (AUC: 0.72, CI 95%  0.68-0.76, p<0.001), CHA2DS2-VASc-HS (AUC:0.72, CI 95%  0.68-0.76, <0.001) | No | No |
| Kazemi et al., | Not available | No | 11 | No | 0.73 (95% CI not reported) | NA | No | No |
| Kim et al., | Not clear | No | 7 | No | Not reported | NA | No | No |

| Kivrak et al., | 13 | Yes | Not clear | No | Did not report (Reported accuracy) | Did not report (reported accuracy) | No | No |
| --- | --- | --- | --- | --- | --- | --- | --- | --- |
| Ko et al., | 28 | Yes | 212 | Yes | Did not report (Reported sensitivity, especificity, accuracy, VPP, VPN) | Same | No | No |
| Kundi et al., | 24 | No | 697 (12.0%), 1,751  (18.2%) and 867  (31.0%) in low, intermediate and high hospital frailty risk, respectively | No | C-statistics without Hospital Frailty Risk Score/C-statistics with Hospital Frailty Risk Score  All-cause Mortality 0.70  (0.69-0.72) 0.73 (0.72-  0.74)  Long-length stay (>10  days) / 0.59 (0.58-0.60)  / 0.61 (0.60-0.61)  Intensive care unit / 0.67 (0.66-0.68) / 0.70 (0.69-  0.70)  Invasive mechanical ventilation / 0.66 (0.65-  0.68) / 0.68 (0.67-0.69) | NA | No | No |
| Leoni et al., | 26 | Yes | 85 | No | 0.821 (95% CI 0.766–  0.876) | 0.822 (95% CI 0.770–  0.873) | No | Yes |
| Levy et al., | 42 | No | Not clear | Yes | 0.86 (95% CI not reported) | 0.82 (95% CI not reported) | No | No |
| Li et al., | 48 | Yes | 47 | Yes | 0.97 (95% CI, 0.95 to  0.98, P<0.001) | Internal validation: 0.96 (95% CI, 0.94 to  0.98, P<0.001) /  External validation: 0.92 (95% CI, 0.86 to  0.98, P=9.7×10-38) | No | Yes |
| Li et al., | 12 | Yes | 211 | Yes | C-index: 0.85, 95% CI:  0.83 to 0.87 (They used | C-index: 0.87, 95%  CI: 0.85 to 0.89 (They | No | Yes |

|  |  |  |  |  | C-index instead of AUC) | used C-index instead of AUC) |  |  |
| --- | --- | --- | --- | --- | --- | --- | --- | --- |
| Liang et al., | 15 | Yes | Not clear | Yes | 0.911 (0.95 CI, 0.875–  0.945) | Internal validation: 0.889 (0.95 CI, 0.843–  0.934)  External validation: The C-index of the entire dataset for the Wuhan, Hubei, and Guangdong cohorts were 0.878, 0.769, and  0.967, respectively. In the Ex3 dataset, the C- index for these cohorts were 0.890, 0.852, and  0.967, respectively. | No | No |
| Liang et al., | 72 | No | 51 (3.2%) | Yes | 0.88 (0.85 - 0.91) | 0.88 (0.84 - 0.93) | No | No |
|  |  |  |  |  | A. Raw data | 1. Raw data CNN 0.86 / ANN 0.82   / Forest(J48) 0.64 / Random forest 0.93 / Random tree 0.63 / REPT tree 0.75 / BayesNet 0.91 / Naïve Bayes 0.91 / Logistic  0.36 / SMO 0.95   1. Normalization data   CNN 0.73 / ANN  0.80 / Forest(J48) 0.64  / Random forest 0.90 / Random tree 0.93 / REPT tree 0.85 / BayesNet 0.92 / Naïve |  |  |
|  |  |  |  |  | CNN 0.85 / ANN 0.89 / |  |  |  |
|  |  |  |  |  | Forest (J48) 0.99 / |  |  |  |
|  |  |  |  |  | Random forest 1.00 / |  |  |  |
|  |  |  |  |  | Random tree 1.00 / |  |  |  |
|  |  |  |  |  | REPT tree 0.88 / |  |  |  |
|  |  |  |  |  | BayesNet 0.90 / Naïve |  |  |  |
|  |  |  |  |  | Bayes 0.86 / Logistic |  |  |  |
| Lin et al., | 30 | No | 166 | Yes | 0.91 / SMO 0.88 |  | No | No |
|  |  |  |  |  | B. Normalized data |  |  |  |
|  |  |  |  |  | CNN 0.91 / ANN 0.96 / |  |  |  |
|  |  |  |  |  | Forest (J48) 0.97 / |  |  |  |
|  |  |  |  |  | Random forest 1.00 / |  |  |  |
|  |  |  |  |  | Random tree 1.00 / |  |  |  |
|  |  |  |  |  | REPT tree 0.91 / |  |  |  |
|  |  |  |  |  | BayesNet 0.90 / Naïve |  |  |  |

|  |  |  |  |  | Bayes 0.84 / Logistic  0.91 / SMO 0.88 | Bayes 0.92 / Logistic  0.86 / SMO 0.70 | |  |
| --- | --- | --- | --- | --- | --- | --- | --- | --- |
| Liu et al., | Not clear (focus on one biomarker) | Yes | 15 | No | AUC: 0.808, p < .001 | NA | No | No |
| Liu et al., | Not clear | Yes | 138 | No | 0.82 (95% CI 0.78 to  0.87) for IL-2R, 0.85 (95% CI 0.81 to 0.89) for IL-6, 0.69 (95% CI  0.64 to 0.75) for IL-8, 0.75 (95% CI 0.69 to 0.81) for IL-10, and 0.71 (95% CI 0.65 to  0.77) for TNF | NA | No | No |
| Liu et al., | 55 | Yes | 37 | No | 0.953 (95% CI 0.908 to  0.997) | NA | No | No |
| Liu et al., | 40 | Yes | 35 | No | AUC values for PT, FDP and DD were 0.892, 0.81 and 0.809,  respectively | NA | No | No |
| Llanera et al., | Not clear (~38) | Yes | 241 | No | No | No | No | No |
| Lopez-Escobar et al., | Not disclosed | Yes | 321 | No | Not disclosed | NA | No | No |
| Lorente et al., | Not clear | Yes | 11 | No | 0,83 | NA | No | No |
| Lu et al., | 8 | No | 112 | No | Not clear | NA | No | No |
| Lu et al., | Not clear | Yes | 39 | No | Not reported | NA | No | No |
| Luo et al., | 20 | Yes | 201 | No | AUCCD8+ = 0.832  [0.804–0.861] vs. AUC  CD3+ = 0.758 [0.726– | NA | No | No |

|  |  |  |  |  | 0.791] or AUCCD4+ =  0.721 [0.686–0.755] |  |  |  |
| --- | --- | --- | --- | --- | --- | --- | --- | --- |
| Ma et al., | 26 | Yes | 128 | No | AUC of 0.948 (95% CI:  0.923–0.973) | NA | No | No |
| Ma et al., | 33 | No | 57 | No | AUROC of 0.9521,  which was better than CURB-65 (AUROC of  0.8501) and the machine-learning-based model (AUROC of 0.4530 | AUROC of 0.6061  was also better than CURB-65 (AUROC  of 0.4608) and the machine-learning- based model (AUROC of 0.2292) | No | No |
| Machado-Alba et al., | >42 | No | 203 | No | No | No | No | No |
| Magro et al., | Not available | Yes | 495 | Yes | AUC = 0.822, 95% CI  0.722–0.922 | AUC = 0.820, 95% CI  0.724–0.920 | No | No |
| Mahdavi et al., | 57 | No |  | No | Non-invasive model:  0.85 ± 0.04 / Joint model: 0.92±0.03 /  Invasive model: 0.85 ± 0.04 | Same | No | Yes |
| Mancilla- Galindo et al., | 30 | Yes | 9228 (validation) +  5278 (validation) | No | AUC: 0.80 (95% CI  0.796–0.804) | Not clear | No | Yes |
| Mann et al., | 145 | Yes | 347 | Yes | 0.796 (95% confidence  interval, 0.767–0.826) | 0.829 (95%  confidence interval, 0.782–0.876) | No | No |
| Manocha et al., | 11 | Yes | 95 | No | 0.834 (95% CI, 0.792–  0.876) | C‐statistic was 0.784  (95% CI, 0.729–  0.838). Validation with the bootstrapping method yielded a C‐ statistic of 0.792 (95%  CI, 0.733–0.843) | No | No |
| Marcolino et al., | 36 | No | Derivation: 806;  Validation: 208; | Yes | AUROC de 0.842 (95%  CI 0.840–0.843) | AUROC  of 0.859 (95% CI | No | Yes |

|  |  |  | External validation: 172 |  |  | 0.833 to 0.885);  External validation: AUROC = 0.894  (95% CI 0.870 to  0.919) |  |  |
| --- | --- | --- | --- | --- | --- | --- | --- | --- |
| Martínez- Lacalzada et al., | 29 | Yes | Derivation: 1598;  External validation: 594 | Yes | 0.823 (95%CI 0.813,  0.834) | 0.794 (95%CI 0.775,  0.813) | No | No |
| Mayneris- Perxachs et al., | Not clear | No | 266 | Yes | Receiver operating characteristic curve for the  logistic regression model in individuals with hemoglobin levels below the median: 81,6%;  Receiver operating characteristic curve for the logistic regression model in  individuals with hemoglobin levels above the median:  83,3% | Not clear | No | No |
| Mei et al., | 51 | Yes | 103 | Yes | Full model: 0.96 (95%  CI, 0.96 to 0.97) /  Simple model: 0.92  (95% CI, 0.89 to 0.95) | Full model: 0.97 (95%  CI, 0.96 to 0.98) /  Simple model: 0.88  (95% CI, 0.80 to 0.96) | No | Yes |
| Mei et al., | 43 | Yes | 105 | Yes | 0.912 (95% CI 0.878-  0.947) | VC1 = 0.928 (95% CI  0.884-0.971) and VC2  = 0.883 (0.815-0.952) | No | Yes |
| Mendizabal et al., | Not disclosed | Yes | 391 | No | 0.75 | NA | No | No |
| Momeni- Boroujeni et al., | 50 | Yes | 211 | No | No | NA | No | No |

| Monterde et al., | Not clear | No | Not clear | No | The AUC for prediction of critical illness was 0.641 (95% CI 0.624-  0.660) for the Charlson index, 0.665 (0.645-  0.681) for the Elixhauser index, and 0.787 (0.773-0.801) for  Queralt DxS. | NA | No | No |
| --- | --- | --- | --- | --- | --- | --- | --- | --- |
| Moulaei et al., | 67 | No | 144 | No | Random forest 99.02  XGBoost 98.18  KNN 96.78  MLP 96.49  Logistic regression 94.22  J48 decision tree 92.19 Naïve Bayes 92.05 | Not clear | No | No |
| Murri et al., | Not clear (~46) | No | 120 (derivation) +  332 (validation) | No | Training set (cross validation) 0.870 | Testing set 0.818 | No | No |
| Nascimento et al., | Not clear | Yes | 15 | No | 0,786 | NA | No | No |
| Neant et al., | Not clear (~5) | Not clear | 74 | No | Not disclosed | NA | No | No |
| Nguyen et al., | Not clear | No | 118 | No | 0.802 (0.029) | NA | No | No |
| Nicholson et al., | 49 | Yes | Not reported | Yes | 0.87 (0.83 – 0.91) | 0.80 (0.75 – 0.85) | No | No |
| Núñez-Gil et al., | Not clear | Yes | 311 | No | 0.88 (0.85–0.91) | Not clear | No | Yes |
| Obremska et al., | Not clear | No | Derivation: 27;  Validation: 8; Prospective cohort: 6 | Yes, described as prospective cohort | 0.89  (0.84–0.95) | Validation: 0.850  (0.75–0.88);  Prospective cohort: 0.773 (0.731–0.816) | No | No |

| Oh & Song | Not clear | Yes | 174 | \No | 0,83 | NA | No | No |
| --- | --- | --- | --- | --- | --- | --- | --- | --- |
| Oualim et al., | Not clear (interested in evaluating d-dimer biomarker) | Yes | 10 | No | Admission D-dimer levels: area under ROC curve 0.775 / D-dimer  levels day 5: area under ROC curve 0.964 | NA | No | No |
| Pan et al., | 100 | Yes | 58 | No | Logistic Regression: 0.84 (0.66-1.0);  AdaBoost: 0.91 (0.80-  1.0); GBDT: 0.85 (0.70-  1.0); XGBoost: 0.92  (0.81-1.0); CatBoost:  0.91 (0.8-1.0) | Not clear (they say "good agreement") | No | Yes |
| Peiró et al., | NA | Yes | 37 | No | cTnI 0.825 (95% CI  0.759–0.892); D-dimer  0.756 (95% CI 0.674–  0.837); CRP 0.685 (95%  CI 0.600–0.770); LDH  0.643 (95% CI 0.534–  0.753) | NA | No | No |
| [Philippe](https://www.ncbi.nlm.nih.gov/pubmed/?term=Philippe%20A%5BAuthor%5D&cauthor=true&cauthor_uid=33449299) et al., | Not clear | Yes | 41 | No | Von Willebrand factor: AUC 0.92, 95% CI  0.88–0.96; Soluble  thrombomodulin: 0.91,  95% CI 0.87–0.95; von  Willebrand factor low- molecular weight multimers: as a ratio vs normal plasma: 0.77,  95% CI 0.64–0.90, as a  percentage 0.64, 95% CI  0.49–0.79 | NA | No | No |
| Pigoga et al., | 34 | No | Not disclosed (239 total) | No | 0.86 | Not disclosed | No | Yes |

| Pimentel et al., | 7 | No | COVID: 99  Viral pneumonia: 29 | No | Not clear | NA | No | Yes |
| --- | --- | --- | --- | --- | --- | --- | --- | --- |
| Plečko et al., | 38 | Yes | 459 | Yes | 0.784 (0.761–0.808) | 0.745 (0.709–0.781) | No | No |
| Ponce et al., | > 33. The data collection sheet had the following variables: (1) country and city of residence, demographic data;  (2) comorbidities and condition at admission; (3)  laboratory values at admission; (4) characteristics and aetiology of AKI; (5) ICU admission, mechanical ventilation (MV) and in- hospital complications; and  (6) condition at discharge | Yes | 544 | No | Random Forest 0.894  [0.82–0.93] ]  XgBoost 0.886 [0.85–  0.96]  Elastic Net 0.877 [0.83–  0.97] | Random Forest 0.831  [0.76–0.89]  XgBoost 0.823 [0.75–  0.88]  Elastic Net 0.821  [0.75–0.88] | No | Yes |
| Qeadan et al., | Not disclosed | Yes | 104 | No | 0.9 | NA | No | No |
| Qin et al., | 46 | Yes | 41 | No | The AUC values of age, neutrophil count, lymphocyte count, prothrombin time, d- dimer and PCT for predicting the in- hospital mortality of COVID-19 patients were 0.808 (95% CI  0.715–0.901), 0.809  (95% CI 0.710–0.907),  0.811 (95% CI 0.724–  0.898), 0.745 (95% CI | NA | No | No |

|  |  |  |  |  | 0.643–0.847), 0.872  (95% CI 0.804–0.940),  0.881 (95% CI 0.809–  0.953 (Fig. (Fig.1),1),  respectively. While combining the bundle of risk factors selected by logistic regression, the AUC value rose to 0.992 (95% CI 0.981–  1.000) |  |  |  |
| --- | --- | --- | --- | --- | --- | --- | --- | --- |
| Qin et al., | 20 | Yes | 300 | No | Hs-cTnI 0.78 (0.73-  0.84) / CK-MB 0.71 (0.67-0.75) / (NT- Pro)BNP 0.81 (0.78- 0.85) / CK 0.67 (0.62- 0.72) / MYO 0.83 (0.80-  0.86) | NA | No | No |
| Romualdo et al., | 9 | No | 8 | No | Ferritin (ng/mL) 0.692  (0.566–0.800)  CRP (mg/dL) 0.791  (0.673–0.881)  D-dimer (μg/L FEU) 0.869 (0.763–0.939)  Calprotectin (mg/L) 0.801 (0.691–0.894)  GDF-15 (ng/L) 0.892  (0.792–0.955) | NA | No | No |
| Rothschild et al., | 41 | No | 23 | No | Not clear | NA | No | No |
| Ryan et al., | Not clear | No | 5083 | No | 12h: 0.865 (0.0027)  24h: 0.783 (0.0017)  48h: 0.769 (0.0074)  72h: 0.726 (0.0047) | NA | 12h: 0.467  (0.0094)  24h: 0.349  (0.0160)  48h: 0.374  (0.0158) | No |

|  |  |  |  |  |  |  | 72h: 0.357  (0.0070) |  |
| --- | --- | --- | --- | --- | --- | --- | --- | --- |
| Saldi et al., | Not clear (>27) | No | 160 | No | 84.7% (95% CI 0.82-  0.88) | No | No | Yes |
| Sankaranarayan an et al., | Not clear (approximately 54) | No | Not clear | Yes (temporal validation) | 0.938 (SE 0.004) | AUROC of 0.901 | No | No (but they adhere to CONSORT) |
| Sauzay et al., | Not disclosed | Yes | 32 | No | Not disclosed | Not disclosed | No | No |
| Schlauch et al., | Not clear | No | 7327 | No | Next-day,  next-3-days, next-7- days, and overall mortality, which resulted in an AUCROC of 0·911, 0·905,  0·901, and 0·905, respectively | 0.91 | No | Yes |
| Schöning et al., | Not clear | No | 25 (derivation and internal validation) | Yes | AUROC = 0.94 | AUROC = 0.85 | No | No |
| Sensusiati et al., | Not disclosed | Yes | 30 | No | 0,794 | NA | No | No |
| Shah et al., | 26 | No | Not clear | No | No | No | No | No |
| Shamout et al., | 58 | No | 538 | No | 24h / 48h / 72h / 96h Radiologist A 0.613 / 0.645 / 0.691 / 0.740  Radiologist B 0.637 / 0.636 / 0.658 / 0.713  Radiologist A + 0.642 / 0.663 / 0.692 / 0.741  Radiologist B (0.555,  0.729) / (0.589, 0.746) /  (0.621, 0.766) / (0.678,  0.809) | 24h / 48h / 72h / 96h COVID-GBM 0.747 /  0.739 / 0.750 / 0.770  COVID-GMIC 0.695 /  0.716 / 0.717 / 0.738  COVID-GBM + 0.765  / 0.749 / 0.769 / 0.786  COVID-GMIC (0.712,  0.817) / (0.700, 0.798)  / (0.724, 0.818) /  (0.745, 0.830) | Not clear | No |

|  |  |  |  |  | COVID-GMIC 0.642 /  0.701 / 0.751 / 0.808  COVID-GBM 0.704 /  0.719 / 0.750 / 0.787  COVID-GBM + / 0.708  / 0.702 / 0.778 / 0.819  COVID-GMIC (0.637,  0.799) / (0.633, 0.775) /  (0.719, 0.851) / (0.763,  0.885) |  |  |  |
| --- | --- | --- | --- | --- | --- | --- | --- | --- |
| Shang et al., | Not clear | Yes | 73 | No | Not disclosed | NA | No | No |
| Shang et al., | 52 | No | 49 | Yes | 0.919 (95% CI 0.870-  0.970) | 0.938 (95% CI 0.902-  0.973) | No | No |
| Shao et al., | 22 | Yes | Not disclosed | No | 0,92 | NA | No | No |
| Shayganfar et al., | Not clear | Yes | 38 | No | Only shown for individual variables | NA | No | No |
| Singh et al., | 57 | No | Not clear | Yes | 0.78, 95% CI: 0.77–0.82 | Not clear | No | No |
| Sinkovits et al., | Not disclosed | Yes | 25 | No | NA | NA | No | No |
| Sîrbu et al., | 139 | No | 91 | Yes | Not clear (localized) | No | Yes | No |
| Soto-Mota et al., | NA (they tested existing scores: LOW-HARM, qSOFA, MSL-COVID-19, NUTRI-CoV, and NEWS2  scores) | Yes | 47 | Yes (they are testing existing scores) | LOW-HARM 0.76  (95% CI 0.69 to 0.84),  qSOFA 0.61 (95% CI  0.53 to 0.69), MSL- COVID-19 0.64 (95%  CI 0.55 to 0.73),  NUTRI-CoV 0.60 (95%  CI 0.51 to 0.69) ,  NEWS2 0.65 (95% CI | NA | No | No |

| 0.56 to 0.75) v, and neutrophil to  lymphocyte ratio 0.65  (95% CI 0.57 to 0.73) | | | | | | | | |
| --- | --- | --- | --- | --- | --- | --- | --- | --- |
| Soto-Mota et al., | NA | No | 200 (50%) | No | NA | Provided by different cut-offs, ranging from 0.61 to 0.90 (95%  ranges from 0.59 to 0.93), with best AUC for 25 points (0.90  [95% CI 0.87-0.93]) | No | No |
| Sottile et al., | Age, gender, race, ethnicity, supplemental O2 (high flow nasal cannula, non-invasive ventilation), median hospital days, overall mortality, all mechanical (ventilation, median hospital days, median ICU days, median ventilator days, mortality), Scores (Median qSOFA, Median SOFA, Median CURB-65, Charlson Comorbidity Index, ARDS Mortality Model (transfusion FFP, transfusion PRBC, GCS, lactate, creatinine, mean bilirubin, mean arterial pH, mean PF, mean SpO2), Novel Predictors (mean D- Dimer, mean LDH, mean ALC, mean BUN, mean troponin, mean CK, mean  ALT, mean lactate). | No | 717 | No | 0,94 | 0,9 | Yes | No |

| Sottile et al., | 21 | No | Derivation: 5726;  Validation: 408 | No | Novel Variables 0.83  (0.83, 0.84) | Novel Variables 0.88  (0.87, 0.90) | Yes | No |
| --- | --- | --- | --- | --- | --- | --- | --- | --- |
| Sourij et al., | Not clear (~36) | Yes | 58 | No | 0.889 (95% CI: 0.837-  0.941) | 0.893 (95% CI: 0.801-  0.959) | No | No |
| Stachel et al., | 83 | No | 452 | Temporal validation | LR: 0.79; DT: 0.69;  GB:0.83; SVM: 0.77;  NN:0.82 (they also measured the prediction model daily during hospital stay) | 0.018 difference in the AUC between training  and validation sets | No | Yes |
| Sun et al., | 40 | Yes | Not clear | No | 0 days earlier: 0.997 ±  0.00; 3 days earlier:  0.969 ± 0.01; 6 days  earlier: 0.947 ±0.03; 9 days earlier:0.921  ±0.03; 12 days earlier:  0.914 ±0.02 | Not clear | No | No |
| Surme et al., | Not clear | Yes | 33 | No | Not disclosed | NA | No | No |
| Synolaki et al., | Not clear | Yes | 24 | Yes | 0,951 | 0,924 | No | Yes |
| Thomson et al., | NA | Yes | 38 | No | Did not report | NA | No | No |
| Turrini et al., | 14 | Yes | 98 | No | Only for some variables, as: age distribution and P/F ratio at presentation showed an optimal cut- point at 69 years (AUC 0.71,) and at 233 (AUC  0.73), respectively.  Inflammation, LDH and CRP showed an optimal  cut-point at 395 U/L (AUC 0.77) and at 124 | NA | No | No |

|  |  |  |  |  | mg/L (AUC 0.70)  respectively. Between the 29 patients tested, IL6 values showed an optimal cut-point at 3484 pg/mL (AUC  0.862). |  |  |  |
| --- | --- | --- | --- | --- | --- | --- | --- | --- |
| Vagliano et al., | Not clear (~40 variables) | No | 796 | No | Not clear (performance varies from fair [AUROC 0⋅7-0⋅8] to  very good [AUROC > 0⋅9]) | Not clear. Predictive performance of the autoML models with variables available at admission shows fair discrimination (average AUROC = 0·75-0·76 (sdev =  0·03), PPV = 0·70-  0·76 (sdev = 0·1) at cut-off = 0·3 (the observed mortality rate), and good calibration. This performance is on par with a logistic regression model with selection of patient variables by three experts (average AUROC = 0·78 (sdev  = 0·03) and PPV = 0·79 (sdev = 0·2)). Extending the models with variables that are available at 24 hours after admission resulted in models  with higher predictive | No | Yes |

|  |  |  |  |  |  | performance (average AUROC = 0·77-0·79  (sdev = 0·03) and PPV  = 0·79-0·80 (sdev = 0·10-0·17)). |  |  |
| --- | --- | --- | --- | --- | --- | --- | --- | --- |
| Van Dam et al., | Age, sex and information regarding comorbidity according to the Charlson Comorbidity Index (CCI), heart rate, mean arterial blood pressure, respiratory rate (RR), oxygen saturation, temperature and Glasgow Coma Scale (GCS), Supplemental oxygen. When the RR or GCS were missing, we used paCO2 and descriptions in the medical records to deduce these values. In addition, we collected blood urea nitrogen (BUN), lactate dehydrogenase (LDH), bilirubin, albumin, lymphocytes and C reactive protein (CRP). Finally, we retrieved data on length of hospital stay, admission to the ICU and mortality. | No | 167 | Yes | 0.84 | The model yielded an AUC of 0.77 (95% CI  0.73 to 0.81)  for 30-day mortality and an AUC of 0.72 (95% CI 0.68 to  0.76) for a composite of 30-day mortality and/or admission to  ICU. | No | Yes |
| Varol et al., | Not clear | Yes | 33 | No | AUC = 0.802, 95% CI 0.777‐0.886 | NA | No | No |
| Vela et al., | NA | No | 19,114 | No | 0.96 (0.96 – 0.96) | 0.96 (0.96 – 0.96) | No | No |

| Velasco- Rodriguez et al., | Not disclosed | Yes | 213 | No | 0,81 | 0,8 | No | Yes |
| --- | --- | --- | --- | --- | --- | --- | --- | --- |
| Vepa et al., | 44 | Yes | Not clear | No | NA | NA | 83.7% | Yes |
| Verma et al., | 28 | No | COVID-19: 204 /  Influenza: 48 | No | mAPACHE (AUC 0.86  for cases with complete data and 0.81 after imputation), CISSS (AUC 0.83 for cases with complete data and  0.80 after imputation) and ISARIC-4C (AUC  0.78 after imputation) | NA | No | No |
| Vicka et al., | Not clear | Yes | 103 | Yes | No | SOFA: 0.679 (0.611  0.747)  SAPS II: 0.755 (0.695  0.815)  APACHE II: 0.772  (0.714 0.830)  4C Mortality: 0.754  (0.694 0.814) | No | No |
| Vila et al., | 15 | Yes | 53 | Yes | 0,845 | Not disclosed | No | No |
| Wang et al., | Not clear | Yes | 142 | No | NA | NA | No | No |
| Wang et al., | Not clear | Yes | 5 | No | NLR + D-dimer: AUC of 0.916 (95% CI  0.855–0.977) / SOFA  and NLR yielded an AUC of 0.750 (95% CI  0.602–0.987) and 0.862  (95% CI 0.751–0.973) | NA | No | No |
| Wang et al., | 38 | Yes | 19 | No | Clinical model 0.883  (0.799, 0.949) / | Clinical model 0.829  (0.678, 0.928) / | No | No |

|  |  |  |  |  | Laboratory model 0.976  (0.923, 0.994) | Laboratory model 0.878 (0.752, 0.958) |  |  |
| --- | --- | --- | --- | --- | --- | --- | --- | --- |
| Wang et al., | Not disclosed | Yes | 21 | NA | 0,78 | NA | No | No |
| Wang et al., | 20 | No | 615 | No | 24 HOURS: Logistic regression model 0.91 / XGBoost 0.92  Logistic regression was able to predict a deceased outcome 4 days in advance (AUC=0.82;  specificity=85%; and sensitivity=71%) and 5 days in advance (AUC=0.81;  specificity=70%; and sensitivity=75%) for XGBoost | Not clear | No | No |
| Wang et al., | 41 | No | 24 | Yes | 0.871 (based on its optimal cut-off value =  85) | Not available (link for supplemental material  does not work) | No | No |
| Wang et al., | Not clear | No | 40 (training);  10 (test);  4 (external validation) | Yes | Not clear | AUCs of 0.876 (95%  confidence interval: 0.752-  0.999) and 0.864  (0.766-0.962) in test and external test sets | No | No |
| Weng et al., | 24 | No | 21 | Yes | 0.921 (0.835-0.968) | 0.975 (0.947-1.0) | No | No |
| Williams et al., | 31917 | No | 11407 | Yes | 0.896 (95% CI 0.72 -  0.90) | CUIMC database 0.820 (95% CI 0.796-  0.840); HIRA  database 0.898 (95%  CI 0.857-0.940); | No | Yes |

|  |  |  |  |  |  | SIDIAP 0.895 (95%  CI 0.881-0.910); VA  0.717 (0.642-0.791) |  |  |
| --- | --- | --- | --- | --- | --- | --- | --- | --- |
| Wongvibulsin et al., | 105 | No | Not disclosed | No | Not disclosed | Not disclosed | No | Yes |
| Wu et al., | 128 | Yes | Not clear | No | AUC 0.955 | AUC 0.945 | No | No |
| Xie et al., | 28 | No | 155 | Yes | 0.880 (95% CI not  reported) | 0.980 (0.958-1.00) | No | Yes |
| Yan et al., | 29 | Yes | 78 | No | Not clear | NA | No | No |
| Yasukawaet al., | Not clear | Yes | 9 | No | Not disclosed | NA | No | No |
| Yavuz et al., | Not clear | Yes | 23 | No | 0,715 | NA | No | No |
| Yoo et al., | 48 | Yes | Not reported | Yes | Not reported, as AUC was used to define the variables for the score. | At admission 0.81; maximum through admission 0.91; mean through admission 0.92 | No | No |
| Yu et al., |  | Yes | 18 | No | 0.971 (95% CI 0.928 to  0.992) | NA | No | No |
| Yu et al., | 34 | No | Not clear (506 total) | No | 0,9 | NA | No | No |
| Zayat et al., | Not clear | Yes | 8 | No | AUC of 0.87 | NA | No | No |

| Zeng et al., | 35 | Yes | Not disclosed | No | 0,979 | 0,954 | No | No |
| --- | --- | --- | --- | --- | --- | --- | --- | --- |
| Zhang et al., | 47 | Yes | 15 | No | Not clear | NA | No | No |
| Zhang et al., | 30 | No | 96 | Yes | 0.886 (95% CI 0.873–  0.899) | 0.879 (95% CI, 0.856–  0.900) and 0.839 (95%  CI [0.798–0.880) for  each one of the hospitals | No | No |
| Zhang et al., | 19 | No | 33 (4.3%) | Yes | DCS: 0.79; DCS: 0.89;  DL: 0.91 (95% CI not  reported) | DL: 0.74 (95% CI not  reported) | No | Yes |
| Zhao et al., | Age, Sex, Ethnicity (Hispanic/Latino, Non- Hispanic/Latino, Unknown), Race (Caucasian, African American or Others), Smoking history, Diabetes , Hypertension, Asthma, COPD, Coronary artery disease, Heart failure, Cancer, Immunosuppression, Chronic kidney disease, Fever, Cough, Shortness of breath, Fatigue, Sputum, Myalgia, Diarrhea, Nausea or vomiting, Sore throat, Rhinorrhea, Loss of smell, Loss of taste, Headache, Chest discomfort or chest pain,  Abnormal chest x-ray | No | 82 | No | 0.87 ([95%  CI, 0.83–0.92],  p<0.001) for mortality | 0.82 ([95% CI, 0.73–  0.92], p<0.001) | No | Yes |

|  | results, Chest x-ray findings (Unilateral and Bilateral),  Heart Rate, Respiratory rate, SpO2%, Systolic blood pressure, Temperature, Alanine aminotransferase, Brain natriuretic peptide, C- reactive protein, D-dimer, Ferritin,  Lactate dehydrogenase, LeUnited Kingdomocytes, Lymphocytes, Procalcitonin, Troponin. |  |  |  |  |  |  |  |
| --- | --- | --- | --- | --- | --- | --- | --- | --- |
| Zhou et al., | Age, sex (female or male), exposure history, current smoker, hypertension, diabetes, coronary heart disease, chronic obstructive lung disease, carcinoma, chronic kidney disease, other, respiratory rate (>24 breaths per min), pulse  ≥125 beats per min, systolic blood pressure <90 mm hg, fever (temperature  ≥37.3°c), cough, sputum, myalgia, fatigue, diarrhoea, nausea or vomiting, SOFA score, qSOFA score, disease severity status, time from illness onset to hospital admission, white  blood cell count, lymphocyte count, | Yes, univariable analysis | 54 | No | No | No | No | No |

| haemoglobin, anaemia, platelet count, albumin, alt, creatinine, lactate dehydrogenase, creatine kinase, high-sensitivity cardiac troponin i, prothrombin time, D-dimer, serum ferritin, IL-6, procalcitonin, imaging features: (A) consolidation,   1. ground-glass opacity , 2. bilateral pulmonary infiltration. | | | | | | | | |
| --- | --- | --- | --- | --- | --- | --- | --- | --- |
| Zhou et al., | 37 | No | 51 | No | 0.955 (95% CI not  provided) | NA | No | No |
| Zhu et al., | Not clear | Yes | 14 | NA | 0,96 | NA | No | No |
| Zinellu et al., | 9 | Yes | 28 | No | ALT 0.519 0.418‐0.618  AST 0.616 0.515‐0.710  De Ritis ratio 0.701 0.603‐0.787 | NA | No | No |

ARDS: acute respiratory distress syndrome; AST: aspartate transaminase; AUC: area under the curve; BMI: body mass index; BUN: blood urea nitrogen; CCEDRRN: canadian COVID- 19 emergency department rapid response network; CI: confidence interval; CKD: chronic kidney disease; COPD: chronic obstructive pulmonary disease; CPR: C-reactive protein; CT: computed tomography; DLN: deep learning networks; DM: diabetes mellitus; ED: emergency department; EH: emergency hospital; ER: emergency room; FiO2: fraction of inspired oxygen; GFR: glomerular filtration rate; GP: general practice; ICU: intensive care unit; IHD: ischemic heart disease; IL-6: interleucin 6; INR: international normalized ratio; LASSO: least absolute shrinkage and selection operator logistic regression; LDH: lactate dehydrogenase; MAP: mean arterial pressure; MICE: Multivariate Imputation by Chained Equations; NA: not applicable; NAAT: nucleic acid amplification test; NEWS2: national early warning score; NLR: neutrophil lymphocyte ratio; OP: outpatient; PLS: partial least squares; RDW: red blood cell distribution width; RF: Random Forest; RT-PCR: reverse transcription polymerase chain reaction; SF ratio: SpO2/FiO2 ratio; SVM: support-vector machine; Trop I: troponin I; XGBoost: eXtreme Gradient Boosting; WBC: white blood cell; WHO: World Health Organization.

**Table S2. Potential predictors included for the development of the models**

| **Variables** |
| --- |
| **Demographics characteristics** |
| Sex at birth |
| Age (years) |
| **Comorbidities and lifestyle habits** |
| Hypertension |
| Coronary artery disease |
| Heart failure |
| Atrial fibrillation or flutter |
| Stroke |
| Chagas disease |
| Rheumatic heart disease |
| Other cardiovascular disease |
| No relevant cardiovascular disease |
| Asthma |
| COPD |
| Pulmonary fibrosis |
| Diabetes mellitus |
| Obesity (BMI>30kg/m^2^) |
| Cirrhosis |
| Psychiatry disease |
| Chronic kidney disease |
| Rheumatologic disease |
| HIV infection |
| Cancer |
| Previous organ transplantation |
| Immunosuppressive condition |
| Another relevant health condition |

| Number of comorbidities |
| --- |
| Number of cardiovascular comorbidities |
| Number of comorbidities in different groups of diseases |
| Illegal drug use |
| Alcoholism |
| Current smoker |
| Ex-smoker |
| **Clinical characteristics** |
| Time from symptom onset |
| Respiratory rate (irpm) |
| Heart rate (bpm) |
| Systolic blood pressure (mm Hg) |
| Diastolic blood pressure (mm Hg) |
| Inotrope use |
| Glasgow coma score |
| SF ratio |
| FiO2 |
| **Laboratory** |
| C reactive protein (mg/L) |
| Hemoglobin (g/L) |
| Leucocytes (10^9^/L) |
| Neutrophils (10^9^/L) |
| Lymphocytes (10^9^/L) |
| Neutrophils-to-lymphocytes ratio |
| Platelet count (10^9^/L) |
| Creatinine (mg/dL) |
| Urea (mg/dL) |
| Lactate (mmol/L) |
| Sodium (mmol/L) |

| Bicarbonate (mEq/L) |
| --- |
| pH |
| pO2 (mmHg) |
| pCO2 (mmHg) |
| D-dimer |

COPD: chronic obstructive pulmonary disease; FiO2: fraction of inspired oxygen; HIV: human immunodeficiency virus; pCO2: partial pressure of carbon dioxide; pO2: partial pressure of oxygen; SF ratio: SpO2/FiO2 ratio.

# Table S3. Additional model results

|  | **MICRO-F1**  **mean** | **MACRO-F1**  **mean** | **PRECISION**  **mean** | **RECALL**  **mean** | **LOGLOSS**  **mean** | **AUROC**  **mean** |
| --- | --- | --- | --- | --- | --- | --- |
| **STACKING** | **0,821** | **0,654** | **0,562** | **0,354** | **6,032** | **0,826** |
| **LGBM** | **0,825** | **0,648** | **0,555** | **0,345** | 6,177 | **0,824** |
| **LGBM + PCA** | 0,802 | 0,586 | 0,544 | 0,225 | 6,839 | 0,788 |
| **LGBM + SVD** | 0,792 | 0,546 | 0,386 | 0,181 | 7,84 | 0,781 |

LGBM: light gradient boosting machines; PCA: principal component analysis; STACKING: a stacking classifier, which combines the outputs of all others; SVD: singular value decomposition.

# REFERENCES

1. Abbasi B., et al. Evaluation of the relationship between inpatient COVID-19 mortality and chest CT severity score. *Am J Emerg Med*. 2021;45:458-463. doi:10.1016/j.ajem.2020.09.056.
2. Abdollahpour I., et al. Model Prediction for In-Hospital Mortality in Patients with COVID-19: A Case-Control Study in Isfahan, Iran. *Am J Trop Med Hyg.* 2021 Feb 16;104(4):1476-1483. doi: 10.4269/ajtmh.20-1039. PMID: 33591938; PMCID: PMC8045635.
3. Abdulaal A., et al. Prognostic Modeling of COVID-19 Using Artificial Intelligence in the United Kingdom: Model Development and Validation. *J Med Internet Res.* 2020 Aug 25;22(8):e20259. doi: 10.2196/20259. PMID: 32735549; PMCID: PMC7451108.
4. Acar, H.C., et al. An easy-to-use nomogram for predicting in-hospital mortality risk in COVID-19: a retrospective cohort study in a university hospital. *BMC Infect Dis* 21, 148 (2021). https://doi.org/10.1186/s12879-021-05845-x.
5. Adderley N.J., et al. Development and external validation of prognostic models for COVID-19 to support risk stratification in secondary care. *BMJ Open*. 2022 Jan 17;12(1):e049506. doi: 10.1136/bmjopen-2021-049506. PMID: 35039282; PMCID: PMC8764710.
6. Agarwal N., et al. COVID-19 mortality prediction model, 3C-M, built for use in resource limited settings - understanding the relevance of neutrophilic leUnited Kingdomocytosis in predicting disease severity and mortality. *MedRxiv.* 2021. doi: https://doi.org/10.1101/2021.08.05.21261565.
7. Ageno W., et al. Clinical risk scores for the early prediction of severe outcomes in patients hospitalized for COVID-19. *Intern Emerg Med.* 2021 Jun;16(4):989-996. doi: 10.1007/s11739-020-02617-4. Epub 2021 Feb 23. PMID: 33620680; PMCID: PMC7900378.
8. Akdur G., et al. Prediction of mortality in COVID-19 through combing CT severity score with NEWS, qSOFA, or peripheral perfusion index. *Am J Emerg Med.* 2021 Dec;50:546-552. doi: 10.1016/j.ajem.2021.08.079. Epub 2021 Sep 2. PMID: 34547696; PMCID: PMC8411577.
9. Aliberti MJR., et al. A fuller picture of COVID-19 prognosis: the added value of vulnerability measures to predict mortality in hospitalised older adults. *Age Ageing.* 2021

Jan 8;50(1):32-39. doi: 10.1093/ageing/afaa240. PMID: 33068099; PMCID: PMC7665299.

1. Allenbach Y., et al. Development of a multivariate prediction model of intensive care unit transfer or death: A French prospective cohort study of hospitalized COVID-19 patients. *PloS one.* 2020;15(10):e0240711-e.
2. Altini N., et al. Predictive Machine Learning Models and Survival Analysis for COVID-19 Prognosis Based on Hematochemical Parameters. *Sensors (Basel).* 2021 Dec 20;21(24):8503. doi: 10.3390/s21248503. PMID: 34960595; PMCID: PMC8705488.
3. Altschul DJ., et al*.* A novel severity score to predict inpatient mortality in COVID- 19 patients. *Scientific Reports.* 2020;10(1):1-8,16726.
4. Avendaño-Ortiz J., et al. The immune checkpoints storm in COVID-19: Role as severity markers at emergency department admission. *Clin Transl Med.* 2021 Oct;11(10):e573. doi: 10.1002/ctm2.573. PMID: 34709745; PMCID: PMC8521292.
5. Aznar-Gimeno R., et al. A Clinical Decision Web to Predict ICU Admission or Death for Patients Hospitalised with COVID-19 Using Machine Learning Algorithms. *Int J Environ Res Public Health.* 2021 Aug 17;18(16):8677. doi: 10.3390/ijerph18168677. PMID: 34444425; PMCID: PMC8394359.
6. Bai T., et al. Reliable and Interpretable Mortality Prediction With Strong Foresight in COVID-19 Patients: An International Study From China and Germany. *Front Artif Intell.* 2021 Sep 3;4:672050. doi: 10.3389/frai.2021.672050. PMID: 34541519; PMCID: PMC8446629.
7. Banoei MM, Dinparastisaleh R, Zadeh AV, Mirsaeidi M. Machine-learning-based COVID-19 mortality prediction model and identification of patients at low and high risk of dying. *Crit Care.* 2021 Sep 8;25(1):328. doi: 10.1186/s13054-021-03749-5. PMID: 34496940; PMCID: PMC8424411.
8. Baqui P., et al*.* Comparing COVID-19 risk factors in Brazil using machine learning: the importance of socioeconomic, demographic and structural factors. *Sci Rep.* 2021 Aug 2;11(1):15591. doi: 10.1038/s41598-021-95004-8. PMID: 34341397; PMCID: PMC8329284.
9. Bartoszko J., et al*.* Development of a repeated-measures predictive model and clinical risk score for mortality in ventilated COVID-19 patients. *Can J Anaesth.* 2022 Mar;69(3):343-352. doi: 10.1007/s12630-021-02163-3. Epub 2021 Dec 20. PMID: 34931293; PMCID: PMC8687635.
10. Bello-Chavolla O.Y., et al*.* Predicting Mortality Due to SARS-CoV-2: A Mechanistic Score Relating Obesity and Diabetes to COVID-19 Outcomes in Mexico. *J Clin Endocrinol Metab.* 2020 Aug 1;105(8):dgaa346. doi: 10.1210/clinem/dgaa346. PMID: 32474598; PMCID: PMC7313944.
11. Bello-Chavolla O.Y., et al*.*Validation and repurposing of the MSL-COVID-19 score for prediction of severe COVID-19 using simple clinical predictors in a triage setting: The Nutri-CoV score. *PLoS One.* 2020 Dec 16;15(12):e0244051. doi: 10.1371/journal.pone.0244051. PMID: 33326502; PMCID: PMC7743927.
12. Bennouar S, Bachir Cherif A, Kessira A, Bennouar DE, Abdi S. Development and validation of a laboratory risk score for the early prediction of COVID-19 severity and in-hospital mortality. *Intensive Crit Care Nurs.* 2021 Jun;64:103012. doi: 10.1016/j.iccn.2021.103012. Epub 2021 Jan 9. PMID: 33487518; PMCID: PMC7834685.
13. Bennouar S, et al. Vitamin D Deficiency and Low Serum Calcium as Predictors of Poor Prognosis in Patients with Severe COVID-19*. J Am Coll Nutr*. 2021;40(2):104- 110. doi:10.1080/07315724.2020.1856013.
14. Berenguer J., et al. Development and validation of a prediction model for 30-day mortality in hospitalised patients with COVID-19: the COVID-19 SEIMC score. *Thorax.* 2021 Sep;76(9):920-929. doi: 10.1136/thoraxjnl-2020-216001. Epub 2021 Feb 25. PMID: 33632764; PMCID: PMC7908055.
15. Berry D.A., et al. Development and validation of a prognostic 40-day mortality risk model among hospitalized patients with COVID-19. *PLoS One.* 2021 Jul 30;16(7):e0255228. doi: 10.1371/journal.pone.0255228. PMID: 34329317; PMCID: PMC8323891.
16. Bertsimas D., et al. COVID-19 mortality risk assessment: An international multi- center study. *PLoS One.* 2020;15(12):e0243262.
17. Boudou M, ÓhAiseadha C, Garvey P, O'Dwyer J, Hynds P. Modelling COVID- 19 severity in the Republic of Ireland using patient co-morbidities, socioeconomic profile and geographic location, February to November 2020. *Sci Rep.* 2021 Sep 16;11(1):18474. doi: 10.1038/s41598-021-98008-6. PMID: 34531478; PMCID: PMC8446039.
18. Brieghel C., et al. Prognostic factors of 90-day mortality in patients hospitalised with COVID-19. *Dan Med J.* 2021 Feb 22;68(3):A09200705. PMID: 33660609.
19. Cai J., et al. The Neutrophil-to-Lymphocyte Ratio Determines Clinical Efficacy of Corticosteroid Therapy in Patients with COVID-19. *Cell Metab.* 2021 Feb

2;33(2):258-269.e3. doi: 10.1016/j.cmet.2021.01.002. Epub 2021 Jan 5. PMID:

33421384; PMCID: PMC7832609.

1. Castro VM, McCoy TH, Perlis RH. Assessment of the Performance Consistency of an Adverse Outcome Prediction Tool for Patients Hospitalized With COVID-19. *JAMA Netw Open.* 2021 Jul 1;4(7):e2118413. doi: 10.1001/jamanetworkopen.2021.18413. PMID: 34313745; PMCID: PMC8317002.
2. Chen R., et al. Risk Factors of Fatal Outcome in Hospitalized Subjects With Coronavirus Disease 2019 From a Nationwide Analysis in China. *Chest.* 2020;158(1):97- 105.
3. Cheng A, Hu L, Wang Y, Huang L, Zhao L, Zhang C, Liu X, Xu R, Liu F, Li J, Ye D, Wang T, Lv Y, Liu Q. Diagnostic performance of initial blood urea nitrogen combined with D-dimer levels for predicting in-hospital mortality in COVID-19 patients. Int J Antimicrob Agents. 2020 Sep;56(3):106110. doi: 10.1016/j.ijantimicag.2020.106110. Epub 2020 Jul 23. PMID: 32712332; PMCID: PMC7377803.
4. Cho SY, Park SS, Song MK, Bae YY, Lee DG, Kim DW. Prognosis Score System to Predict Survival for COVID-19 Cases: a Korean Nationwide Cohort Study. J Med Internet Res. 2021 Feb 22;23(2):e26257. doi: 10.2196/26257. PMID: 33539312; PMCID: PMC7901599.
5. Chow DS, Glavis-Bloom J, Soun JE, Weinberg B, Loveless TB, Xie X, Mutasa S, Monuki E, Park JI, Bota D, Wu J, Thompson L, Boden-Albala B, Khan S, Amin AN, Chang PD. Development and external validation of a prognostic tool for COVID-19 critical disease. PLoS One. 2020 Dec 9;15(12):e0242953. doi: 10.1371/journal.pone.0242953. PMID: 33296357; PMCID: PMC7725393.
6. Chua F, Vancheeswaran R, Draper A, Vaghela T, Knight M, Mogal R, Singh J, Spencer LG, Thwaite E, Mitchell H, Calmonson S, Mahdi N, Assadullah S, Leung M, O'Neill A, Popat C, Kumar R, Humphries T, Talbutt R, Raghunath S, Molyneaux PL, Schechter M, Lowe J, Barlow A. Early prognostication of COVID-19 to guide hospitalisation versus outpatient monitoring using a point-of-test risk prediction score. Thorax. 2021 Jul;76(7):696-703. doi: 10.1136/thoraxjnl-2020-216425. Epub 2021 Mar 10. PMID: 33692174; PMCID: PMC7948158.
7. Chung H, Ko H, Kang WS, Kim KW, Lee H, Park C, Song HO, Choi TY, Seo JH, Lee J. Prediction and Feature Importance Analysis for Severity of COVID-19 in South Korea Using Artificial Intelligence: Model Development and Validation. J Med

Internet Res. 2021 Apr 19;23(4):e27060. doi: 10.2196/27060. PMID: 33764883; PMCID: PMC8057199.

1. Churpek MM, Gupta S, Spicer AB, Hayek SS, Srivastava A, Chan L, et al. Machine Learning Prediction of Death in Critically Ill Patients With Coronavirus Disease 2019. Crit Care Explor. 2021;3(8):e0515.
2. Clift AK, Coupland CAC, Keogh RH, Diaz-Ordaz K, Williamson E, Harrison EM, Hayward A, Hemingway H, Horby P, Mehta N, Benger J, Khunti K, Spiegelhalter D, Sheikh A, Valabhji J, Lyons RA, Robson J, Semple MG, Kee F, Johnson P, Jebb S, Williams T, Hippisley-Cox J. Living risk prediction algorithm (QCOVID) for risk of hospital admission and mortality from coronavirus 19 in adults: national derivation and validation cohort study. BMJ. 2020 Oct 20;371:m3731. doi: 10.1136/bmj.m3731. PMID: 33082154; PMCID: PMC7574532.
3. Corradi F, Isirdi A, Malacarne P, Santori G, Barbieri G, Romei C, Bove T, Vetrugno L, Falcone M, Bertini P, Guarracino F, Landoni G, Forfori F; UCARE (Ultrasound in Critical care and Anesthesia Research Group). Low diaphragm muscle mass predicts adverse outcome in patients hospitalized for COVID-19 pneumonia: an exploratory pilot study. Minerva Anestesiol. 2021 Apr;87(4):432-438. doi: 10.23736/S0375-9393.21.15129-6. Epub 2021 Feb 17. PMID: 33594871.
4. Covino M, De Matteis G, Polla DAD, Santoro M, Burzo ML, Torelli E, Simeoni B, Russo A, Sandroni C, Gasbarrini A, Franceschi F. Predictors of in-hospital mortality AND death RISK STRATIFICATION among COVID-19 PATIENTS aged ≥ 80 YEARs OLD. Arch Gerontol Geriatr. 2021 Jul-Aug;95:104383. doi: 10.1016/j.archger.2021.104383. Epub 2021 Feb 25. PMID: 33676091; PMCID: PMC7904458.
5. Dashti H, Roche EC, Bates DW, Mora S, Demler O. SARS2 simplified scores to estimate risk of hospitalization and death among patients with COVID-19. *Sci Rep*. 2021;11(1):4945. Published 2021 Mar 2. doi:10.1038/s41598-021-84603-0.
6. Deng F, Zhang L, Lyu L, et al. Increased levels of ferritin on admission predicts intensive care unit mortality in patients with COVID-19. El incremento de ferritina sérica durante el ingreso predice la mortalidad de los pacientes de COVID-19 en Cuidados Intensivos. *Med Clin (Barc)*. 2021;156(7):324-331. doi:10.1016/j.medcli.2020.11.030.
7. Di Castelnuovo A, Bonaccio M, Costanzo S, et al. Common cardiovascular risk factors and in-hospital mortality in 3,894 patients with COVID-19: survival analysis and

machine learning-based findings from the multicentre Italian CORIST Study. *Nutr Metab Cardiovasc Dis*. 2020;30(11):1899-1913. doi:10.1016/j.numecd.2020.07.031.

1. Ding ZY, Li GX, Chen L, et al. Association of liver abnormalities with in-hospital mortality in patients with COVID-19. *J Hepatol*. 2021;74(6):1295-1302. doi:10.1016/j.jhep.2020.12.012.
2. Doganci S, Ince ME, Ors N, et al. A new COVID-19 prediction scoring model for in-hospital mortality: experiences from Turkey, single center retrospective cohort analysis. *Eur Rev Med Pharmacol Sci*. 2020;24(19):10247-10257. doi:10.26355/eurrev_202010_23249.
3. Doher MP, Torres de Carvalho FR, Scherer PF, et al. Acute Kidney Injury and Renal Replacement Therapy in Critically Ill COVID-19 Patients: Risk Factors and Outcomes: A Single-Center Experience in Brazil. *Blood Purif*. 2021;50(4-5):520-530. doi:10.1159/000513425.
4. Domínguez-Olmedo JL, Gragera-Martínez Á, Mata J, Pachón Álvarez V. Machine Learning Applied to Clinical Laboratory Data in Spain for COVID-19 Outcome Prediction: Model Development and Validation. *J Med Internet Res*. 2021;23(4):e26211. Published 2021 Apr 14. doi:10.2196/26211.
5. Ebrahimi V, Sharifi M, Mousavi-Roknabadi RS, et al. Predictive determinants of overall survival among re-infected COVID-19 patients using the elastic-net regularized Cox proportional hazards model: a machine-learning algorithm. *BMC Public Health*. 2022;22(1):10. Published 2022 Jan 5. doi:10.1186/s12889-021-12383-3.
6. El-Raheem GOH, Yousif MAA, Mohamed DSI. Prediction of COVID-19 mortality among hospitalized patients in Sudan. MedRxiv. 2021. **doi:** https://doi.org/10.1101/2021.03.09.21253179.
7. Eskandar EN, Altschul DJ, de la Garza Ramos R, et al. Neurologic Syndromes Predict Higher In-Hospital Mortality in COVID-19 [published correction appears in Neurology. 2021 Mar 16;96(11):551]. *Neurology*. 2021;96(11):e1527-e1538. doi:10.1212/WNL.0000000000011356.
8. Faisal M, Mohammed MA, Richardson D, Fiori M, Beatson K. Development and validation of automated computer aided-risk score for predicting the risk of in-hospital mortality using first electronically recorded blood test results and vital signs for COVID- 19 hospital admissions: a retrospective development and validation study. MedRxiv. 2020. doi: 10.1101/2020.11.30.20241273.
9. Fan X, Zhu B, Nouri-Vaskeh M, et al. Scores based on neutrophil percentage and lactate dehydrogenase with or without oxygen saturation predict hospital mortality risk in severe COVID-19 patients. *Virol J*. 2021;18(1):67. Published 2021 Mar 31. doi:10.1186/s12985-021-01538-8.
10. Fernandes FT, de Oliveira TA, Teixeira CE, Batista AFM, Dalla Costa G, Chiavegatto Filho ADP. A multipurpose machine learning approach to predict COVID- 19 negative prognosis in São Paulo, Brazil. *Sci Rep*. 2021;11(1):3343. Published 2021 Feb 8. doi:10.1038/s41598-021-82885-y.
11. Fumagalli C, Rozzini R, Vannini M, Coccia F, Cesaroni G, Mazzeo F, et al. Clinical risk score to predict in-hospital mortality in COVID-19 patients: a retrospective cohort study. BMJ open. 2020;10(9):e040729-e.
12. Galiero R, Pafundi PC, Simeon V, et al. Impact of chronic liver disease upon admission on COVID-19 in-hospital mortality: Findings from COVOCA study. *PLoS One*. 2020;15(12):e0243700. Published 2020 Dec 10. doi:10.1371/journal.pone.0243700.
13. Galloway JB, Norton S, Barker RD, Brookes A, Carey I, Clarke BD, et al. A clinical risk score to identify patients with COVID-19 at high risk of critical care admission or death: an observational cohort study. Journal of Infection. 2020;81(2):282- 8.
14. Garrafa E, Vezzoli M, Ravanelli M, Farina D, Borghesi A, Calza S, et al. Early Prediction of In-Hospital Death of COVID-19 Patients: A Machine-Learning Model Based on Age, Blood Analyses, and Chest X-Ray Score. MedRxiv. 2021. doi: 10.1101/2021.06.10.21258721.
15. Gatti M, Calandri M, Biondo A, et al. Emergency room comprehensive assessment of demographic, radiological, laboratory and clinical data of patients with COVID-19: determination of its prognostic value for in-hospital mortality. *Intern Emerg Med*. 2022;17(1):205-214. doi:10.1007/s11739-021-02669-0.
16. Girardin JL, Seixas A, Ramos Cejudo J, et al. Contribution of pulmonary diseases to COVID-19 mortality in a diverse urban community of New York. *Chron Respir Dis*. 2021;18:1479973120986806. doi:10.1177/1479973120986806.
17. Gomez NFP, Lobo IM, Cremades IG, Tejerina AF, Rueda FR, Teleki AV, et al. [Potential biomarkers predictors of mortality in COVID-19 patients in the Emergency Department]. Rev Esp Quimioter. 2020;33(4):267-73.
18. Gopalan N, Senthil S, Prabakar NL, et al. Predictors of mortality among hospitalized COVID-19 patients and risk score formulation for prioritizing tertiary care-

An experience from South India. *PLoS One*. 2022;17(2):e0263471. Published 2022 Feb 3. doi:10.1371/journal.pone.0263471.

1. Gorham J, Moreau A, Corazza F, et al. Interleukine-6 in critically ill COVID-19 patients: A retrospective analysis. *PLoS One*. 2020;15(12):e0244628. Published 2020 Dec 31. doi:10.1371/journal.pone.0244628.
2. Guan X, Zhang B, Fu M, et al. Clinical and inflammatory features based machine learning model for fatal risk prediction of hospitalized COVID-19 patients: results from a retrospective cohort study. *Ann Med*. 2021;53(1):257-266. doi:10.1080/07853890.2020.1868564.
3. Gude-Sampedro F, Fernández-Merino C, Ferreiro L, et al. Development and validation of a prognostic model based on comorbidities to predict COVID-19 severity: a population-based study. *Int J Epidemiol*. 2021;50(1):64-74. doi:10.1093/ije/dyaa209.
4. Gue YX, Tennyson M, Gao J, Ren S, Kanji R, Gorog DA. Development of a novel risk score to predict mortality in patients admitted to hospital with COVID-19. Sci Rep. 2020;10(1):21379-.
5. Gupta RK, Harrison EM, Ho A, et al. Development and validation of the ISARIC 4C Deterioration model for adults hospitalised with COVID-19: a prospective cohort study. *Lancet Respir Med*. 2021;9(4):349-359. doi:10.1016/S2213-2600(20)30559-2.
6. Gupta A, Kachur SM, Tafur JD, et al. Development and Validation of a Multivariable Risk Prediction Model for COVID-19 Mortality in the Southern United States. *Mayo Clin Proc*. 2021;96(12):3030-3041. doi:10.1016/j.mayocp.2021.09.002.
7. Hajifathalian K, Sharaiha RZ, Kumar S, Krisko T, Skaf D, Ang B, et al. Development and external validation of a prediction risk model for short-term mortality among hospitalized U.S. COVID-19 patients: A proposal for the COVID-AID risk tool. PloS one. 2020;15(9):e0239536-e.
8. Halalau A, Imam Z, Karabon P, Mankuzhy N, Shaheen A, Tu J, et al. External validation of a clinical risk score to predict hospital admission and in-hospital mortality in COVID-19 patients. Annals of medicine. 2021;53(1):78-86.
9. He X, Yao F, Chen J, et al. The poor prognosis and influencing factors of high D- dimer levels for COVID-19 patients. *Sci Rep*. 2021;11(1):1830. Published 2021 Jan 19. doi:10.1038/s41598-021-81300-w.
10. He X, Wang L, Wang H, et al. Factors associated with acute cardiac injury and their effects on mortality in patients with COVID-19. *Sci Rep*. 2020;10(1):20452. Published 2020 Nov 24. doi:10.1038/s41598-020-77172-1.
11. He F, Page JH, Weinberg KR, Mishra A. The Development and Validation of Simplified Machine Learning Algorithms to Predict Prognosis of Hospitalized Patients With COVID-19: Multicenter, Retrospective Study. *J Med Internet Res*. 2022;24(1):e31549. Published 2022 Jan 21. doi:10.2196/31549.
12. Heber S, Pereyra D, Schrottmaier WC, et al. A Model Predicting Mortality of Hospitalized Covid-19 Patients Four Days After Admission: Development, Internal and Temporal-External Validation. *Front Cell Infect Microbiol*. 2022;11:795026. Published 2022 Jan 24. doi:10.3389/fcimb.2021.795026.
13. Heldt FS, Vizcaychipi MP, Peacock S, et al. Early risk assessment for COVID-19 patients from emergency department data using machine learning. *Sci Rep*. 2021;11(1):4200. Published 2021 Feb 18. doi:10.1038/s41598-021-83784-y.
14. Heller RA, Sun Q, Hackler J, et al. Prediction of survival odds in COVID-19 by zinc, age and selenoprotein P as composite biomarker. *Redox Biol*. 2021;38:101764. doi:10.1016/j.redox.2020.101764.
15. Her AY, Bhak Y, Jun EJ, et al. A Clinical Risk Score to Predict In-hospital Mortality from COVID-19 in South Korea. *J Korean Med Sci*. 2021;36(15):e108. Published 2021 Apr 19. doi:10.3346/jkms.2021.36.e108.
16. Hohl CM, RosychUnited Kingdom RJ, Archambault PM, O’Sullivan F, Leeies M, Mercier E, et al. DERIVATION AND VALIDATION OF A CLINICAL SCORE TO PREDICT DEATH AMONG NON-PALLIATIVE COVID-19 PATIENTS PRESENTING TO EMERGENCY DEPARTMENTS: THE CCEDRRN COVID MORTALITY SCORE. MedRxiv. 2021. doi: 10.1101/2021.07.28.21261283.
17. Hu C, Liu Z, Jiang Y, Zhang X, Shi O, Xu K, et al. Early prediction of mortality risk among severe COVID-19 patients using machine learning. Int J Epidemiol. 2021 Jan 23;49(6):1918-1929. doi: 10.1093/ije/dyaa171.
18. Ikemura K, Bellin E, Yagi Y, et al. Using Automated Machine Learning to Predict the Mortality of Patients With COVID-19: Prediction Model Development Study. *J Med Internet Res*. 2021;23(2):e23458. Published 2021 Feb 26. doi:10.2196/23458.
19. Incerti D, Rizzo S, Li X, Lindsay L, Yau V, Kleeber D, et al. Risk factors for mortality among hospitalized patients with COVID-19. Medrxiv. 2020. doi: 10.1101/2020.09.22.20196204.
20. Ismail K, Bensasi H, Taha A, et al. Characteristics and outcome of critically ill patients with coronavirus disease-2019 (COVID-19) pneumonia admitted to a tertiary care center in the United Arab Emirates during the first wave of the SARS-CoV-2

pandemic. A retrospective analysis. *PLoS One*. 2021;16(10):e0251687. Published 2021 Oct 22. doi:10.1371/journal.pone.0251687.

1. Ji D, Zhang D, Xu J, et al. Prediction for Progression Risk in Patients With COVID-19 Pneumonia: The CALL Score. *Clin Infect Dis*. 2020;71(6):1393-1399. doi:10.1093/cid/ciaa414.
2. Jimenez-Solem E, Petersen TS, Hansen C, et al. Developing and validating COVID-19 adverse outcome risk prediction models from a bi-national European cohort of 5594 patients. *Sci Rep*. 2021;11(1):3246. Published 2021 Feb 5. doi:10.1038/s41598- 021-81844-x.
3. Jiwa N, Mutneja R, Henry L, Fiscus G, Zu Wallack R. Development of a brief scoring system to predict any-cause mortality in patients hospitalized with COVID-19 infection. *PLoS One*. 2021;16(7):e0254580. Published 2021 Jul 16. doi:10.1371/journal.pone.0254580.
4. Kabootari M, Habibi Tirtashi R, Hasheminia M, et al. Clinical features, risk factors and a prediction model for in-hospital mortality among diabetic patients infected with COVID-19: data from a referral centre in Iran. *Public Health*. 2022;202:84-92. doi:10.1016/j.puhe.2021.11.007.
5. Kamran F, Tang S, Otles E, et al. Early identification of patients admitted to hospital for COVID-19 at risk of clinical deterioration: model development and multisite external validation study. *BMJ*. 2022;376:e068576. Published 2022 Feb 17. doi:10.1136/bmj-2021-068576.
6. Kapoor M, Panda PK, Saini LK, Bahurupi Y. A Retrospective analysis of DIC score and SIC score in prediction of COVID-19 severity. MedRxiv. 2021. doi: https://doi.org/10.1101/2021.06.26.21259369.
7. Kar S, Chawla R, Haranath SP, et al. Multivariable mortality risk prediction using machine learning for COVID-19 patients at admission (AICOVID). *Sci Rep*. 2021;11(1):12801. Published 2021 Jun 17. doi:10.1038/s41598-021-92146-7.
8. Karthikeyan A, Garg A, Vinod PK, Priyakumar UD. Machine Learning Based Clinical Decision Support System for Early COVID-19 Mortality Prediction. *Front Public Health*. 2021;9:626697. Published 2021 May 12. doi:10.3389/fpubh.2021.626697.
9. Katkat F, Karahan S, Varol S, Kalyoncuoglu M, Okuyan E. Mortality prediction with CHA2DS2-VASc, CHA2DS2-VASc-HS and R2CHA2DS2-VASc score in patients hospitalized due to COVID-19. *Eur Rev Med Pharmacol Sci*. 2021;25(21):6767-6774. doi:10.26355/eurrev_202111_27121.
10. Kazemi MA, Ghanaati H, Moradi B, Chavoshi M, Hashemi H, Hemmati S, et al. Prognostic factors of chest CT findings for ICU admission and mortality in patients with COVID-19 pneumonia. Medrxiv. 2020. doi: 10.1101/2020.10.30.20223024
11. Kim I-C, Song JE, Lee HJ, Park J-H, Hyun M, Lee JY, et al. The Implication of Cardiac Injury Score on In-hospital Mortality of Coronavirus Disease 2019. Journal of Korean medical science. 2020;35(39):e349-e.
12. Kivrak M, Guldogan E, Colak C. Prediction of death status on the course of treatment in SARS-COV-2 patients with deep learning and machine learning methods. *Comput Methods Programs Biomed*. 2021;201:105951. doi:10.1016/j.cmpb.2021.105951.
13. Ko H, Chung H, Kang WS, Park C, Kim DW, Kim SE, et al. An Artificial Intelligence Model to Predict the Mortality of COVID-19 Patients at Hospital Admission Time Using Routine Blood Samples: Development and Validation of an Ensemble Model. Journal of medical Internet research. 2020;22(12):e25442-e.
14. Kundi H, Çetin EHÖ, Canpolat U, et al. The role of Frailty on Adverse Outcomes Among Older Patients with COVID-19. *J Infect*. 2020;81(6):944-951. doi:10.1016/j.jinf.2020.09.029.
15. Leoni MLG, Lombardelli L, Colombi D, et al. Prediction of 28-day mortality in critically ill patients with COVID-19: Development and internal validation of a clinical prediction model. *PLoS One*. 2021;16(7):e0254550. Published 2021 Jul 13. doi:10.1371/journal.pone.0254550.
16. Levy TJ, Richardson S, Coppa K, Barnaby DP, McGinn T, Becker LB, et al. Development and validation of a survival calculator for hospitalized patients with COVID-19. Medrxiv. 2020. doi:10.1101/2020.04.22.20075416.
17. Li L, Fang X, Cheng L, et al. Development and validation of a prognostic nomogram for predicting in-hospital mortality of COVID-19: a multicenter retrospective cohort study of 4086 cases in China. *Aging (Albany NY)*. 2021;13(3):3176-3189. doi:10.18632/aging.202605.
18. Li P, Chen L, Liu Z, Pan J, Zhou D, Wang H, et al. Clinical features and short- term outcomes of elderly patients with COVID-19. International Journal of Infectious Diseases. 2020;97:245-50.
19. Liang W, Yao J, Chen A, et al. Early triage of critically ill COVID-19 patients using deep learning. *Nat Commun*. 2020;11(1):3543. Published 2020 Jul 15. doi:10.1038/s41467-020-17280-8.
20. Liang W, Liang H, Ou L, Chen B, Chen A, Li C, et al. Development and validation of a clinical risk score to predict the occurrence of critical illness in hospitalized patients with COVID-19. JAMA Internal Medicine. 2020;180(8):1081-9.
21. Lin JK, Chien TW, Wang LY, Chou W. An artificial neural network model to predict the mortality of COVID-19 patients using routine blood samples at the time of hospital admission: Development and validation study. Medicine (Baltimore). 2021;100(28):e26532.
22. Liu Z, Li J, Li M, et al. Elevated α-hydroxybutyrate dehydrogenase as an independent prognostic factor for mortality in hospitalized patients with COVID-19. *ESC Heart Fail*. 2021;8(1):644-651. doi:10.1002/ehf2.13151.
23. Liu QQ, Cheng A, Wang Y, et al. Cytokines and their relationship with the severity and prognosis of coronavirus disease 2019 (COVID-19): a retrospective cohort study. *BMJ Open*. 2020;10(11):e041471. Published 2020 Nov 30. doi:10.1136/bmjopen- 2020-041471.
24. Liu C, Li L, Song K, et al. A nomogram for predicting mortality in patients with COVID-19 and solid tumors: a multicenter retrospective cohort study. *J Immunother Cancer*. 2020;8(2):e001314. doi:10.1136/jitc-2020-001314.
25. Liu Y, Gao W, Guo W, et al. Prominent coagulation disorder is closely related to inflammatory response and could be as a prognostic indicator for ICU patients with COVID-19. *J Thromb Thrombolysis*. 2020;50(4):825-832. doi:10.1007/s11239-020- 02174-9.
26. Llanera DK, Wilmington R, Shoo H, et al. Clinical Characteristics of COVID-19 Patients in a Regional Population With Diabetes Mellitus: The ACCREDIT Study. *Front Endocrinol (Lausanne)*. 2022;12:777130. Published 2022 Jan 13. doi:10.3389/fendo.2021.777130.
27. López-Escobar A, Madurga R, Castellano JM, et al. Hemogram as marker of in- hospital mortality in COVID-19. *J Investig Med*. 2021;69(5):962-969. doi:10.1136/jim- 2021-001810.
28. Lorente L, Martín MM, González-Rivero AF, et al. DNA and RNA Oxidative Damage and Mortality of Patients With COVID-19. *Am J Med Sci*. 2021;361(5):585-590. doi:10.1016/j.amjms.2021.02.012.
29. Lu X, Jiang L, Chen T, et al. Continuously available ratio of SpO2/FiO2 serves as a noninvasive prognostic marker for intensive care patients with COVID-19. *Respir Res*. 2020;21(1):194. Published 2020 Jul 22. doi:10.1186/s12931-020-01455-4.
30. Lu J, Hu S, Fan R, Liu Z, Yin X, Wang Q, et al. ACP risk grade: a simple mortality index for patients with confirmed or suspected severe acute respiratory syndrome coronavirus 2 disease (COVID-19) during the early stage of outbreak in Wuhan, China. Preprints with The Lancet. 2020.
31. Luo M, Liu J, Jiang W, Yue S, Liu H, Wei S. IL-6 and CD8+ T cell counts combined are an early predictor of in-hospital mortality of patients with COVID-19. *JCI Insight*. 2020;5(13):e139024. Published 2020 Jul 9. doi:10.1172/jci.insight.139024.
32. Ma X, Wang H, Huang J, et al. A nomogramic model based on clinical and laboratory parameters at admission for predicting the survival of COVID-19 patients. *BMC Infect Dis*. 2020;20(1):899. Published 2020 Nov 30. doi:10.1186/s12879-020- 05614-2.
33. Ma X, Ng M, Xu S, et al. Development and validation of prognosis model of mortality risk in patients with COVID-19. *Epidemiol Infect*. 2020;148:e168. Published 2020 Aug 4. doi:10.1017/S0950268820001727.
34. Machado-Alba JE, Valladales-Restrepo LF, Machado-Duque ME, et al. Factors associated with admission to the intensive care unit and mortality in patients with COVID-19, Colombia. *PLoS One*. 2021;16(11):e0260169. Published 2021 Nov 19. doi:10.1371/journal.pone.0260169.
35. Magro B, Zuccaro V, Novelli L, et al. Predicting in-hospital mortality from Coronavirus Disease 2019: A simple validated app for clinical use. *PLoS One*. 2021;16(1):e0245281. Published 2021 Jan 14. doi:10.1371/journal.pone.0245281.
36. Mahdavi M, Choubdar H, Zabeh E, et al. A machine learning based exploration of COVID-19 mortality risk. *PLoS One*. 2021;16(7):e0252384. Published 2021 Jul 2. doi:10.1371/journal.pone.0252384.
37. Mancilla-Galindo J, Vera-Zertuche JM, Navarro-Cruz AR, et al. Development and validation of the patient history COVID-19 (PH-Covid19) scoring system: a multivariable prediction model of death in Mexican patients with COVID-19. *Epidemiol Infect*. 2020;148:e286. Published 2020 Nov 26. doi:10.1017/S0950268820002903.
38. Mann CZ, Abshire C, Yost M, et al. Derivation and external validation of a simple risk score to predict in-hospital mortality in patients hospitalized for COVID-19: A multicenter retrospective cohort study. *Medicine (Baltimore)*. 2021;100(40):e27422. doi:10.1097/MD.0000000000027422.
39. Manocha KK, Kirzner J, Ying X, et al. Troponin and Other Biomarker Levels and Outcomes Among Patients Hospitalized With COVID-19: Derivation and Validation of

the HA2T2 COVID-19 Mortality Risk Score. *J Am Heart Assoc*. 2021;10(6):e018477. doi:10.1161/JAHA.120.018477.

1. Marcolino MS, Pires MC, Ramos LEF, et al. ABC2-SPH risk score for in-hospital mortality in COVID-19 patients: development, external validation and comparison with other available scores. *Int J Infect Dis*. 2021;110:281-308. doi:10.1016/j.ijid.2021.07.049.
2. Martínez-Lacalzada M, Viteri-Noël A, Manzano L, et al. Predicting critical illness on initial diagnosis of COVID-19 based on easily obtained clinical variables: development and validation of the PRIORITY model. *Clin Microbiol Infect*. 2021;27(12):1838-1844. doi:10.1016/j.cmi.2021.07.006.
3. Mayneris-Perxachs J, Russo MF, Ramos R, et al. Blood Hemoglobin Substantially Modulates the Impact of Gender, Morbid Obesity, and Hyperglycemia on COVID-19 Death Risk: A Multicenter Study in Italy and Spain. *Front Endocrinol (Lausanne)*. 2021;12:741248. Published 2021 Nov 2. doi:10.3389/fendo.2021.741248
4. Mei J, Hu W, Chen Q, et al. Development and external validation of a COVID- 19 mortality risk prediction algorithm: a multicentre retrospective cohort study. *BMJ Open*. 2020;10(12):e044028. Published 2020 Dec 24. doi:10.1136/bmjopen-2020- 044028.
5. Mei Q, Wang AY, Bryant A, Yang Y, Li M, Wang F, et al. Development and validation of prognostic model for predicting mortality of COVID-19 patients in Wuhan, China. Sci Rep. 2020;10(1):22451-.
6. Mendizabal M, Ridruejo E, Piñero F, et al. Comparison of different prognostic scores for patients with cirrhosis hospitalized with SARS-CoV-2 infection. *Ann Hepatol*. 2021;25:100350. doi:10.1016/j.aohep.2021.100350.
7. Momeni-Boroujeni A, Mendoza R, Stopard IJ, Lambert B, Zuretti A. A Dynamic Bayesian Model for Identifying High-Mortality Risk in Hospitalized COVID-19 Patients. Infect Dis Rep. 2021;13(1):239-50.
8. Monterde D, Carot-Sans G, Cainzos-Achirica M, Abilleira S, Coca M, Vela E, et al. Comorbidity accounts for severe COVID-19 risk, but how do we measure it? Retrospective assessment of the performance of three measures of comorbidity using 4,607 hospitalizations. MedRxiv. 2021. doi: https://doi.org/10.1101/2021.07.02.21259898.
9. Moulaei K, Shanbehzadeh M, Mohammadi-Taghiabad Z, Kazemi-Arpanahi H. Comparing machine learning algorithms for predicting COVID-19 mortality. *BMC Med*

*Inform Decis Mak*. 2022;22(1):2. Published 2022 Jan 4. doi:10.1186/s12911-021-01742-

0.

1. Murri R, Lenkowicz J, Masciocchi C, et al. A machine-learning parsimonious multivariable predictive model of mortality risk in patients with Covid-19. *Sci Rep*. 2021;11(1):21136. Published 2021 Oct 27. doi:10.1038/s41598-021-99905-6.
2. Nascimento JHP, Costa RLD, Simvoulidis LFN, et al. COVID-19 and Myocardial Injury in a Brazilian ICU: High Incidence and Higher Risk of In-Hospital Mortality. COVID-19 e Injúria Miocárdica em UTI Brasileira: Alta Incidência e Maior Risco de Mortalidade Intra-Hospitalar. *Arq Bras Cardiol*. 2021;116(2):275-282. doi:10.36660/abc.20200671.
3. Néant N, Lingas G, Le Hingrat Q, et al. Modeling SARS-CoV-2 viral kinetics and association with mortality in hospitalized patients from the French COVID cohort. *Proc Natl Acad Sci U S A*. 2021;118(8):e2017962118. doi:10.1073/pnas.2017962118.
4. Nguyen S, Chan R, Cadena J, et al. Budget constrained machine learning for early prediction of adverse outcomes for COVID-19 patients. *Sci Rep*. 2021;11(1):19543. Published 2021 Oct 1. doi:10.1038/s41598-021-98071-z.
5. Nicholson CJ, Wooster L, Sigurslid HH, Li RF, Jiang W, Tian W, et al. Estimating Risk of Mechanical Ventilation and Mortality Among Adult COVID-19 patients Admitted to Mass General Brigham: The VICE and DICE Scores. EClinicalMedicine. 2021;33:100765. doi: https://doi.org/10.1016/j.eclinm.2021.100765.
6. Nuez-Gil IJ, Fernandez-Perez C, Estrada V, Becerra-Munoz VM, El-Battrawy I, Uribarri A, et al. Mortality risk assessment in Spain and Italy, insights of the HOPE COVID-19 registry. Intern Emerg Med. 2020.
7. Obremska M, Pazgan-Simon M, Budrewicz K, et al. Simple demographic characteristics and laboratory findings on admission may predict in-hospital mortality in patients with SARS-CoV-2 infection: development and validation of the COVID-19 score. *BMC Infect Dis*. 2021;21(1):945. Published 2021 Sep 14. doi:10.1186/s12879-021- 06645-z.
8. Oh TK, Song IA. Metformin use and risk of COVID-19 among patients with type II diabetes mellitus: an NHIS-COVID-19 database cohort study. *Acta Diabetol*. 2021;58(6):771-778. doi:10.1007/s00592-020-01666-7
9. Oualim S, Abdeladim S, Ouarradi AE, et al. Elevated levels of D-dimer in patients with COVID-19: prognosis value. *Pan Afr Med J*. 2020;35(Suppl 2):105. Published 2020 Jul 7. doi:10.11604/pamj.supp.2020.35.2.24692.
10. Pan P, Li Y, Xiao Y, et al. Prognostic Assessment of COVID-19 in the Intensive Care Unit by Machine Learning Methods: Model Development and Validation. *J Med Internet Res*. 2020;22(11):e23128. Published 2020 Nov 11. doi:10.2196/23128.
11. Peiró ÓM, Carrasquer A, Sánchez-Gimenez R, et al. Biomarkers and short-term prognosis in COVID-19. *Biomarkers*. 2021;26(2):119-126. doi:10.1080/1354750X.2021.1874052.
12. Philippe A, Chocron R, Gendron N, et al. Circulating Von Willebrand factor and high molecular weight multimers as markers of endothelial injury predict COVID-19 in- hospital mortality. *Angiogenesis*. 2021;24(3):505-517. doi:10.1007/s10456-020-09762- 6.
13. Pigoga JL, Omer YO, Wallis LA. Derivation of a Contextually-Appropriate COVID-19 Mortality Scale for Low-Resource Settings. *Ann Glob Health*. 2021;87(1):31. Published 2021 Mar 26. doi:10.5334/aogh.3278Pigoga.
14. Pimentel MAF, Redfern OC, Hatch R, Young JD, Tarassenko L, Watkinson PJ. Trajectories of vital signs in patients with COVID-19 [published correction appears in Resuscitation. 2021 May;162:91-92]. *Resuscitation*. 2020;156:99-106. doi:10.1016/j.resuscitation.2020.09.002.
15. Plečko D, Bennett N, Mårtensson J, et al. Rapid Evaluation of Coronavirus Illness Severity (RECOILS) in intensive care: Development and validation of a prognostic tool for in-hospital mortality. Acta Anaesthesiol Scand. 2022;66(1):65-75. doi:10.1111/aas.13991.
16. Ponce D, de Andrade LGM, Claure-Del Granado R, Ferreiro-Fuentes A, Lombardi R. Development of a prediction score for in-hospital mortality in COVID-19 patients with acute kidney injury: a machine learning approach. *Sci Rep*. 2021;11(1):24439. Published 2021 Dec 24. doi:10.1038/s41598-021-03894-5.
17. Qeadan F, Mensah NA, Tingey B, Stanford JB. The risk of clinical complications and death among pregnant women with COVID-19 in the Cerner COVID-19 cohort: a retrospective analysis. *BMC Pregnancy Childbirth*. 2021;21(1):305. Published 2021 Apr 16. doi:10.1186/s12884-021-03772-y.
18. Qin ZJ, Liu L, Sun Q, et al. Impaired immune and coagulation systems may be early risk factors for COVID-19 patients: A retrospective study of 118 inpatients from Wuhan, China. *Medicine (Baltimore)*. 2020;99(35):e21700. doi:10.1097/MD.0000000000021700.
19. Qin JJ, Cheng X, Zhou F, et al. Redefining Cardiac Biomarkers in Predicting Mortality of Inpatients With COVID-19. *Hypertension*. 2020;76(4):1104-1112. doi:10.1161/HYPERTENSIONAHA.120.15528.
20. Romualdo LGG, Mulero MDR, Olivo MH, et al. Circulating levels of GDF-15 and calprotectin for prediction of in-hospital mortality in COVID-19 patients: A case series. *J Infect*. 2021;82(2):e40-e42. doi:10.1016/j.jinf.2020.08.010.
21. Rothschild E, Baruch G, Szekely Y, et al. The Predictive Role of Left and Right Ventricular Speckle-Tracking Echocardiography in COVID-19. *JACC Cardiovasc Imaging*. 2020;13(11):2471-2474. doi:10.1016/j.jcmg.2020.07.026.
22. Ryan L, Lam C, Mataraso S, et al. Mortality prediction model for the triage of COVID-19, pneumonia, and mechanically ventilated ICU patients: A retrospective study. *Ann Med Surg (Lond)*. 2020;59:207-216. doi:10.1016/j.amsu.2020.09.044.
23. Saldi SRF, Safitri ED, Setiati S, et al. Prognostic Scoring System for Mortality of Hospitalized COVID-19 Patients in Resource-Limited Settings: A Multicenter Study from COVID-19 Referral Hospitals. *Acta Med Indones*. 2021;53(4):407-415.
24. Sankaranarayanan S, Balan J, Walsh JR, et al. COVID-19 Mortality Prediction From Deep Learning in a Large Multistate Electronic Health Record and Laboratory Information System Data Set: Algorithm Development and Validation. *J Med Internet Res*. 2021;23(9):e30157. Published 2021 Sep 28. doi:10.2196/30157.
25. Sauzay C, Couillez G, Le Guyader M, et al. A simple score (Biovid-19) based on biological parameters predicts transfer to intensive care units and death in COVID-19 patients. *Curr Res Transl Med*. 2021;69(2):103284. doi:10.1016/j.retram.2021.103284.
26. Schlauch D, Fisher AM, Correia J, Fu X, Martin C, Junglen A, et al. Development of a Real-Time Risk Model (RTRM) for Predicting In-Hospital COVID-19 Mortality. MedRxiv. 2021. doi: https://doi.org/10.1101/2021.04.26.21256138.
27. Schöning V, Liakoni E, Baumgartner C, et al. Development and validation of a prognostic COVID-19 severity assessment (COSA) score and machine learning models for patient triage at a tertiary hospital. *J Transl Med*. 2021;19(1):56. Published 2021 Feb 5. doi:10.1186/s12967-021-02720-w.
28. Sensusiati AD, Amin M, Nasronudin N, et al. Age, neutrophil lymphocyte ratio, and radiographic assessment of the quantity of lung edema (RALE) score to predict in- hospital mortality in COVID-19 patients: a retrospective study. *F1000Res*. 2020;9:1286. Published 2020 Oct 30. doi:10.12688/f1000research.26723.2.
29. Shah SA, Moore E, Robertson C, et al. Predicted COVID-19 positive cases, hospitalisations, and deaths associated with the Delta variant of concern, June-July, 2021. *Lancet Digit Health*. 2021;3(9):e539-e541. doi:10.1016/S2589-7500(21)00175-8
30. Shamout FE, Shen Y, Wu N, et al. An artificial intelligence system for predicting the deterioration of COVID-19 patients in the emergency department. *NPJ Digit Med*. 2021;4(1):80. Published 2021 May 12. doi:10.1038/s41746-021-00453-0
31. Shang Y, Liu T, Wei Y, Li J, Shao L, Liu M, et al. Scoring systems for predicting mortality for severe patients with COVID-19. EClinicalMedicine. 2020;24:100426-.
32. Shang M, Wei J, Zou HD, Zhou QS, Zhang YT, Wang CY. Early Warning Factors of Death in COVID-19 Patients. *Curr Med Sci*. 2021;41(1):69-76. doi:10.1007/s11596- 021-2320-7.
33. Shao F, Xu S, Ma X, et al. In-hospital cardiac arrest outcomes among patients with COVID-19 pneumonia in Wuhan, China. *Resuscitation*. 2020;151:18-23. doi:10.1016/j.resuscitation.2020.04.005.
34. Shayganfar A, Sami R, Sadeghi S, et al. Risk factors associated with intensive care unit (ICU) admission and in-hospital death among adults hospitalized with COVID- 19: a two-center retrospective observational study in tertiary care hospitals [published correction appears in Emerg Radiol. 2021 Dec;28(6):1231]. *Emerg Radiol*. 2021;28(4):691-697. doi:10.1007/s10140-021-01903-8.
35. Singh V, Kamaleswaran R, Chalfin D, et al. A deep learning approach for predicting severity of COVID-19 patients using a parsimonious set of laboratory markers. *iScience*. 2021;24(12):103523. doi:10.1016/j.isci.2021.103523.
36. Sinkovits G, Mező B, Réti M, et al. Complement Overactivation and Consumption Predicts In-Hospital Mortality in SARS-CoV-2 Infection. *Front Immunol*. 2021;12:663187. Published 2021 Mar 25. doi:10.3389/fimmu.2021.663187.
37. Sîrbu A, Barbieri G, Faita F, et al. Early outcome detection for COVID-19 patients. *Sci Rep*. 2021;11(1):18464. Published 2021 Sep 16. doi:10.1038/s41598-021- 97990-1.
38. Soto-Mota A, Marfil-Garza BA, Castiello-de Obeso S, et al. Prospective predictive performance comparison between clinical gestalt and validated COVID-19 mortality scores. *J Investig Med*. 2022;70(2):415-420. doi:10.1136/jim-2021-002037.
39. Soto-Mota A, Marfil-Garza BA, Martínez Rodríguez E, Barreto Rodríguez JO, López Romo AE, Alberti Minutti P, et al. The low-harm score for predicting mortality in

patients diagnosed with COVID-19: A multicentric validation study. J Am Coll Emerg Physicians Open. 2020.

1. Sottile PD, Albers D, DeWitt PE, et al. Real-Time Electronic Health Record Mortality Prediction During the COVID-19 Pandemic: A Prospective Cohort Study. Preprint. *medRxiv*. 2021;2021.01.14.21249793. Published 2021 Jan 15. doi:10.1101/2021.01.14.21249793.
2. Sottile PD, Albers D, DeWitt PE, Russell S, Stroh JN, Kao DP, et al. Real-Time Electronic Health Record Mortality Prediction During the COVID-19 Pandemic: A Prospective Cohort Study. J Am Med Inform Assoc. 2021.
3. Sourij H, Aziz F, Bräuer A, Ciardi C, Clodi M, Fasching P, et al. COVID‐19 fatality prediction in people with diabetes and prediabetes using a simple score upon hospital admission. Diabetes, Obesity and Metabolism. 2020;23:589-98.
4. Stachel A, Daniel K, Ding D, Francois F, Phillips M, Lighter J. Development and validation of a machine learning model to predict mortality risk in patients with COVID-

19. BMJ Health Care Inform. 2021;28(1).

1. Sun C, Hong S, Song M, Li H, Wang Z. Predicting COVID-19 disease progression and patient outcomes based on temporal deep learning. *BMC Med Inform Decis Mak*. 2021;21(1):45. Published 2021 Feb 8. doi:10.1186/s12911-020-01359-9.
2. Surme S, Buyukyazgan A, Bayramlar OF, et al. Predictors of Intensive Care Unit Admission or Mortality in Patients with Coronavirus Disease 2019 Pneumonia in Istanbul, Turkey. *Jpn J Infect Dis*. 2021;74(5):458-464. doi:10.7883/yoken.JJID.2020.1065.
3. Synolaki E, Papadopoulos V, Divolis G, et al. The Activin/Follistatin Axis Is Severely Deregulated in COVID-19 and Independently Associated With In-Hospital Mortality. *J Infect Dis*. 2021;223(9):1544-1554. doi:10.1093/infdis/jiab108.
4. Thomson RJ, Hunter J, Dutton J, et al. Clinical characteristics and outcomes of critically ill patients with COVID-19 admitted to an intensive care unit in London: A prospective observational cohort study. *PLoS One*. 2020;15(12):e0243710. Published 2020 Dec 15. doi:10.1371/journal.pone.0243710.
5. Turrini M, Gardellini A, Beretta L, Buzzi L, Ferrario S, Vasile S, et al. Clinical Course and Risk Factors for In-Hospital Mortality of 205 Patients with SARS-CoV-2 Pneumonia in Como, Lombardy Region, Italy. Vaccines (Basel). 2021;9(6).
6. Vagliano I, Brinkman S, Abu-Hanna A, et al. Can we reliably automate clinical prognostic modelling? A retrospective cohort study for ICU triage prediction of in-

hospital mortality of COVID-19 patients in the Netherlands. *Int J Med Inform*. 2022;160:104688. doi:10.1016/j.ijmedinf.2022.104688.

1. Van Dam PM, Zelis N, Stassen P, van Twist DJL, De Leeuw PW, van Kuijk S, et al. Validating the RISE UP score for predicting prognosis in patients with COVID-19 in the emergency department: a retrospective study. BMJ Open. 2021;11(2):e045141.
2. Varol Y, Hakoglu B, Kadri Cirak A, et al. The impact of charlson comorbidity index on mortality from SARS-CoV-2 virus infection and A novel COVID-19 mortality index: CoLACD. *Int J Clin Pract*. 2021;75(4):e13858. doi:10.1111/ijcp.13858.
3. Vela E, Carot-Sans G, Clèries M, Monterde D, Acebes X, Comella A, et al. Development and performance of a population-based risk stratification model for COVID-19. MedRxiv. 2021. doi: https://doi.org/10.1101/2021.05.25.21257783.
4. Velasco-Rodríguez D, Alonso-Dominguez JM, Vidal Laso R, et al. Development and validation of a predictive model of in-hospital mortality in COVID-19 patients. *PLoS One*. 2021;16(3):e0247676. Published 2021 Mar 4. doi:10.1371/journal.pone.0247676.
5. Vepa A, Saleem A, Rakhshan K, et al. Using Machine Learning Algorithms to Develop a Clinical Decision-Making Tool for COVID-19 Inpatients. *Int J Environ Res Public Health*. 2021;18(12):6228. Published 2021 Jun 9. doi:10.3390/ijerph18126228.
6. Verma AA, Hora T, Jung HY, et al. Characteristics and outcomes of hospital admissions for COVID-19 and influenza in the Toronto area. *CMAJ*. 2021;193(12):E410- E418. doi:10.1503/cmaj.202795.
7. Vicka V, Januskeviciute E, Miskinyte S, et al. Comparison of mortality risk evaluation tools efficacy in critically ill COVID-19 patients. *BMC Infect Dis*. 2021;21(1):1173. Published 2021 Nov 22. doi:10.1186/s12879-021-06866-2.
8. Villa E, Critelli R, Lasagni S, et al. Dynamic angiopoietin-2 assessment predicts survival and chronic course in hospitalized patients with COVID-19. *Blood Adv*. 2021;5(3):662-673. doi:10.1182/bloodadvances.2020003736.
9. Wang P, Sha J, Meng M, et al. Risk factors for severe COVID-19 in middle-aged patients without comorbidities: a multicentre retrospective study. *J Transl Med*. 2020;18(1):461. Published 2020 Dec 7. doi:10.1186/s12967-020-02655-8
10. Wang F, Ran L, Qian C, et al. Epidemiology and Outcomes of Acute Kidney Injury in COVID-19 Patients with Acute Respiratory Distress Syndrome: A Multicenter Retrospective Study. *Blood Purif*. 2021;50(4-5):499-505. doi:10.1159/000512371.
11. Wang K, Zuo P, Liu Y, et al. Clinical and Laboratory Predictors of In-hospital Mortality in Patients With Coronavirus Disease-2019: A Cohort Study in Wuhan, China. *Clin Infect Dis*. 2020;71(16):2079-2088. doi:10.1093/cid/ciaa538.
12. Wang S, Sheng Y, Tu J, Zhang L. Association between peripheral lymphocyte count and the mortality risk of COVID-19 inpatients. *BMC Pulm Med*. 2021;21(1):55. Published 2021 Feb 11. doi:10.1186/s12890-021-01422-9.
13. Wang JM, Liu W, Chen X, McRae MP, McDevitt JT, Fenyö D. Predictive Modeling of Morbidity and Mortality in Patients Hospitalized With COVID-19 and its Clinical Implications: Algorithm Development and Interpretation. *J Med Internet Res*. 2021;23(7):e29514. Published 2021 Jul 9. doi:10.2196/29514.
14. Wang J, Zhang H, Qiao R, Ge Q, Zhang S, Zhao Z, et al. Thrombo-inflammatory features predicting mortality in patients with COVID-19: The FAD-85 score. Journal of International Medical Research. 2020;48(9):1-14.
15. Wang S, Dong D, Li L, et al. A Deep Learning Radiomics Model to Identify Poor Outcome in COVID-19 Patients With Underlying Health Conditions: A Multicenter Study. *IEEE J Biomed Health Inform*. 2021;25(7):2353-2362. doi:10.1109/JBHI.2021.3076086.
16. Weng Z, Chen Q, Li S, Li H, Zhang Q, Lu S, et al. ANDC: an early warning score to predict mortality risk for patients with Coronavirus Disease 2019. Journal of translational medicine. 2020;18(328):1-10.
17. Williams RD, Markus AF, Yang C, Salles TD, DuVall SL, Falconer T, et al. Seek COVER: Development and validation of a personalized risk calculator for COVID-19 outcomes in an international network. Medrxiv. 2020. doi: https://doi.org/10.1101/2020.05.26.20112649.
18. Wongvibulsin S, Garibaldi BT, Antar AAR, et al. Development of Severe COVID-19 Adaptive Risk Predictor (SCARP), a Calculator to Predict Severe Disease or Death in Hospitalized Patients With COVID-19. *Ann Intern Med*. 2021;174(6):777-785. doi:10.7326/M20-6754.
19. Wu G, Yang P, Xie Y, Woodruff HC, Rao X, Guiot J, et al. Development of a clinical decision support system for severity risk prediction and triage of COVID-19 patients at hospital admission: an international multicentre study. Eur Respir J. 2020;56(2).
20. Xie J, Hungerford D, Chen H, Abrams ST, Li S, Wang G, et al. Development and external validation of a prognostic multivariable model on admission for hospitalized

patients with COVID-19. PLOS ONE 16(8): e0255748.. 2020. doi: https://doi.org/10.1371/journal.pone.0255748.

1. Yan L, Zhang H-T, Goncalves J, Xiao Y, Wang M, Guo Y, et al. An interpretable mortality prediction model for COVID-19 patients. Nat Mach Intell. 2020;2:283-8.
2. Yasukawa K, Minami T, Boulware DR, Shimada A, Fischer EA. Point-of-Care Lung Ultrasound for COVID-19: Findings and Prognostic Implications From 105 Consecutive Patients. *J Intensive Care Med*. 2021;36(3):334-342. doi:10.1177/0885066620988831.
3. Genc Yavuz B, Colak S, Guven R, Altundag İ, Seyhan AU, Gunay Inanc R. Clinical Features of the 60 Years and Older Patients Infected with 2019 Novel Coronavirus: Can We Predict Mortality Earlier?. *Gerontology*. 2021;67(4):433-440. doi:10.1159/000514481.
4. Yoo E, Percha B, Tomlinson M, RazUnited Kingdom V, Pan S, Basist M, et al. Development and calibration of a simple mortality risk score for hospitalized COVID-19 adults. Medrxiv. 2020. doi: https://doi.org/10.1101/2020.08.31.20185363.
5. Yu Z, Ke Y, Xie J, et al. Clinical characteristics on admission predict in-hospital fatal outcome in patients aged ≥75 years with novel coronavirus disease (COVID-19): a retrospective cohort study. *BMC Geriatr*. 2020;20(1):514. Published 2020 Nov 30. doi:10.1186/s12877-020-01921-0.
6. Yu L, Halalau A, Dalal B, Abbas AE, Ivascu F, Amin M, et al. Machine learning methods to predict mechanical ventilation and mortality in patients with COVID-19. PLoS One. 2021;16(4):e0249285.
7. Zayat R, Kalverkamp S, Grottke O, et al. Role of extracorporeal membrane oxygenation in critically Ill COVID-19 patients and predictors of mortality. *Artif Organs*. 2021;45(6):E158-E170. doi:10.1111/aor.13873.
8. Zeng Z, Wu C, Lin Z, et al. Development and validation of a simple-to-use nomogram to predict the deterioration and survival of patients with COVID-19. *BMC Infect Dis*. 2021;21(1):356. Published 2021 Apr 16. doi:10.1186/s12879-021-06065-z
9. Zhang H, Shi T, Wu X, Zhang X, Wang K, Bean D, et al. Risk prediction for poor outcome and death in hospital in-patients with COVID-19: derivation in Wuhan, China and external validation in London, United Kingdom. Preprints with The Lancet. 2020.
10. Zhang S, Xu Y, Li J, et al. Symptomless multi-variable apnea prediction index assesses adverse outcomes in patients with Corona Virus Disease 2019. *Sleep Med*. 2020;75:294-300. doi:10.1016/j.sleep.2020.08.031.
11. Zhang S, Guo M, Duan L, Wu F, Hu G, Wang Z, et al. Development and validation of a risk factor-based system to predict short-term survival in adult hospitalized patients with COVID-19: a multicenter, retrospective, cohort study. Critical Care. 2020;24(1):1-13.
12. Zhang H, Shi T, Wu X, et al. Risk prediction for poor outcome and death in hospital in-patients with COVID-19: derivation in Wuhan, China and external validation in London, UK. medRxiv 2020.04.28.20082222; doi: https://doi.org/10.1101/2020.04.28.20082222.
13. Zhao Z, Chen A, Hou W, Graham JM, Li H, Richman PS, et al. Prediction model and risk scores of ICU admission and mortality in COVID-19. PLoS One. 2020;15(7):e0236618.
14. Zhou F, Yu T, Du R, Fan G, Liu Y, Liu Z, et al. Clinical course and risk factors for mortality of adult inpatients with COVID-19 in Wuhan, China: a retrospective cohort study. The Lancet. 2020;395(10229):1054-62.
15. Zhou J, Huang L, Chen J, Yuan X, Shen Q, Dong S, et al. Clinical features predicting mortality risk in older patients with COVID-19. Current Medical Research and Opinion. 2020;36(11):1753-9.
16. Zhu JS, Ge P, Jiang C, Zhang Y, Li X, Zhao Z, et al. Deep-learning artificial intelligence analysis of clinical variables predicts mortality in COVID-19 patients. J Am Coll Emerg Physicians Open. 2020.
17. Zinellu A, Arru F, De Vito A, et al. The De Ritis ratio as prognostic biomarker of in-hospital mortality in COVID-19 patients. *Eur J Clin Invest*. 2021;51(1):e13427. doi:10.1111/eci.13427.
